# Rac1 promotes kidney collecting duct repair by mechanically coupling cell morphology to mitotic entry

**DOI:** 10.1126/sciadv.adi7840

**Published:** 2024-02-07

**Authors:** Fabian Bock, Xinyu Dong, Shensen Li, Olga M. Viquez, Eric Sha, Matthew Tantengco, Elizabeth M. Hennen, Erin Plosa, Alireza Ramezani, Kyle L. Brown, Young Mi Whang, Andrew S. Terker, Juan Pablo Arroyo, David G. Harrison, Agnes Fogo, Cord H. Brakebusch, Ambra Pozzi, Roy Zent

**Affiliations:** ^1^ Division of Nephrology and Hypertension, Department of Medicine, Vanderbilt University Medical Center, Nashville, TN, USA.; ^2^ Department of Veterans Affairs Hospital, Tennessee Valley Healthcare System, Nashville, TN, USA.; ^3^ Vanderbilt Center for Kidney Disease, Vanderbilt University Medical Center, Nashville, TN, USA.; ^4^ Department of Biomedical Engineering, Vanderbilt University, Nashville, TN, USA.; ^5^ Division of Neonatology, Department of Pediatrics, Vanderbilt University Medical Center, Nashville, TN, USA.; ^6^ Interdisciplinary Center for Quantitative Modeling in Biology, University of California, Riverside, CA, USA.; ^7^ Department of Physics and Astronomy, University of California, Riverside, CA, USA.; ^8^ Division of Clinical Pharmacology, Department of Medicine, Vanderbilt University Medical Center, Nashville, TN, USA.; ^9^ Department of Pathology, Microbiology and Immunology, Vanderbilt University Medical Center, Nashville, TN, USA.; ^10^ Biotech Research Center, University of Copenhagen, Copenhagen DK-2200, Denmark.; ^11^ Department of Physiology and Molecular Biophysics, Vanderbilt University School of Medicine, Nashville, TN, USA.; ^12^ Department of Cell and Developmental Biology, Vanderbilt University School of Medicine, Nashville, TN, USA.

## Abstract

Prolonged obstruction of the ureter, which leads to injury of the kidney collecting ducts, results in permanent structural damage, while early reversal allows for repair. Cell structure is defined by the actin cytoskeleton, which is dynamically organized by small Rho guanosine triphosphatases (GTPases). In this study, we identified the Rho GTPase, Rac1, as a driver of postobstructive kidney collecting duct repair. After the relief of ureteric obstruction, Rac1 promoted actin cytoskeletal reconstitution, which was required to maintain normal mitotic morphology allowing for successful cell division. Mechanistically, Rac1 restricted excessive actomyosin activity that stabilized the negative mitotic entry kinase Wee1. This mechanism ensured mechanical G_2_-M checkpoint stability and prevented premature mitotic entry. The repair defects following injury could be rescued by direct myosin inhibition. Thus, Rac1-dependent control of the actin cytoskeleton integrates with the cell cycle to mediate kidney tubular repair by preventing dysmorphic cells from entering cell division.

## Teaser

The small Rho GTPase Rac1 promotes the kidney collecting duct repair by maintaining actin-dependent cell shape during division.

## INTRODUCTION

Obstructive uropathy is severe kidney damage that results from functional or structural restriction of urinary flow (*1*, *2*). Obstructive uropathy is common and accounts for nearly 10% of all acute and chronic kidney disease cases in the general population (*1*). The buildup of pressure within the urinary collecting system leads to mechanically induced collecting duct (CD) dilatation with flattening, atrophy, and apoptosis of CD epithelial cells (*3*, *4*). Without timely decompression, irreversible progressive renal injury requiring dialysis often ensues (*5*, *6*). Despite notable diagnostic and procedural advances in recognizing and relieving urinary obstruction, the outcomes for patients after obstructive uropathy have not improved (*7*). This is due to a gap in understanding how the CD repairs and a lack of targeted molecular interventions.

Mechanisms of renal tubular epithelial repair have primarily been studied in the proximal tubule exposed to ischemic or toxic injury (*8*). These kidney epithelial cells are largely quiescent at baseline but rapidly reenter the cell cycle when injured and replace the cells that died by a process of dedifferentiation (*8*). Arrest in the G_2_-M phase of the cell cycle is a well-described maladaptive reaction of proximal tubule injury leading to epithelial cell senescence that promotes local inflammation and fibrosis (*8*). Similar to the proximal tubule, the CD cells have notable self-repair capacity mediated by reentry into the cell cycle and increasing cell division following obstruction (*3*). However, the mechanisms of CD repair are poorly defined and likely morphologically and functionally distinct from the proximal tubule.

Kidney epithelial cell repair after injury requires dynamic morphological change mediated by the actin cytoskeleton (*9*). There is substantial actin dysregulation soon after initial tubular injury, and loss of actin organization destroys architecture and hampers tubular resorptive function (*9*, *10*). Consistent with this, animal studies indicate that therapeutic tubular actin cytoskeletal stabilization by enhancing actin branching protects the kidney in various experimental injury models (*11*). Despite these findings, it is unclear what critical cellular functions are required in kidney tubular epithelial repair, whether dynamic actin reorganization plays a role and how this is molecularly coordinated.

Rac1 is a small canonical Rho guanosine triphosphatase (GTPase) that regulates actin cytoskeletal dynamics (*12*). Integrin–extracellular matrix interactions activate Rac1, and activated [guanosine 5′-triphosphate (GTP)–bound] Rac1 polymerizes and organizes the actin cytoskeleton via various downstream effectors to promote lamellipodia formation and cellular migration (*13*). Moreover, in epithelia, Rac1 promotes polarized actin organization and cell-cell junction stability and maintains apicobasal polarity (*14*–*16*). We and others showed that Rac1 is required for normal postdevelopment CD structure and function (*17*, *18*) as it maintains actin cytoskeletal branching at cell-cell junctions that restricts excessive actomyosin activity and maintains cell shape and polarity (*18*). In addition, there is strong evidence that Rac1 function in epithelial cells goes beyond actin organization (*19*). Rac1 stimulates cell cycle progression by activating traditional proliferative cell survival pathways (extracellular signal–regulated kinase, p38–mitogen-activated protein kinase, Akt, and c-Jun N-terminal kinase) via downstream effectors that overlap with its actin cytoskeletal function (e.g., p21-activated protein kinase 1), raising the possibility that the actin organization and cell cycle progression functions of Rac1 are intimately linked (*20*–*23*).

In this study, we analyzed the role of Rac1 in CD repair following irreversible prolonged and short reversible obstructive kidney injury. We show that CDs lacking Rac1 are unable to reconstitute their morphology or proliferate normally after relief of obstruction, as this small GTPase plays a key role in integrating actin cytoskeletal organization and cell cycle control. Mechanistically, we show that Rac1 regulates the orderly actin-dependent progression through G_2_-M that ensures reliable cell division.

## RESULTS

### Rac1 is required to maintain CD integrity during prolonged obstruction

**Fig. 1.**
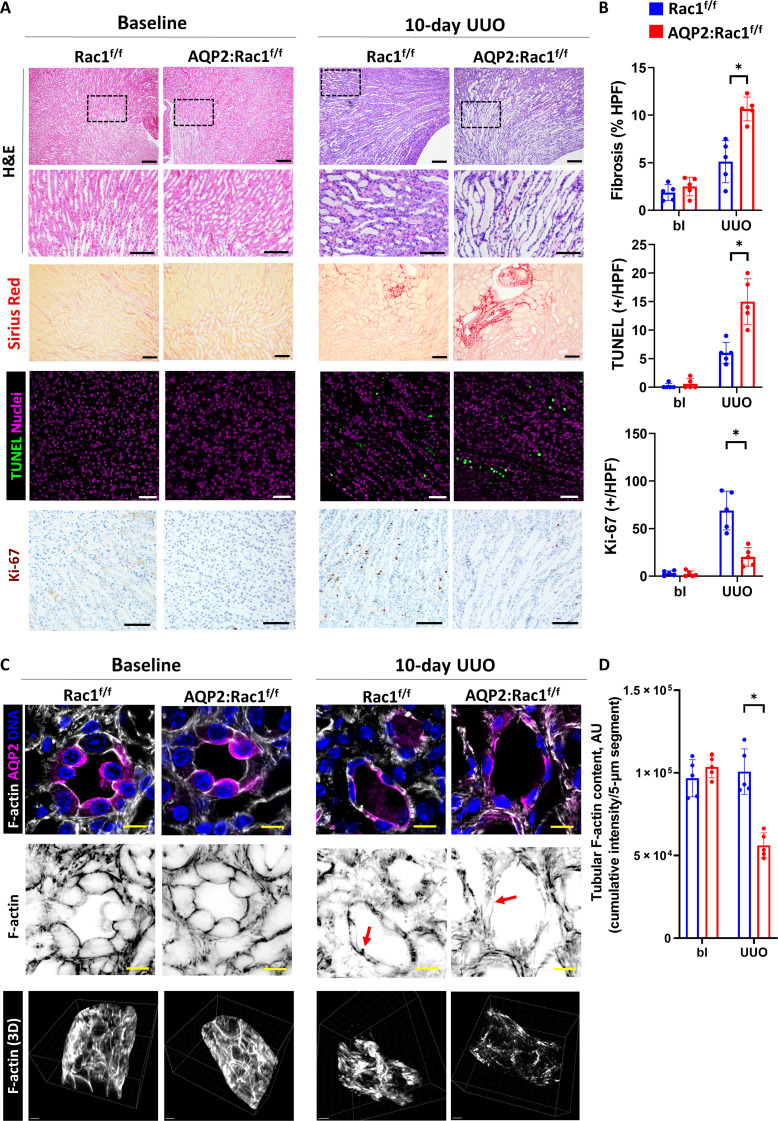
Rac1 is required to maintain epithelial and F-actin integrity during prolonged obstruction. (**A**) Hematoxylin and eosin (H&E)–stained paraffin kidney sections in the first and second rows [scale bars, 100 μm (top row) and 50 μm (insets); area for insets indicated by a black dashed box), Sirius Red staining in the third row (scale bars, 50 μm), apoptosis [terminal deoxynucleotidyl transferase–mediated deoxyuridine triphosphate nick end labeling (TUNEL)] staining in the fourth row (nuclei are magenta, and positive nuclei are green; scale bars, 50 μm), and Ki-67 in the fifth row [3,3′-Diaminobenzidine (DAB), brown; scale bars, 50 μm] of medullary regions of control (Rac1^f/f^) or AQP2:Rac1^f/f^ kidneys at baseline or 10 days after obstruction (10-day UUO, ureteral ligation with sutures). (**B**) Quantification of fibrosis [as percentage of Sirius Red–positive area per high-power field (HPF)], TUNEL (positive cells per HPF), and Ki-67 (positive cells per HPF) from (A). bl, baseline. (**C**) Thick fresh frozen medullary kidney slices stained for AQP2 (CDs) and F-actin of baseline and obstructed (10-day UUO) control and AQP2:Rac1^f/f^ kidneys. First row depicts a cross section (scale bars, 10 μm), second row shows the F-actin channel with the colors inverted (scale bars, 10 μm), and the third row shows 3D F-actin reconstructions (scale bars, 5 μm). Red arrows in the second row highlight basolateral F-actin that is deficient in the injured mutant CDs. (**D**) Quantification of total tubular F-actin content as outlined in the methods. AU, arbitrary units. *n* = 5 mice per group with each dot in the scatter plots representing an individual sample. Bars are means ± SD. *P < 0.05.

To determine whether Rac1 is essential in maintaining collecting system epithelial structural integrity in a model with morphological damage of the CD, we induced prolonged obstruction (>7 days) in mice, which leads to substantial parenchymal loss with apoptosis prevailing over regeneration (*3*). We deleted Rac1 in the CDs at embryonic day 19 (E19) by crossing Rac1^flox/flox^ (Rac1^f/f^) mice with AQP2 (Aquaporin 2)–Cre mice. Successful deletion of Rac1 in the collecting system of AQP2:Rac1^f/f^ mice was confirmed by immunofluorescence of medullary CDs and immunoblotting of papillary lysates (fig. S1). AQP2:Rac1^f/f^ mice did not have any histological abnormalities or functional differences in the kidney at baseline (Fig. 1A and fig. S2). We performed prolonged unilateral ureteral obstruction (UUO) and analyzed the kidneys after 10 days of UUO. As expected, this injury resulted in medullary tubular dilatation with epithelial flattening in Rac1^f/f^ mice, which was more pronounced in the kidneys of AQP2:Rac1^f/f^ mice. In keeping with this finding, the AQP2:Rac1^f/f^ kidneys displayed significantly increased fibrosis (Sirius Red) and medullary apoptosis with decreased proliferation (Ki-67, all active cell cycle phases) (Fig. 1, A and B) compared to Rac1^f/f^ kidneys indicating more severe obstruction-induced epithelial damage. Given the role of Rac1 in regulating actin cytoskeletal organization, we performed thick frozen sectioning of fresh tissue, followed by optical clearing and high-resolution three-dimensional (3D) confocal imaging of the actin cytoskeleton of CDs demarcated by AQP2. There was no difference in epithelial actin structure at baseline between genotypes (Fig. 1C), and after UUO, Rac1^f/f^ CDs demonstrated flattening but a largely preserved pattern of basolateral filamentous actin (F-actin). By contrast the AQP2:Rac1^f/f^ CDs had a near-complete loss of F-actin, which was more obvious in the 3D reconstruction of the tubular actin cytoskeleton (Fig. 1, C and D, and movie S1). There was no general decrease in F-actin intensity surrounding the CDs, suggesting that the F-actin defect in the AQP2:Rac1^f/f^ mice was CD specific (fig. S3). Together, these results show that Rac1 plays a critical role in protecting the kidney collecting system from injury following prolonged obstruction.

### Rac1 promotes CD repair, morphological reconstitution, and F-actin recovery

**Fig. 2.**
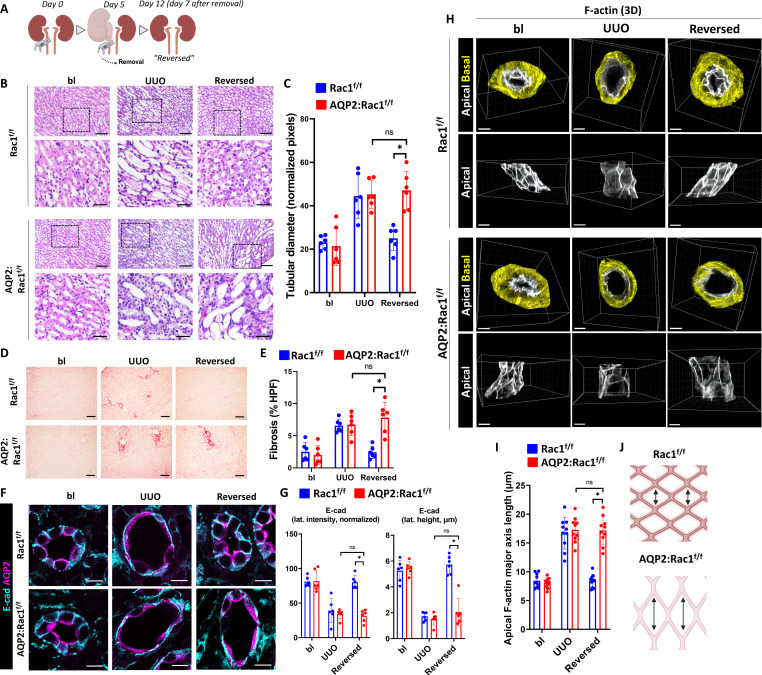
Rac1 promotes CD repair, morphological reconstitution, and F-actin recovery. (**A**) Schematic representation of the reversible UUO model outlining surgical clamp placement and removal for collecting system decompression. (**B**) H&E paraffin kidney sections of Rac1^f/f^ and AQP2:Rac1^f/f^ mice at baseline, after 5 days of UUO and 1 week after reversal of obstruction (“reversed”) in the top row (scale bars, 50 μm) with a black dashed box indicating insets shown below (scale bars, 20 μm). (**C**) Quantification of medullary tubular diameter in (B) in normalized pixels (normalized to frame size that was equal between groups). (**D**) Sirius Red stained kidney sections (scale bars, 100 μm). (**E**) Quantification of fibrosis in (D) as percent Sirius Red positive per HPF. (**F**) E-cadherin immunostaining was performed on paraffin kidney sections of baseline, obstructed, and reversed Rac1^f/f^ and AQP2:Rac1^f/f^ mice, and the CDs were marked by AQP2 (scale bars, 10 μm); sections were analyzed by confocal microscopy. (**G**) Quantification of normalized lateral E-cadherin intensity (left) and lateral E-cadherin height in micrometers (right) from (F) using ImageJ. (**H**) 3D reconstructions of AQP2-positive F-actin–labeled CDs with segmentation and masking of the basolateral (yellow) and apical (white) F-actin using Imaris. Top rows display a top view of both masking channels, and bottom rows show side views of apical F-actin. *n* = 6 mice per group. Scale bars, 5 μm. (**I**) Quantification of the apical F-actin major axis length in micrometers (details in Materials and Methods) with each dot in the scatter plots representing measurements. (**J**) Schematic depiction of the results showing abnormal longitudinal extension of the apical F-actin meshwork of repairing AQP2:Rac1^f/f^ CDs. Bars are means ± SD. ns, not significant. *P < 0.05. Panels (A) and (J) were created with Biorender.com.

We next defined whether Rac1 played a role in CD repair following injury using a well-established model of reversible UUO (*24*–*26*). In this model, microvascular clamps are used instead of sutures for ureteral ligation allowing for successful decompression and injury recovery (Fig. 2A and fig. S4). Kidneys were reversibly obstructed for five days, which is a time point after which damage is still recoverable (*25*). There were no differences in tubular dilatation or fibrosis (assessed by picrosirius red) at 5 days of injury; however, the tubular dilatation and fibrosis did not regress in the reversed AQP2:Rac1^f/f^ mice, suggesting that they were unable to undergo tubular repair (Fig. 2, B to E). To assess whether the CD cells could regain their epithelial integrity following injury, we labeled AQP2^+^ CDs with the basolateral adherens junction marker E-cadherin. There was a loss of the typical basolateral E-cadherin pattern in both groups following obstruction, which was reestablished in repairing Rac1^f/f^ CDs; however, there was persistently decreased E-cadherin height and lateral density in the AQP2:Rac1^f/f^ CDs (Fig. 2, F and G). Medullary tubular dilatation, inflammatory infiltrates, destruction of renal parenchymal architecture, and fibrosis were still present 1 month after reversal of obstruction in the AQP2:Rac1^f/f^ mice, while the Rac1^f/f^ kidneys appeared to be morphologically normal (fig. S5).

To support the structure of a cell, the actin cytoskeleton forms F-actin out of globular actin monomers (*27*). To examine the actin cytoskeleton in greater detail, we performed optical clearing of fresh thick inner medullary slices and performed 3D high-resolution confocal imaging of F-actin. Both Rac1^f/f^ and AQP2:Rac1^f/f^ CDs showed dysmorphic F-actin and diminished apical F-actin density following UUO. This was reversible in the Rac1^f/f^ CDs; however, it persisted in AQP2:Rac1^f/f^ CDs (fig. S6). 3D reconstruction of the phalloidin-labeled actin cytoskeleton, differential segmentation, and masking of the apical versus basolateral F-actin cytoskeleton revealed decreased density and increased elongation of the tubular apical F-actin meshwork in the flattened AQP2:Rac1^f/f^ CD epithelium during repair, whereas the F-actin structure returned to normal in control CDs (Fig. 2, H to J, and movie S2). To verify that Rac1 plays a role for reconstitution of the actin cytoskeleton after injury, we subjected a monolayer of silicon-rhodamine (SiR)-actin–labeled isolated Rac1^f/f^ and Rac1^−/−^CD cells [generated as described in (*18*)] to two-photon laser ablation. Apical F-actin formation in cells surrounding the site of injury was clearly present in Rac1^f/f^ but not Rac1^−/−^ CD cells (fig. S7 and movie S3). In summary, Rac1 is required to reconstitute epithelial F-actin architecture during the repair process both in vivo and in vitro.

### Rac1 is required for CD proliferation and maintains CD cells in G_2_-M during repair

**Fig. 3.**
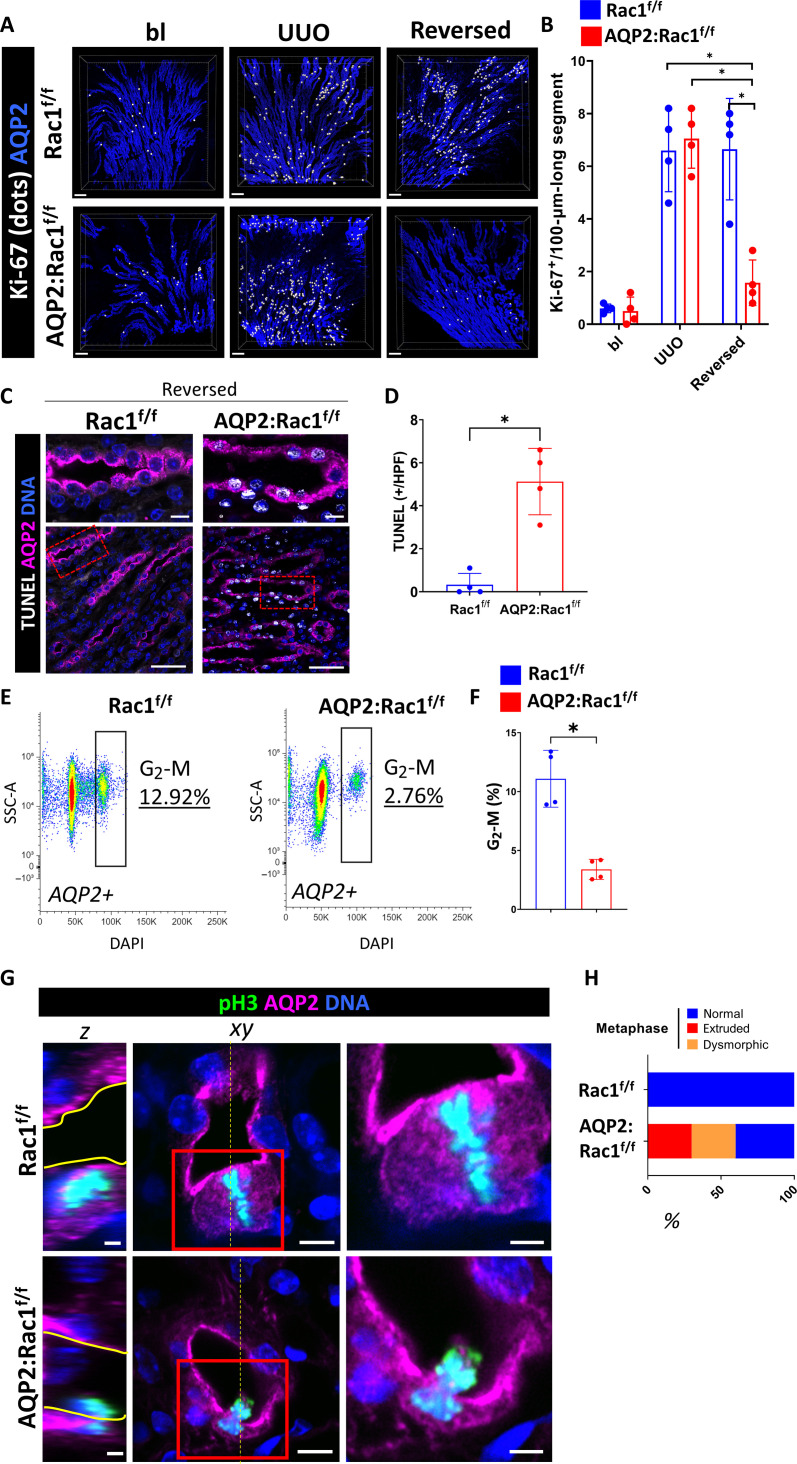
Rac1 promotes proliferation and normal mitotic progression during repair. (**A**) Optically cleared full medullary slices of baseline, obstructed (“UUO”), and unobstructed reversed Rac1^f/f^ and AQP2:Rac1^f/f^ kidneys. Ki-67 signal inside AQP2^+^ CDs was filtered and converted to white dots. Scale bars, 100 μm. (**B**) Quantification of (A) showing Ki-67–positive cells per 100-μm-long CD segment with each dot representing individual mice. *n* = 4 mice per group. (**C**) Apoptosis (TUNEL; white) labeling of CDs (AQP2^+^; magenta) of paraffin kidney sections in reversed Rac1^f/f^ and AQP2:Rac1^f/f^ mice. (**D**) Quantification of TUNEL-positive cells in CDs per medullary HPF with each dot representing individual samples. *n* = 4 mice per group. (**E**) Representative flow cytometry plots of AQP2^+^ CD cells of reversed Rac1^f/f^ and AQP2:Rac1^f/f^ kidneys showing side scatter (SSC-A) against DNA [4′,6-diamidino-2-phenylindole (DAPI)] with a G_2_-M phase–specific cell cycle gate and corresponding subpopulation percentage shown in the plot. (**F**) Quantification of G_2_-M phase–specific cell populations as shown in the gating in (E) with four mice (dots) per group; bars are means ± SD. (**G**) Mitotic (pH3-positive; green) metaphase cells (condensed, aligned DNA) of repairing (reversed) CDs (AQP2; magenta) of Rac1^f/f^ and AQP2:Rac1^f/f^ mice were analyzed using 3D super-resolution confocal imaging. The far left column shows orthogonal *Z*-slices (scale bars, 2.5 μm; yellow continuous line outlines the apical lumen) as indicated by the yellow dashed line in the cross section in the middle column (scale bars, 5 μm). The far right column depicts insets as outlined by a red continuous box (scale bars, 2.5 μm). (**H**) Relative distribution of metaphase abnormalities based on a morphological assessment as shown in (G). At least 10 mitoses were analyzed per group. *P < 0.05.

In addition to morphological reconstitution, epithelial CD repair requires proliferation (*3*). We therefore investigated whether this aspect of CD repair in AQP2:Rac1^f/f^ kidneys was abnormal. We observed decreased CD cellularity in the dysmorphic AQP2:Rac1^f/f^ kidneys, when compared to the Rac1^f/f^ kidneys, suggesting a proliferation defect (fig. S8). Thus, we labeled full medullary slices with AQP2 and Ki-67 (which marks all active cell cycle phases) and performed 3D high-resolution multiplex imaging of optically cleared tissue slices to determine Ki-67–positive cells inside CDs (AQP2^+^). We performed surface reconstruction, masking, and spot-to-surface thresholding algorithms (*28*) to highlight proliferating CD cells (white dots). We found that ureteral obstruction induces a proliferative response in both groups, but AQP2:Rac1^f/f^ CDs were unable to maintain the proliferative state and showed markedly reduced Ki-67 inside AQP2^+^ tubules during repair in comparison to controls (Fig. 3, A and B, and fig. S9). In addition to the decreased proliferation, there was also increased apoptosis in repairing AQP2:Rac1^f/f^ CDs compared to controls (Fig. 3, C and D). Thus, the repair phase of AQP2:Rac1^f/f^ CDs is characterized by both cell death and hypoproliferation compared to Rac1^f/f^ CDs.

Rac1 is required for the G_1_-S phase transition in proliferating cancer cells (*20*, *29*), so we investigated whether there was decreased S phase entry and DNA synthesis in Rac1-null CDs. We pulsed mice during repair with intraperitoneal 5-bromo-2′-deoxyuridine (BrdU) and examined the tissue histologically during the repair process. Unexpectedly, there was no difference in the BrdU–to–Ki-67 ratio between groups during repair (fig. S10). Since G_2_-M arrest has been implicated in failed tubular repair in the proximal tubule, we predicted that repairing AQP2:Rac1^f/f^ CD cells would enrich in G_2_-M. We therefore performed a DNA content–based cell cycle assessment in vivo in AQP2-labeled CD cells (*30*). There were less repairing AQP2:Rac1^f/f^ CD cells in G_2_-M compared to controls, suggesting that Rac1 was not required to prevent a G_2_-M arrest but instead to maintain CD cells in G_2_-M (Fig. 3, E and F). These data suggested that there was likely a defect in cell division [M phase (mitosis)] during G_2_-M, which is a known actin cytoskeleton–dependent process. We therefore evaluated cells undergoing mitosis [marked by phosphohistone H3 (pH3)] and found that there was a defect in pH3/AQP2 double-positive G_2_-M phase cells in repairing AQP2:Rac1^f/f^ CDs suggesting a problem with mitosis (fig. S11).

### Rac1 promotes normal mitotic progression

To understand the mechanism for the M phase defect in repairing AQP2:Rac1^f/f^ CD cells, we performed high-resolution 3D confocal imaging of the mitotic substages in vivo during repair. During metaphase, Rac1^f/f^ CD cells enlarged, their condensed chromosomes (marked by pH3) aligned, and the dividing cells stayed within the plane of the epithelium (Fig. 3G). By contrast, in AQP2:Rac1^f/f^ CD cells, the condensed chromosomes appeared dysmorphic, and there was an increased rate of mitotic extrusion and more luminal mitotic cells (Fig. 3, G and H, and fig. S12). These data suggest that Rac1 is required for normal metaphase morphology.

**Fig. 4.**
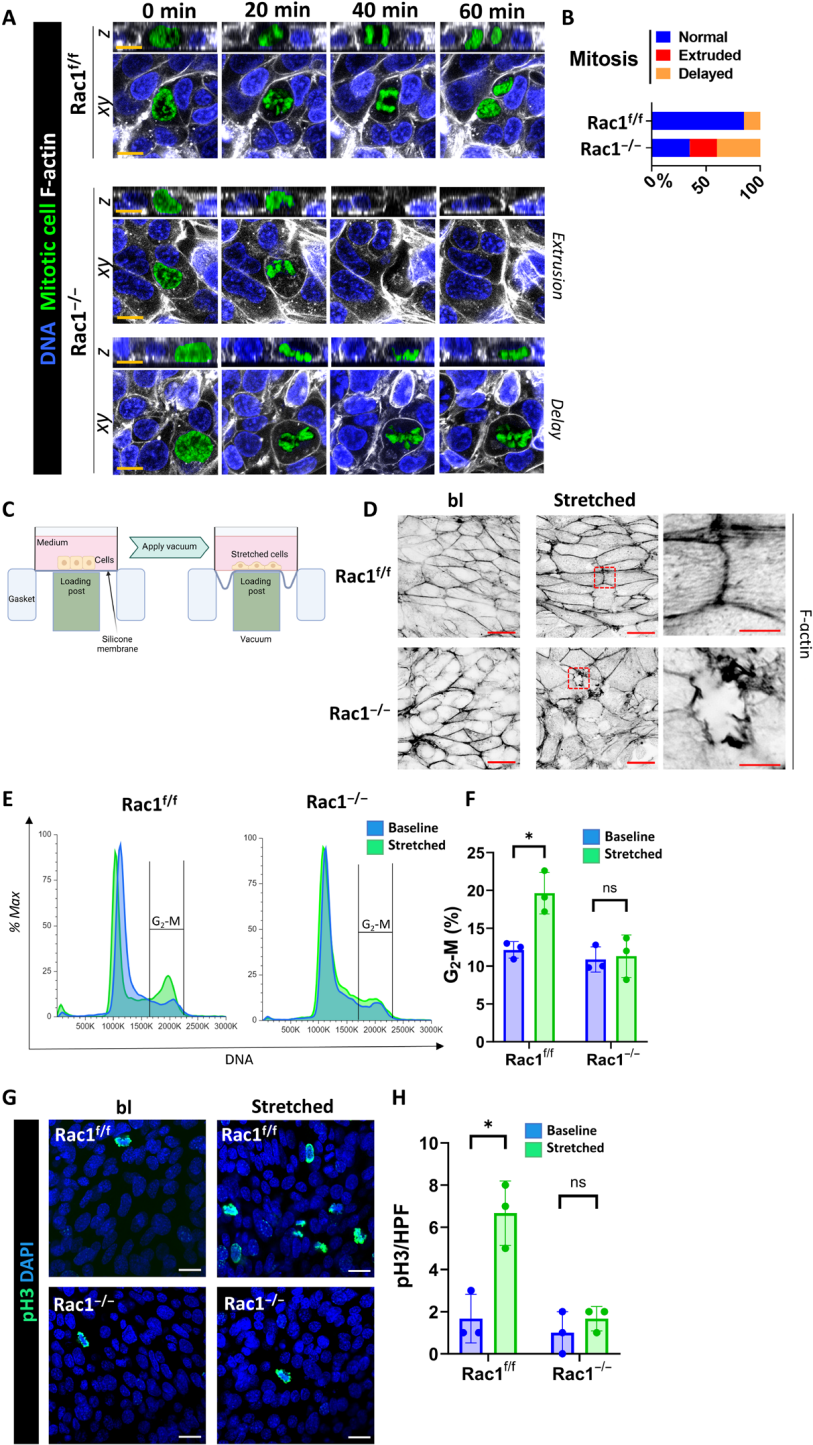
Rac1 promotes mitotic progression, F-actin integrity, and a G_2_-M cell cycle shift after CD cell injury in vitro. (**A**) Live confocal mitosis imaging of SPY650-DNA (blue)– and SPY555-actin (white)–labeled CD cells in vitro. Orthogonal *Z*-slices are also shown. Representative mitotic defects are shown for Rac1^−/−^ CD cells. Scale bars, 10 μm. The mitotic cell was manually recolored in green. (**B**) Relative distribution of mitotic defects in vitro during live imaging cell division sequences with at least 10 mitoses analyzed per group. (**C**) Schematic presentation of the Flexcell bioreactor. Created with Biorender.com (**D**) Super-resolution confocal images of apical cross sections of phalloidin-647 (F-actin)–labeled Rac1^f/f^ and Rac1^−/−^ CD cell monolayers before stretch (baseline) and after stretch (“stretched”). Images are shown as color-inverted grayscale images and are representative of three repeat experiments (scale bars, 20 μm). The red dashed box indicates the region of the inset shown in the rightmost column (scale bars, 5 μm). (**E**) Representative flow cytometry cell cycle histograms of propidium iodide (“DNA”)–labeled CD cells in vitro before stretch (baseline; blue) and after stretch (stretched, green). Plots are normalized to mode (% max), and the indicated range highlights the G_2_-M population. (**F**) Quantification of the relative G_2_-M population with bars as means ± SD and each dot representing individual samples from three independent repeat experiments. (**G**) Baseline and poststretch (stretched) Rac1^f/f^ and Rac1^−/−^ CD cell monolayers were stained for mitotic cells (pH3; green) and nuclei (DNA; blue). Images are representative of three experiments. Scale bars, 20 μm. (**H**) Quantification of mitotic cells (pH3-positive) per HPF of Rac1^f/f^ and Rac1^−/−^ CD cells before stretch (baseline; blue) and after stretch (stretched; green). Individual dots represent repeat experiments, and bars are means ± SD. *P < 0.05.

Mitosis is the shortest cell cycle phase and occurs on a scale of minutes, which makes it difficult to study (*31*). We therefore performed 3D confocal live imaging of mitosis in proliferating postscratch Rac1^f/f^ and Rac1^−/−^ CD cell monolayers grown on Matrigel-coated glass bottom dishes using SPY probes. In this model of stretch-induced mitosis, cells initially stretch over the wound area and then undergo waves of cell division in the scratch-adjacent areas (*32*). When the chromosomes condensed in the interphase to prometaphase, transition control cells rounded up, pushed against their neighbors, and successfully underwent chromosome separation (anaphase and telophase) and cytokinesis within the plane of the regenerating epithelial cell layer (Fig. 4, A and B). By contrast, Rac1^−/−^ CD cells briefly rounded up in prometaphase but then failed to maintain their morphology resulting in mitotic delay or mechanical mitotic cell extrusion (Fig. 4, A and B, and movie S4). As expected, epithelial F-actin organization was disrupted at the migrating wound edge margins of Rac1^−/−^ CD cell layers, consistent with the role of this small GTPase in promoting F-actin organization in repairing CD cells (fig. S13, A and B). There were also less mitotic cells in the scratch-adjacent areas of repairing Rac1^−/−^ CD cell layers (fig. S13, C and D). To further verify this observation in another model where cells are stretched to mimic ureteral obstruction (*33*), we grew cells on coated silicone membranes and exposed them to noncyclical uniaxial stretch (10%, 3.5 hours) in a Flexcell bioreactor (Fig. 4C). Similar to the scratch assay, poststretch Rac1^−/−^ CD cell layers demonstrated increased F-actin disruption (Fig. 4D), fewer cells in G_2_-M (Fig. 4, E and F), and less mitosis (Fig. 4, G and H). These data suggest that during repair from mechanical injury Rac1 not only maintains F-actin organization but also promotes normal cell cycling through G_2_-M by maintaining mitotic progression.

### Rac1 regulates F-actin–dependent mitotic rounding during repair

**Fig. 5.**
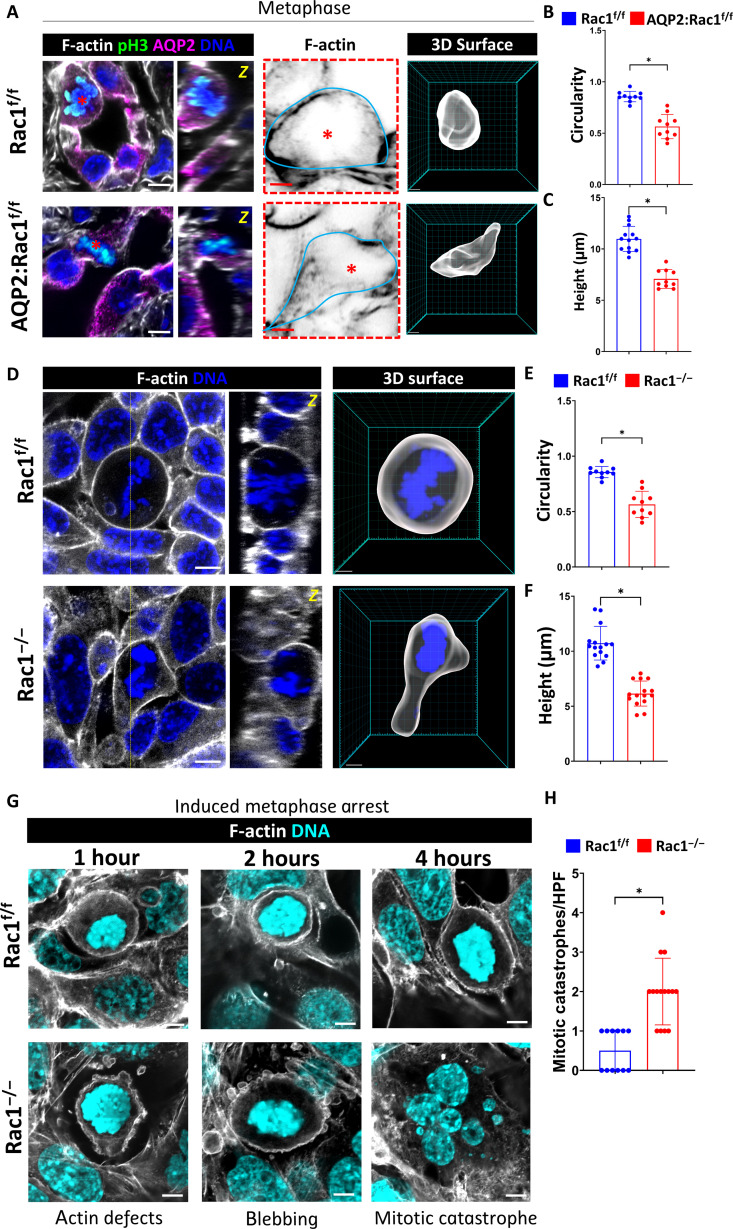
Rac1 regulates normal mitotic metaphase rounding. (**A**) Cross sections of F-actin–labeled (white) thick frozen sections. CDs (AQP2; magenta) and mitotic metaphase cells (pH3; green) of reversed/repairing mice (scale bars, 5 μm). Orthogonal *Z*-slices of the mitotic cells are shown next to the cross section as indicated by the yellow (“*z*”). The red dashed box indicates color-inverted mitotic metaphase F-actin outlined by a continuous cyan line (scale bars, 3 μm). The rightmost panel shows 3D reconstructions of the metaphase F-actin (scale bars, 3 μm). Images are representative of at least 10 mitotic figures per group pooled from at least three mice per group. (**B** and **C**) Quantification of metaphase circularity (4π × area/perimeter^2^) and height (in micrometers) showing a minimum of 10 measurements per group. Bars are means ± SD. (**D**) In vitro confocal imaging of F-actin (white)– and DNA (blue)–labeled mitotic metaphase CD cells (scale bars, 5 μm). Orthogonal *Z*-views are shown next to the cross section with the *Z*-level indicated by a yellow dashed line. 3D metaphase F-actin surface reconstructions are shown (scale bars, 3 μm). (**E** and **F**) Quantification of metaphase circularity and height (in micrometers) of control and Rac1^−/−^ CD cells showing a minimum of 10 measurements per group over at least three experiments. Bars are means ± SD. (**G**) F-actin (white)– and DNA (cyan)–labeled metaphase-arrested [using nocodazole (100 ng/ml), condensed chromosomes] Rac1^f/f^ and Rac1^−/−^ CD cells analyzed by confocal microscopy over time as indicated. Metaphase defects of Rac1^−/−^ CD cells are shown including F-actin disorganization, surface blebbing, and nuclear fragmentation with micronuclei formation indicative of mitotic catastrophes (scale bars, 5 μm). Images are representative of at least two experiments. (**H**) Quantification of mitotic catastrophes per HPF of nocodazole-arrested control and Rac1^−/−^ CD cells showing a minimum of 12 measurements per group. Bars are means ± SD. *P < 0.05.

To investigate the underlying cause for the mitotic defects, we initially imaged the microtubular spindle apparatus in the different mitotic substages but found no gross differences between Rac1^f/f^ and Rac1^−/−^ CD cells (fig. S14). We next investigated whether Rac1 deficiency affected the process of cytokinesis, which is the physical separation of daughter cells at the end of mitosis. Failure of cytokinesis typically results in aneuploidy (*34*–*36*). The aneuploidy rates were low and not increased in repairing AQP2:Rac1^f/f^ CD cells in vivo compared to Rac1^f/f^ CDs (fig. S15). We next defined whether AQP2:Rac1^f/f^ CD cells were unable to undergo mitosis due to problems with metaphase cell rounding, which is an essential F-actin–dependent process that creates the space and geometry for spindle formation and chromosome separation in epithelial cells and is closely linked to normal cell cycle progression through G_2_-M (*37*–*40*). To do this, we performed deep 3D super-resolution confocal imaging of optically cleared medullary slices after reversal of ureteral obstruction, followed by 3D reconstruction of the mitotic metaphase F-actin surface. Rac1^f/f^ CDs rounded up their metaphase F-actin surface and assumed a near-spherical shape within the otherwise cuboidal epithelium, whereas AQP2:Rac1^f/f^ CDs displayed decreased circularity and increased flattening (Figure 5, A to C). Next, we analyzed mitotic F-actin rounding in vitro by performing 3D super-resolution imaging of the F-actin cytoskeleton of mitotic metaphase CD cells in postwound closure epithelial layers. The rounded metaphase morphology of Rac1^−/−^ CD cells was severely altered with an increased axial ratio and decreased circularity compared to controls (Fig. 5, D to F). A similar rounding defect was observed when cells proliferated after mechanical stretch (fig. S16). To exclude that the rounding defect was an artifact of our 2D culture system, we grew CD cells as 3D spheroids in Matrigel, after which we performed 3D high-resolution imaging of mitotic F-actin morphology. This also showed misplaced and dysmorphic mitotic metaphase F-actin in Rac1^−/−^ CD cells (fig. S17). To test whether the F-actin–dependent rounding defect was sufficient to cause mitotic instability in metaphase, we treated postwound closure CD cell layers with low-dose nocodazole, which activates the spindle assembly checkpoint and arrests and maintains mitotic cells in the rounded metaphase stage without affecting F-actin (*39*, *41*, *42*). We monitored the mitotic state over time by super-resolution confocal imaging of F-actin and by immunoblotting for pH3. Compared to metaphase-arrested controls, which remained stably rounded over time, the Rac1^−/−^ CD cells demonstrated a disrupted metaphase F-actin structure and increased cortical blebbing and mitotic catastrophes (mitotic cell death) (Fig. 5, G and H). Furthermore, immunoblotting for the mitotic marker pH3 revealed that metaphase-arrested Rac1^−/−^ CD cells were unable to maintain the mitotic state as indicated by a decrease in pH3 that did not occur in controls (fig. S18). Thus, Rac1 is required for epithelial mitotic metaphase stability by promoting F-actin–dependent mitotic rounding.

### Rac1 promotes mitotic rounding by regulating actomyosin

**Fig. 6.**
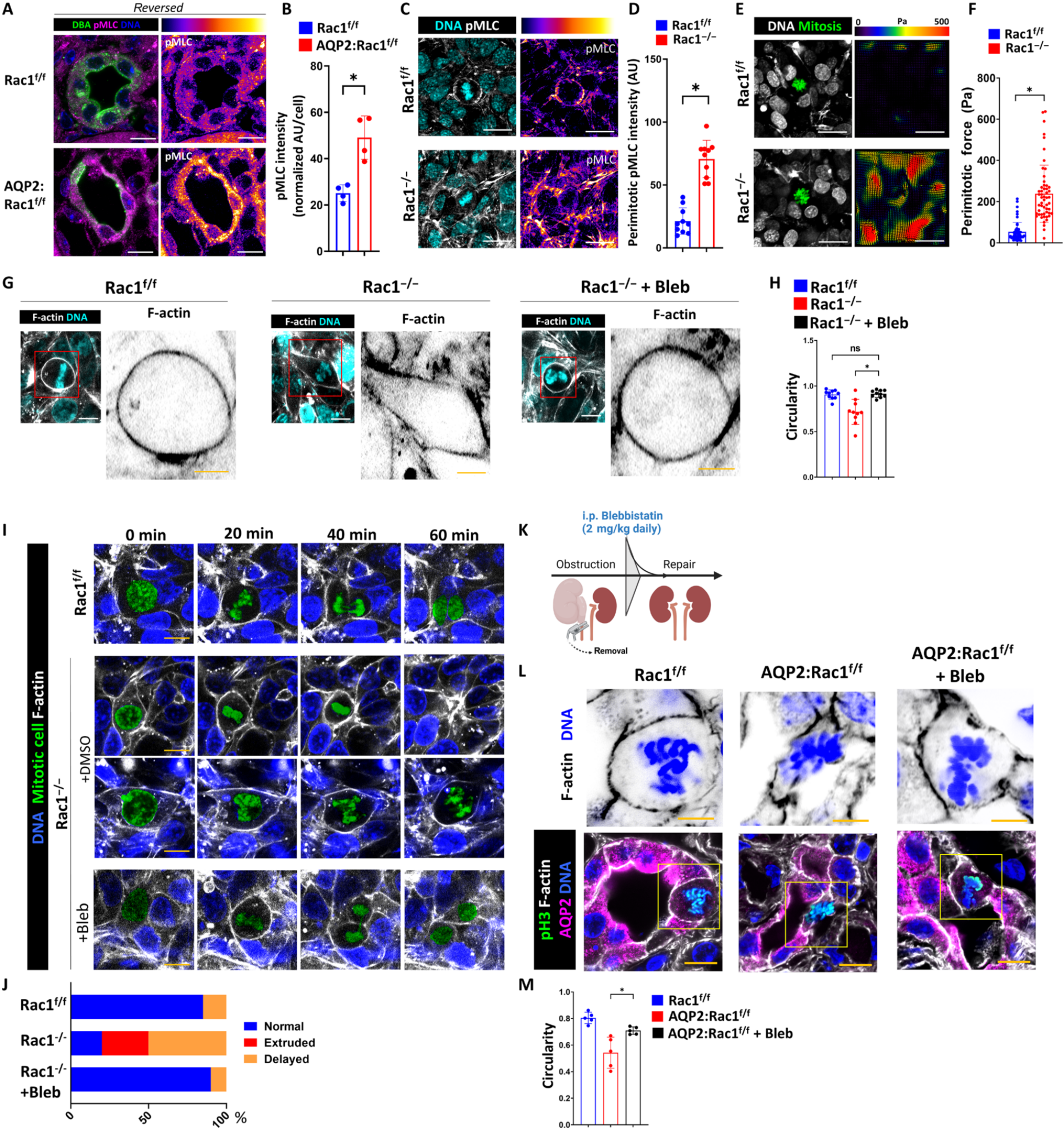
Rac1 promotes mitotic rounding by regulating actomyosin. (**A**) Representative images of activated actomyosin (pMLC) in reversed mice. CDs are labeled with DBA (dolichus biflorus agglutinin; green). pMLC is shown with a fire color scheme applied. (**B**) Quantification of average pMLC intensity with dots representing four mice per group. (**C**) pMLC (white)– and DNA (cyan)–labeled CD cell monolayers with a metaphase shown in the center. A pMLC fire color conversion is shown. Three repeat experiments. Scale bars, 20 μm (A and C). (**D**) pMLC intensity in the perimitotic area with dots representing 10 individual measurements. (**E**) F-actin (white)– and DNA (cyan)–labeled CD cells grown on a Matrigel-coated polydimethylsiloxane (PDMS) substrate with fluorescent nanobeads. Mitotic cell colored in green. Traction force maps are shown (in pascals). Scale bars, 20 μm. (**F**) Quantification of perimitotic forces. (**G**) F-actin (white)– and DNA (cyan)–labeled mitotic metaphase CD cells. Bleb, blebbistatin (5 μM). Scale bars, 10 μm. Red box outlines Metaphase F-actin shape shown on the right (scale bar, 5 μm). (**H**) Circularity quantification of mitotic metaphase F-actin as shown in (G). A minimum of 10 measurements are shown per group. (**I**) Live confocal mitosis imaging of CD cells in vitro. Bleb, 5 μM. Scale bars, 10 μm. The mitotic cell was colored in green. (**J**) Relative distribution of mitotic defects in vitro. At least 10 mitoses per group. In vitro experiments are representative of *n* = 3. (**K**) In vivo injection protocol of blebbistatin [5 days, 2 mg/kg per day, intraperitoneally (i.p.)]. Created with Biorender.com. (**L**) Super-resolution confocal imaging of mitotic CD cells during repair (scale bars, 10 μm). The yellow box outlines the insets that are shown in the top row, which depict color-inverted metaphase F-actin (scale bars, 5 μm). (**M**) Quantification of mitotic metaphase circularity from (L) showing *n* = 4 mice per group. Bars are means ± SD. *P < 0.05.

Epithelial actin cytoskeletal networks generate force and contractility by incorporating the motor protein myosin that determines the mechanical properties of an epithelium (*43*). We previously showed that Rac1-depedent actin cytoskeletal organization is required to restrict excessive actomyosin activity in CD cells by maintaining actin branching (*18*). We therefore determined whether the abnormality in mitotic morphology was a mechanical defect due to actomyosin dysregulation. We confirmed that there is increased activated actomyosin in repairing AQP2:Rac1^f/f^ CDs compared to controls by immunostaining for phospho–myosin light chain (pMLC) (Fig. 6, A and B). We next investigated whether actomyosin was dysregulated around dysmorphic mitotic metaphase cells in regenerating Rac1^−/−^ CD monolayers in vitro. While Rac1^f/f^ CD cells showed some enrichment of pMLC in the rounded mitotic actin cortex, this was not observed in mitotic Rac1^−/−^ CD cells. Instead, strong, and diffuse actomyosin activation was seen in the Rac1^−/−^ CD cells that surround mitotic cells (Fig. 6, C and D). To determine whether the dysregulation of actomyosin translates into increased mechanical tension around mitotic cells, we performed traction force microscopy and found that Rac1^−/−^ CD cell layers displayed increased mechanical force in the area adjacent to mitotic prometaphase cells when compared to Rac1^f/f^ CD cells, suggesting that actomyosin dysregulation leads to a force imbalance and increased tension around mitotic cells (Fig. 6, E and F). These data suggest that Rac1 restricts excessive actomyosin force generation, which is likely required to maintain normal mitotic morphology. Direct myosin inhibition has been shown to decrease the mechanical force generation by epithelial cells (*44*). Hence, we hypothesized that direct myosin inhibition would reverse the Rac1^−/−^ phenotype of failed rounding and cell division. We therefore inhibited the mechanical force on mitotic cells by treating Rac1^−/−^ CD cell layers in vitro with low doses of the direct myosin inhibitor blebbistatin. This treatment reversed the F-actin mitotic rounding defect in Rac1^−/−^ CD cells, suggesting that Rac1 promotes normal mitotic F-actin rounding by modulating actomyosin (Fig. 6, G and H). Similarly, blebbistatin reversed the F-actin rounding defect in Rac1^−/−^ CD spheroids in a 3D matrix (fig. S19). We then assessed whether excess actomyosin activity inhibition was sufficient to rescue the defect in mitotic progression of dividing Rac1^−/−^ CD cells by treating monolayers with low-dose blebbistatin and performing live confocal mitosis imaging. Blebbistatin reversed the mitotic extrusion and metaphase delay defects in dividing Rac1^−/−^ CD cells (Fig. 6, I and J, and movie S5). To assess whether this finding could be recapitulated in vivo, we administered blebbistatin to mice upon clip removal and reversal of obstruction *(*Fig. 6K). Five days after daily blebbistatin injection, we performed in vivo super-resolution confocal microscopy of the metaphase actin cytoskeleton of fresh frozen medullary slices and found that myosin-inhibited mitotic AQP2:Rac1^f/f^ CD cells have a markedly improved morphology with increased size and circularity, whereas vehicle-treated mitotic mutant CD cells remained flat and dysmorphic with decreased rounding (Fig. 6, L and M). Together, these data indicate that Rac1 promotes actin-dependent mitotic metaphase rounding by controlling actomyosin, which is required to maintain mitotic stability.

### Direct actomyosin inhibition is sufficient to reverse the repair defect of AQP2:Rac1^f/f^ kidneys

**Fig. 7.**
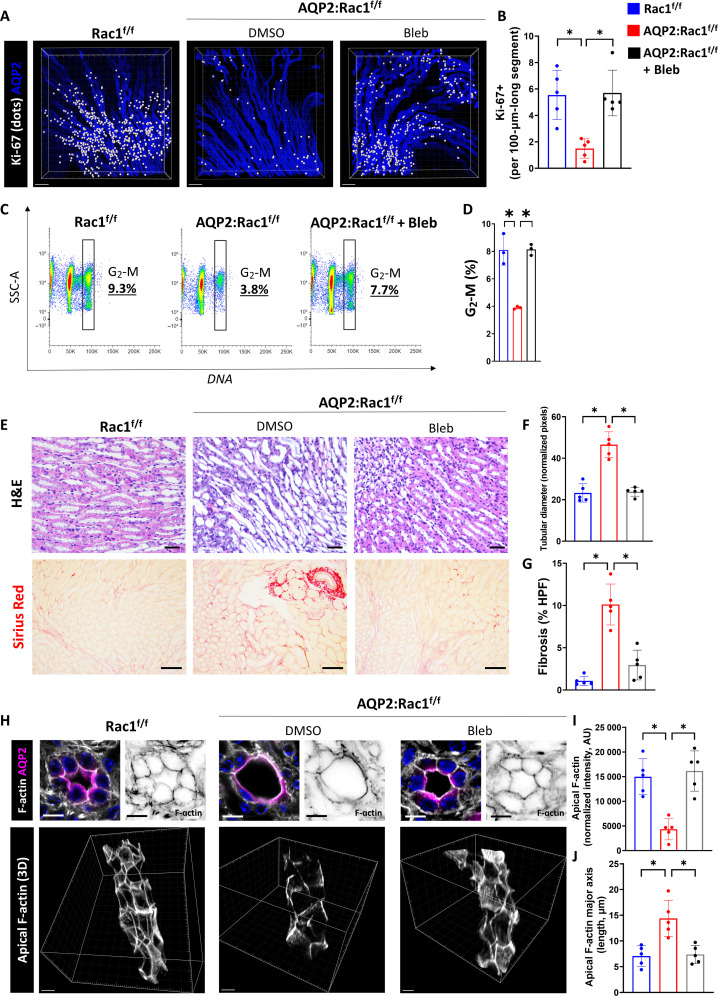
Actomyosin inhibition reversed the repair defect of AQP2:Rac1^−/−^ mice. (**A**) Optically cleared medullary slices of reversed (repairing) mice labeled with AQP2 (CDs) and Ki-67. 3D reconstructions are shown. Ki-67 signal inside AQP2^+^ CDs was filtered and converted to white dots. Scale bars, 100 μm. (**B**) Quantification of (A) showing Ki-67–positive cells per 100-μm-long CD segment with dots representing individual mice (*n* = 5). (**C**) Flow cytometry plots of AQP2^+^ CD cells of reversed kidneys showing side scatter (SSC-A) against DNA (DAPI) with a G_2_-M phase–specific cell cycle gate. Subpopulation percentages are shown in the plot. (**D**) Quantification of G_2_-M CD cells with at least three mice (one dot) per group. (**E**) H&E paraffin kidney sections in the first row (scale bars, 20 μm) of medullary regions and Sirius Red staining in the second row (scale bars, 50 μm). *n* = 5 mice per group. (**F** and **G**) Quantification of medullary tubular diameter as normalized pixels and fibrosis (as percentage of Sirius Red–positive area per HPF) with dots representing individual mice. (**H**) F-actin labeling (white) of CDs (AQP2; magenta) in thick fresh frozen medullary kidney slices in reversed kidneys analyzed by 3D super-resolution confocal. First row depicts two cross sections (left original, right F-actin channel; scale bars, 10 μm), and second row shows oblique views of 3D reconstructions of the apical CD F-actin (scale bars, 7 μm). (**I** and **J**) Quantification of apical F-actin intensity and apical F-actin major axis length; dots represent individual mice. *n* = 5 mice per group. Bars are means ± SD. *P < 0.05.

Since blebbistatin rescued the mitotic rounding defects, we determined its effects on CD cell proliferation and repair following obstruction. To do this, we labeled full medullary slices collected after the obstruction was reversed with AQP2 and Ki-67. We then performed confocal high-resolution multiplex imaging and postimaging surface reconstructions applying spot-to-surface thresholding algorithms. We found that blebbistatin treatment rescued the proliferation defect and decreased apoptosis of repairing AQP2:Rac1^f/f^ kidneys with similar proliferation rates to untreated controls (Fig. 7 A and B, and figs. S20 and S21). We confirmed that blebbistatin does not induce CD cell proliferation in uninjured control kidneys at baseline (fig. S22). As blebbistatin administration during obstructive injury has been shown to affect macrophage motility (*45*), we counted immunolabeled total macrophages (CD68^+^) around untreated and blebbistatin-treated repairing AQP2:Rac1^f/f^ CDs. There was no major difference, suggesting that modulation of immune cell infiltration is not a confounding mechanism whereby blebbistatin rescues the repair defect in the absence of Rac1 (fig. S23). When we performed DNA content–based cell cycle assessment by flow cytometry of dissociated kidneys, we noted that actomyosin inhibition also rescued the G_2_-M cell cycle defect of repairing AQP2:Rac1^f/f^ CDs (Fig. 7, C and D). We further showed that blebbistatin ameliorated medullary tubular dilatation and fibrosis to similar levels as untreated controls (Fig. 7, E to G). Last, we performed thick frozen sectioning of fresh tissue, followed by high-resolution 3D confocal imaging and reconstruction of the actin cytoskeleton to demonstrate full restoration of apical F-actin density and morphology of the apical actin cytoskeleton of actomyosin-inhibited AQP2:Rac1^f/f^ CDs when compared to the vehicle-treated AQP2:Rac1^f/f^ CDs (Fig. 7, H to J, and movie S6). These data indicate that Rac1-dependent modulation of actomyosin is required to promote normal proliferation and cell cycling through G_2_-M during repair, which are essential for epithelial CD repair.

### Rac1 restricts myosin activation by inhibiting the RhoA activator guanine nucleotide exchange factor–H1

**Fig. 8.**
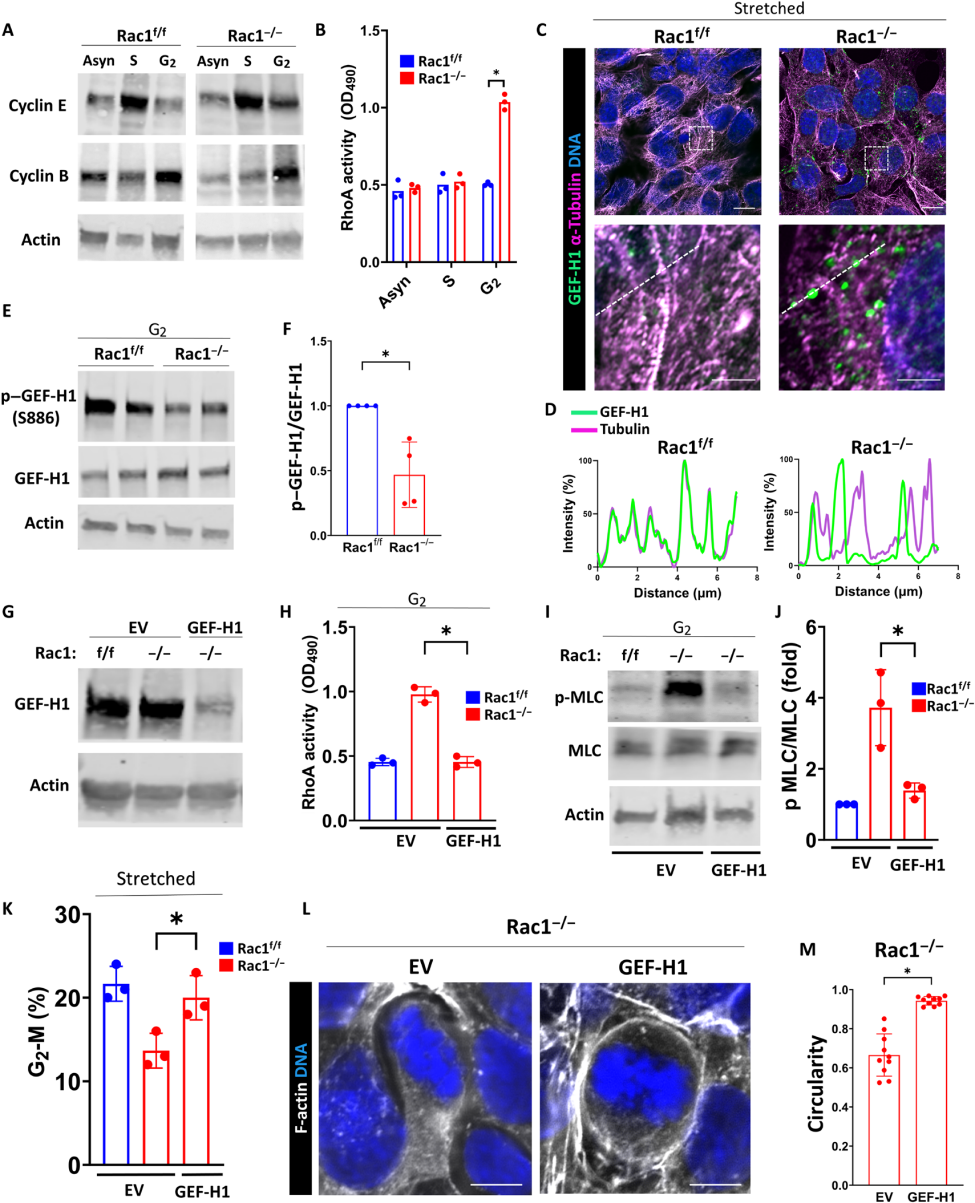
Rac1 restricts myosin activation by inhibiting the RhoA activator GEF-H1. (**A**) Immunoblots of asynchronous (Asyn), G_1_-S–synchronized, or S phase–synchronized CD cells to confirm cell cycle phase–specific enrichment (cyclin E, S phase; cyclin B1, G_2_ phase). OD_490_, optical density at 490 nm. (**B**) Quantification of RhoA activity (asynchronous, S, and G_2_) using G-LISA and measuring absorbance at 490 nm. Individual dots represent repeat experiments (*n* = 3). (**C**) Super-resolution confocal imaging of microtubules (magenta) and GEF-H1 (green) of CD cells after stretch. Scale bars, 10 μm (first row) and 2.5 μm (second row). The white dashed box indicates the region of interest for the insets in the second row. (**D**) Line scan profile (white dashed line) normalized to the highest (100%) and lowest (0%) intensity. Representative of three repeat experiments and 10 measurements per group. (**E** and **F**) Immunoblotting of G_2_-synchronized cell lysates for phosphorylated (serine-886, S886) and total GEF-H1 in biological duplicates. Three repeat experiments were quantified in (F). Fold change values ± SD. (**G**) Immunoblotting of empty vector (EV)–transfected or GEF-H1 knockdown short hairpin RNA (shRNA)–transfected CD cells. (**H**) Quantification of RhoA activity in G_2_ cell cycle fractions. Individual dots represent repeat experiments (*n* = 3). (**I** and **J**) Immunoblotting of G_2_ cell cycle fractions of EV or GEF-H1 knockdown transfected for total and pMLC (serine-19). The phospho-to-total myosin light chain (pMLC–to–t-MLC) ratio is shown as fold change ± SD with individual dots representing repeat experiments (*n* = 3). (**K**) Quantification of G_2_-M cell cycle fractions (in percentage) of the indicated groups after induction of proliferation using stretch by flow cytometry using propidium iodide. Individual dots represent experiments (*n* = 3). (**L** and **M**) F-actin (white)– and DNA (blue)–labeled mitotic metaphase EV- or GEF-H1 knockdown–transfected Rac1^−/−^ CD cells (scale bars, 5 μm). Mitotic rounding (circularity) is quantified in (M) showing a minimum of 10 measurements per group. Bars are means ± SD. *P < 0.05.

Next, we sought to determine the mechanism whereby Rac1 suppressed actomyosin activity. Classically, Rac1 regulates myosin activity via its mutual antagonism with the other canonical Rho-GTPase RhoA (*46*, *47*). Although we previously showed that RhoA activity is not changed in asynchronous (non–cell cycle–synchronized) Rac1^−/−^ CD cells (*18*), it is possible that interactions between Rac1 and RhoA occur in a cell cycle phase–specific manner in kidney CD cells. To investigate this, we first synchronized cells at the beginning of S phase using a double thymidine block (enriched for cyclin E) or at the beginning of G_2_ using the cyclin-dependent kinase 1 (Cdk1) inhibitor RO-3306 (enriched for cyclin B1) (Fig. 8A) (*48*–*50*). We then measured RhoA activity using G-LISA and found that Rac1^−/−^ CD cells have abnormally increased RhoA activity specifically at the beginning of G_2_ (Fig. 8B). The antagonistic cross-talk between Rac1 and RhoA is mainly mediated by upstream regulatory guanine nucleotide exchange factors (GEFs) (*47*). The microtubule-associated GEF-H1 (also known as Arhgef2) is a RhoA-activating GEF that mediates the cell cycle phase–specific cross-talk between microtubules and the actin cytoskeleton, and its activity is regulated through a cycle of microtubule binding and release (*51*–*55*). This cycle is controlled by Rac1-dependent serine-886 phosphorylation of GEF-H1 via its effector p21-Activated kinase 1 (Pak1) that promotes the microtubular translocation and inhibition of GEF-H1 (*56*). GEF-H1 was recently shown to be released upon interphase microtubular disassembly promoting mitotic entry and controlling mitotic cell shape (*57*). On this basis, we hypothesized that GEF-H1 controls the antagonistic cell cycle phase–specific Rac1-RhoA interaction. Super-resolution imaging of microtubules in uniaxially stretched G_2_-shifted CD cells revealed increased free (not associated with microtubules) GEF-H1 in Rac1^−/−^ CD cells indicating increased GEF-H1 activity toward RhoA (Fig. 8, C and D). Consistent with this, the inhibitory S886 phosphorylation of GEF-H1 was significantly decreased in Rac1^−/−^ CD cells (Fig. 8, E and F). GEF-H1 knockdown in Rac1^−/−^ CD cells abolished the abnormally elevated RhoA activity and myosin phosphorylation in G_2_ (Fig. 8, G to J). Last, GEF-H1 knockdown in Rac1^−/−^ CD cells was sufficient to rescue the cell stretch–triggered G_2_-M phase cell cycle shift and restore mitotic rounding (Fig. 8, K to M). These data suggest that Rac1 suppresses abnormal actomyosin activation in the G_2_ cell cycle phase through RhoA antagonism by promoting the phosphorylation, inhibition, and microtubular association of the RhoA-activating GEF-H1. As GEF-H1 activates RhoA that regulates actomyosin via Rho-associated coiled-coil containing protein kinases (ROCK1/2) (*58*), we tested whether pharmacological inhibition of ROCK1/2 would rescue the mitotic rounding and epithelial repair defect. Similar to direct myosin inhibition with blebbistatin, ROCK inhibition with Y-27632 rescued the defects in mitotic morphology and promoted mitotic rounding in Rac1^−/−^ CD cells (fig. S24). Next, we injected AQP2:Rac1^f/f^ mice with Y-27632 upon reversal of ureteral obstruction [10 mg/kg, intraperitoneally (i.p.), per day for 5 days]. Inhibition of ROCK in vivo in repairing AQP2:Rac1^f/f^ mice restored CD mitotic rounding, proliferation, and tissue and actin cytoskeleton morphology, mimicking blebbistatin treatment (figs. S24 and S25). These data support a mechanistic model whereby Rac1 promotes repair via suppression of the GEF-H1-RhoA-ROCK-actomyosin signaling axis that is required to maintain myosin-dependent epithelial morphology and cell cycling.

### Rac1-dependent actomyosin regulates a mechanical G_2_-M checkpoint to prevent premature mitotic entry

**Fig. 9.**
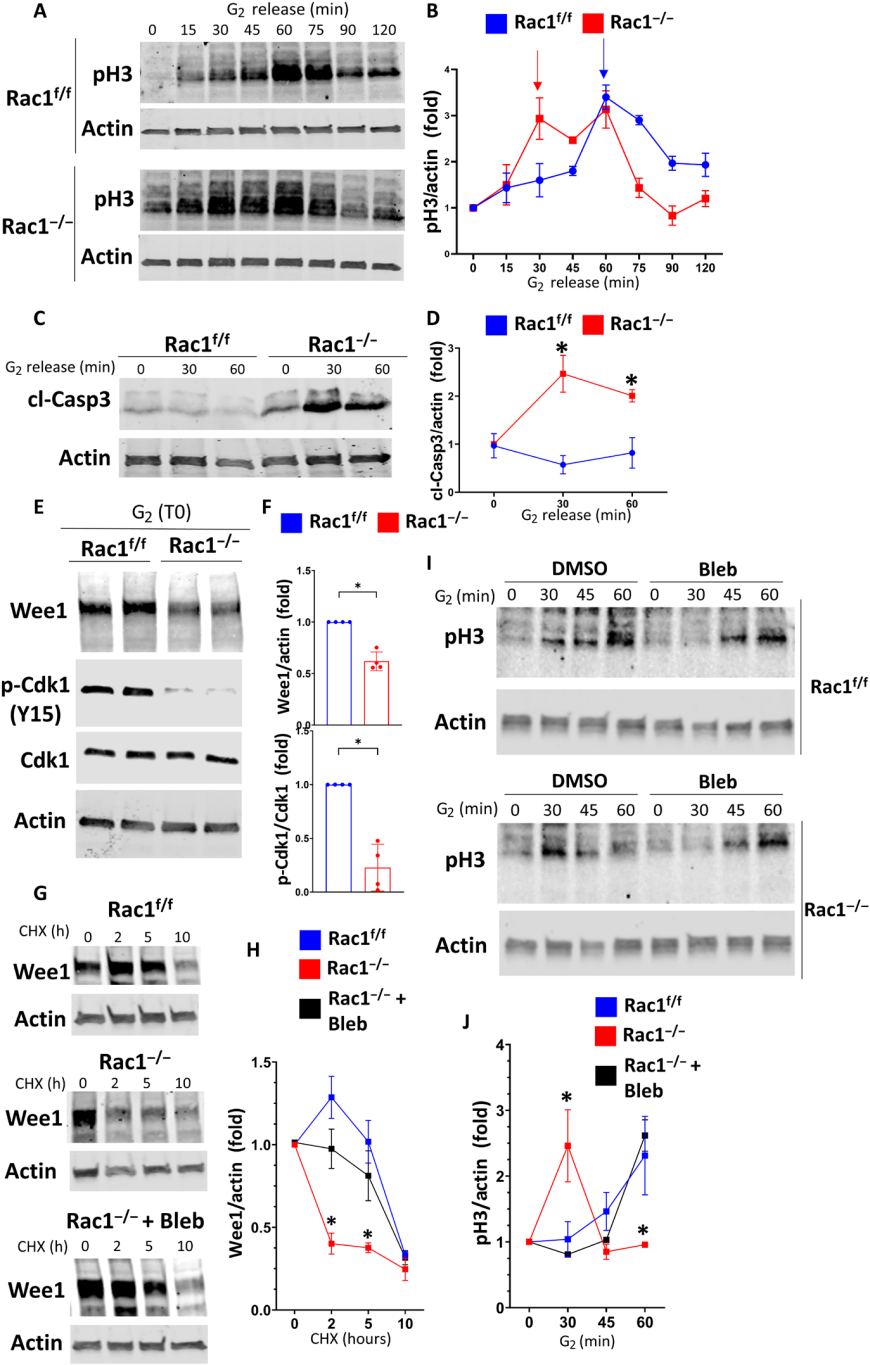
Rac1 via actomyosin regulates the G_2_-M checkpoint kinase Wee1 to prevent premature mitotic entry. (**A** and **B**) CD cells were G_2_-synchronized using RO-3306, and lysates were collected at the indicated time points after RO-3306 washout (“G_2_ release”). Lysates were immunoblotted for pH3 to monitor mitotic entry. Three repeat experiments were quantified in (B). Fold change values ± SD. Arrows highlight the first pH3 peak of the respective groups indicating mitotic entry. (**C** and **D**) G_2_-synchronized CD cells were immunoblotted for cleaved caspase 3 (cl-Casp3) after G_2_ release and quantified in (D) as fold change values ± SD (*n* = 3). Asterisk (*) denotes between-group significance at the corresponding time point. (**E** and **F**) CD cells were G_2_-synchronized, and lysates were obtained immediately upon G_2_ washout (G_2_, T0) and immunoblotted in biological duplicates. p-Cdk1 Y15: phosphorylated Cdk1 tyrosine-15. Three repeat experiments were quantified in (F). Fold change values ± SD. (**G** and **H**) Asynchronous Rac1^f/f^ (+DMSO) and Rac1^−/−^ [+DMSO or blebbistatin (5 μM)] CD cells were treated with cycloheximide (CHX; 100 μg/ml), and lysates were obtained at the indicated time points and immunoblotted for Wee1. Three repeat experiments are quantified in (H) as fold change ± SD. Asterisk (*) denotes significance between Rac1^−/−^ and Rac1^f/f^ or blebbistatin-treated Rac1^−/−^ at the corresponding time point. (**I** and **J**) Rac1^f/f^ and Rac1^−/−^ CD cells were G_2_-synchronized and treated with vehicle (DMSO) or 5 μM blebbistatin upon G_2_ release. Lysates were collected at the indicated time points and immunoblotted for pH3 to monitor mitotic entry. Three independent repeat experiments are quantified (for Rac1^f/f^, only the vehicle control is shown) in (J) as fold change values ± SD. Asterisk (*) denotes significance between Rac1^−/−^ and Rac1^f/f^ or blebbistatin-treated Rac1^−/−^ at the corresponding time point. *P < 0.05.

Our findings suggested that Rac1-dependent actomyosin regulation via RhoA antagonism is required to promote the orderly cell cycle progression through G_2_-M, specifically mitosis. Unexpectedly, however, we found that dysmorphic Rac1-deficient cells entered mitosis and did not go into cell cycle arrest, suggesting an abnormal G_2_-M checkpoint override in cells that were clearly malformed. We therefore investigated whether failed cell cycle checkpoint activation contributed to our Rac1-deficient phenotype of mitotic dysmorphology, rounding failure, extrusion, and impaired proliferation. We performed cell cycle synchronization at the G_1_-S border of control and Rac1^−/−^ CD cells in vitro using a double thymidine block and immunoblotted for cyclin B1 as a positive control as it rises steadily after thymidine washout, peaks in G_2_, and then drops sharply in mitosis (*59*). We found that while the cyclin B1 peak is preserved in Rac1^−/−^ CDs, it is notably shifted toward earlier time points (fig. S26). To test whether this is associated with earlier mitosis, we immunoblotted for the mitotic marker pH3 that confirmed abnormally early mitosis in Rac1^−/−^ CD cells (fig. S26). Next, to specifically study mitotic entry, we synchronized cells in G_2_ using the reversible Cdk1 inhibitor RO-3306 (*49*, *60*). Rac1^f/f^ CD cells entered mitosis within 60 min of RO-3306 washout, whereas Rac1^−/−^ CD cells rapidly entered mitosis within ~15 min indicating a shortened G_2_ phase and premature mitotic entry (Fig. 9, A and B). G_2_-M checkpoint override has recently been linked to mitotic cell death in cancer cells (*61*–*63*). We thus investigated whether the premature mitotic entry is related to increased mitotic instability and cell death. Rac1^−/−^ CD cells demonstrated increased cleaved caspase 3 activation upon release into G_2_ and mitotic entry indicating increased mitosis-associated cell death (Fig. 9, C and D). The orderly progression through G_2_ and mitotic entry is controlled by the G_2_-M checkpoint kinase Wee1, which phosphorylates and inhibits Cdk1 on tyrosine-15 (Y15) to suppress early mitotic events of cells unprepared for mitosis (*64*, *65*). Wee1 inhibition leads to loss of G_2_-M checkpoint inhibition and early forced mitotic entry, reminiscent of the Rac1^−/−^ phenotype (*61*–*63*). To test whether Wee1 is regulated by Rac1, we synchronized cells in G_2_ using RO-3306 and obtained lysates immediately after washout (G_2_ fraction). We found that Wee1 and Y15-Cdk1 levels were reduced in Rac1^−/−^ CD cells in G_2_ (Fig. 9, E and F). Since Wee1 levels and function are regulated by proteasomal degradation (*64*, *66*, *67*), we treated asynchronous control and Rac1^−/−^ CD cells with the protein synthesis inhibitor cycloheximide and followed Wee1 levels over time. While Wee1 levels remained relatively stable over 5 to 7 hours in Rac1^f/f^ CD cells, they dropped sharply after 1 to 2 hours in Rac1^−/−^ CD cells indicating that Rac1 is required for Wee1 protein stability (Fig. 9, G and H). As Wee1 levels are modulated by actomyosin and degraded by excessive myosin contractility (*65*), we tested whether Wee1 instability was reversible with blebbistatin treatment of the Rac^−/−^ CD cells. This was indeed the case (Fig. 9, G and H), suggesting that the effect in the Rac1-deficient CD cells was mediated by excessive actomyosin-dependent forces. Furthermore, myosin inhibition in G_2_-synchronized Rac1^−/−^ CD cells with blebbistatin or an ROCK inhibitor abrogated premature mitotic entry, suggesting that the actomyosin dependent stabilization of Wee1 downstream of Rac1 is required for normal mitotic entry (Fig. 9, I and J, and fig. S27).

### Wee1 inhibition recapitulates Rac1 deficiency

**Fig. 10.**
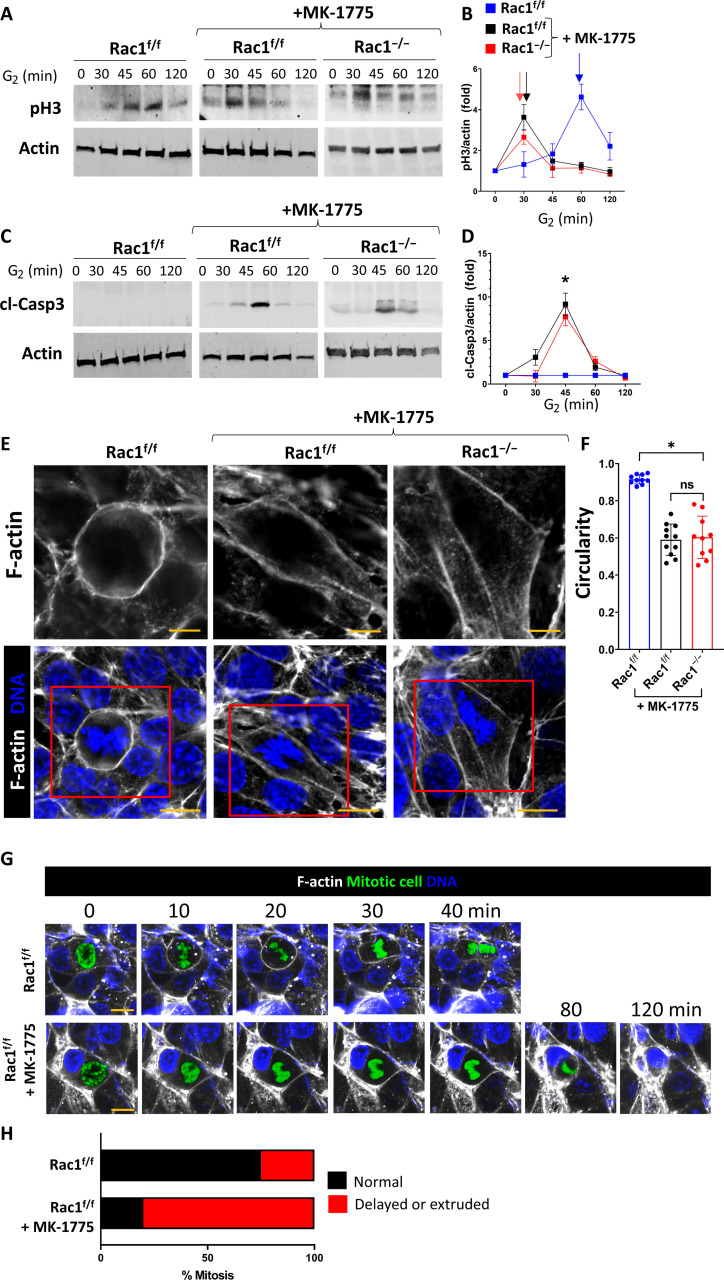
Wee1 inhibition phenocopies Rac1 deficiency in mitosis. (**A** to **D**) Rac1^f/f^ and Rac1^−/−^ CD cells were G_2_-synchronized using RO-3306 and treated with the Wee1-specific inhibitor MK-1775 (1 μM) upon G_2_ release. Lysates were collected at the indicated time points and immunoblotted for pH3 to monitor mitotic entry or cleaved caspase 3 to monitor cell death. Densitometry was used to quantify fold changes ± SD of three repeat experiments in (B) and (D). Arrows in (B) highlight the first pH3 peak indicating mitotic entry. (**E**) F-actin (white)– and DNA (blue)–labeled Rac1^f/f^ and Rac1^−/−^ (+MK-1775; 1 μM) CD cell monolayers analyzed by confocal microscopy with a mitotic metaphase cell shown in the center (scale bars, 10 μm). The top row column depicts metaphase F-actin (scale bars, 5 μm) as outlined by a red continuous box in the bottom row. Images are representative of at least three experiments. (**F**) Circularity quantification of mitotic metaphase F-actin as shown in (E) with a minimum of 10 measurements shown per group. Bars are means ± SD. (**G**) Live confocal mitosis imaging of SPY650-DNA (blue)– and SPY555-actin (white)–labeled vehicle (DMSO)– or MK-1775 (1 μM)–treated Rac1^f/f^ CD cells in vitro. The mitotic cell was manually segmented and colored in green. Scale bars, 10 μm. Sequences are representative of three repeat experiments with at least three to four mitoses analyzed per experiment. (**H**) Relative distribution of mitotic defects in vitro during live imaging cell division sequences with at least 10 mitoses analyzed per group. *P < 0.05.

To determine whether Rac1-dependent Wee1 stabilization was sufficient to promote normal mitotic entry, we treated G_2_-synchronized Rac1^f/f^ CD cells with the Wee1-specific inhibitor adavosertib (MK-1775). These cells behaved similar to the Rac1^−/−^ CD cells showing premature mitotic entry and mitosis-associated cell death indicating that Rac1-dependent regulation of Wee1 is sufficient to control mitotic entry (Fig. 10, A to D). We also verified that Wee1 inhibition resulted in abnormal actin–dependent mitotic rounding and mitotic progression in postwound closure Rac1^f/f^ CD cells or CD spheroids in a 3D matrix in vitro (Fig. 10, E to H, and fig. S28 and movie S7). Thus, Rac1-dependent stabilization of Wee1 controls mitotic entry that is required to maintain mitotic morphology and progression.

### Rac1 levels are associated with preserved CD morphology in human chronic kidney disease

**Fig. 11.**
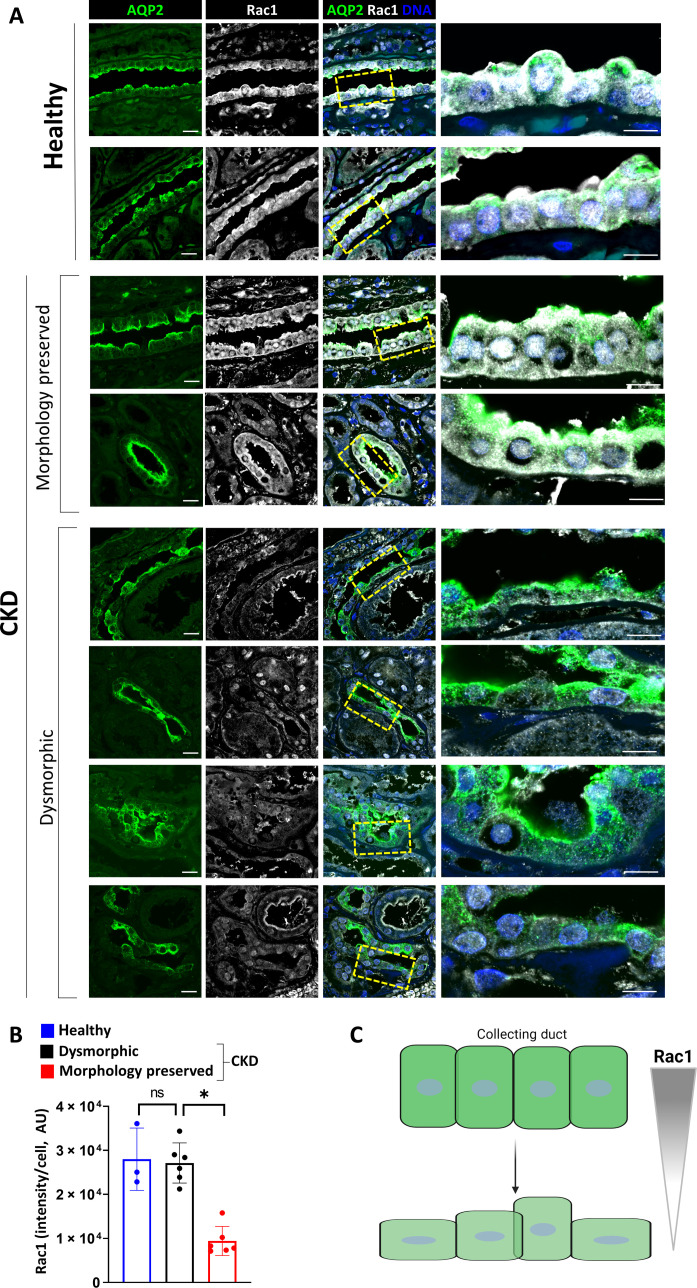
Rac1 is associated with preserved CD morphology in chronic kidney disease. (**A**) Rac1 immunostaining (white) of human kidney biopsy specimens (paraffin) with the CDs marked by AQP2 (green). Scale bars, 20 μm. Control (“healthy”) biopsy displays a typical columnar to cuboidal CD morphology. Chronic kidney disease biopsy specimens were grouped according to CD morphology (preserved versus dysmorphic). Two or 4 representative stainings from different subjects (*n* = 3 healthy controls and 6 different chronic kidney disease biopsy specimens) are shown per group. The rightmost panel shows an inset that is outlined by a dashed yellow box in the original image. Scale bars, 10 μm. (**B**) Quantification of normalized Rac1 staining intensity per cell with dots representing the intensity per sample with 35 cells per sample analyzed. Bars are means ± SD. *P < 0.05. (**C**) Schematic representation of the proposed association between Rac1 and CD morphology. Created with Biorender.com.

Our mouse model indicates that Rac1 is essential for maintaining and rebuilding the structural integrity of injured CDs. In humans, CD integrity is a major determinant of renal function, and many known causes of chronic kidney disease are associated with notable CD morphological abnormalities (*68*–*70*). Thus, we analyzed the protein level of total Rac1 in the CDs of kidney biopsies from healthy controls or individuals with chronic kidney disease with fibrosis. Rac1 is readily detected in the CDs of healthy controls (Fig. 11A), and its expression is preserved in CDs from patients with chronic kidney disease who maintain their typical cuboidal to columnar morphology (Fig. 11, A and B). By contrast, the levels of Rac1 are markedly decreased in the dysmorphic CDs of individuals with chronic kidney disease, suggesting that Rac1 expression plays a role in the preservation of CD morphology in chronic kidney disease (Fig. 11C).

### Mechanical CD cell injury results in Hace1-mediated Rac1 ubiquitination and degradation

The finding that total Rac1 levels correlate with preserved morphology in human chronic kidney disease (CKD) suggested that this is a critical event in mediating irreversible chronic injury of CDs. In addition, proteasome-mediated degradation of Rac1 was shown to contribute to loss of epithelial morphology (*71*). We therefore sought to determine the mechanism whereby total Rac1 is regulated. The Rac1-specific ubiquitin ligase was identified to be the HECT domain and ankyrin repeat containing E3 ubiquitin protein ligase 1 (HACE1), which polyubiquitinates (>50 kDa) Rac1 and efficiently targets Rac1 for degradation (*72*–*74*). Furthermore, Hace1 was recently identified as a mechanosensitive ubiquitin ligase linking extracellular matrix compliance to Rac1-driven migration and cell cycling (*75*). When we assessed the levels of Hace1 in our murine model, we found it to be up-regulated in irreversible but not in reversible ureteral obstruction. Consistent with this, Rac1 levels decrease in irreversible but not reversible injury (fig. S29). To assess the mechanistic relevance of this finding, we used a well-established in vitro cell compression model where confluent cells are covered with a 1:1 mixture of low gelling agarose and full medium creating a thin gel layer with a defined height and weight that provides low but constant pressure deforming the cell layer (fig, S30, A and B) (*76*–*79*). Long (4 hours)—but not short (1 hour)—cell compression reduced Rac1 and increased Hace1 levels (fig. S30, C and D). We then performed Rac1 immunoprecipitation, followed by Western blotting for ubiquitination at different time points after cell compression. These studies indicate that long but not short cell compression of CD cells in vitro lead to polyubiquitination and degradation of Rac1 (fig. S30E). This effect was abolished by genetic inhibition of Hace1 using lentivirus-mediated transfection of Hace1 short hairpin RNA (shRNA) into compressed control cells (fig. S30, F to H). These data suggest a model whereby chronic mechanical epithelial injury results in Hace1-mediated Rac1 degradation, which potentially hampers cellular adaption to a higher mechanical pressure environment as found in chronic human kidney disease with a profibrotic stiff extracellular matrix.

## DISCUSSION

Relieving kidney collecting system obstruction before irreversible fibrosis results in tubule repair and regeneration. This requires complex cellular processes that include reconstitution of cell morphology and proliferation. In this study, we show that Rac1-dependent regulation of the actin cytoskeleton, a key component for normal cell morphology, is required for the cell proliferation necessary for CD repair following reversal of obstructive injury. We demonstrate that an abnormal actin cytoskeleton induced by deleting Rac1 in CDs results in a dysmorphic epithelial cell that aberrantly enters mitosis and fails to maintain mitotic rounding resulting in mitotic extrusion, apoptosis, and impaired proliferation and repair. This is mediated by the inability of the abnormal actin cytoskeleton to stabilize the G_2_-M checkpoint inhibitor Wee1 (fig. S31). This finding together with our observation that decreased Rac1 expression is observed in dysmorphic CDs of patients with chronic kidney disease suggests that Rac1 mediates the morphological integrity of CD repair. Furthermore, it highlights that preventing dysmorphic cells from aberrantly entering mitosis is critical for epithelial repair.

There is little data on the role of Rac1 in kidney injury. Most studies were performed in the podocyte where Rac1 deletion induced no discernible developmental phenotype (*80*). However, selective Rac1 deletion in podocytes was shown to be protective in a model of protamine induced podocyte foot process effacement (*80*). Consistent with this finding, abnormal activation of Rac1 expressed in podocytes were also shown to induce functional alterations, maladaptive cytoskeletal remodeling, and focal segmental glomerulosclerosis (*81*–*83*). These studies are in contrast with the finding that Rac1 deletion increased podocyte loss and glomerulosclerosis in diabetic nephropathy and Adriamycin models (*84*). Thus, in the podocyte, Rac1 exerts different effects depending on the injury model. There is even less data on the role of Rac1 in kidney tubule development and disease. We and others showed that Rac1 is required for normal development and function of the kidney collecting system (*17*, *18*). In the context of tubule injury, Rac1 contributes to oxalate-induced nephropathy by increasing reduced form of nicotinamide adenine dinucleotide phosphate–mediated cell injury and it enhances transforming growth factor–β1–driven epithelial dysfunction in a UUO model (*85*). Last, Rac1 expression is increased following acute ischemic injury of proximal tubules (*86*). We now show that decreased Rac1 expression correlates with CD tubule damage and morphology in human individuals with chronic kidney disease. Furthermore, we show that loss of Rac1 in the CD makes mice more susceptible to UUO injury and prevents CD recovery in a reversible UUO model. Our observations are consistent with the finding that deleting Rac1 in luminal epithelial cells in the breast caused ductal apoptotic cell shedding and defective epithelial regeneration during the reproductive cycle (*87*). Our data are also consistent with the recent observation that exogenous administration of constitutively active and cell-permeable Rac1 peptides protects the lung epithelium against pathogen-induced injury by stabilizing the actin cytoskeleton (*88*). Thus, like in the mammary gland or the lung, Rac1 is critical in maintaining CD cytoskeletal structure in the setting of injury and injury recovery. We substantially extend these studies by defining how Rac1-dependent actin organization is an essential requirement for normal cell cycling and cell division control.

One of the unique aspects of the reversible UUO model we used in this study is that it allows one to define how Rac1 mediates CD cell proliferation following injury. The CD is an excellent model to understand this process, as unlike many differentiated epithelial cell types (e.g., podocytes) that die after severe injury, quiescent CD cells are able reenter the cell cycle and proliferate (*89*). We found that Rac1 maintains CD cells in the G_2_-M cell cycle phase during repair, suggesting that Rac1 may play a role in “slowing” of cell cycle progression. This was unexpected as failed repair of the injured proximal tubule is caused by a G_2_-M arrest and rapid and sustained proliferation of proximal tubule cells is required for recovery (*8*, *90*). These discrepant data likely indicate cell type–specific control of the cell cycle and underscore our incomplete understanding of the physiologic meaning of cell cycle states. Our data extend prior observations from other model systems that transient stalling or reversible prolongation of the cell cycle in G_2_ is critical for successful repair of tissue injury (*8*, *91*–*94*). Cell cycle slowing after the acute kidney injury phase confers protection against chronic epithelial injury (*92*). The reason for as well as the mechanism of this physiologic cell cycle and G_2_ prolongation has not been defined. We found that Rac1 stabilizes Wee1 to prevent early and rapid mitotic entry during G_2_. Wee1 in yeast and *Xenopus* constitutes a “morphogenesis checkpoint” that puts the brakes on cell cycle progression allowing for the coordination of epithelial morphogenesis and proper actin cytoskeletal organization with mitosis (*95*–*98*). These data suggest that physiologic G_2_ prolongation is a key step in epithelial repair. It is unclear whether this is a conserved element of repair controlled by Rac1 in other organs or cell types.

We found that Rac1-deficient CD cells are unable to undergo normal mitotic rounding and fail the progression through mitosis. This result conflicts with other studies showing that Rac1 deficiency has no effect on mitosis but, instead, its inactivation is required for successful cytokinesis (*99*). In cancer cells, Rac1 activity is suppressed at the cell equator during metaphase and anaphase to promote RhoA-dependent restriction of actomyosin contractility to a narrow zone for efficient membrane ingression in early cytokinesis (*99*, *100*). While we cannot rule out that Rac1 regulation is required for efficient cytokinesis during repair, we did not detect a difference in abnormal polyploidy or aneuploidy (common outcomes of failed cytokinesis) in repairing CD cells either expressing or lacking Rac1. Since we found that CD cells lacking Rac1 are extruded or die in metaphase, it is also conceivable that cells are eliminated before other mitotic defects become apparent. We find that Rac1-driven suppression of RhoA-ROCK–dependent myosin contractility is essential for early G_2_ cell cycling and epithelial mitotic integrity. This finding extends prior experimental evidence that constitutive RhoA activation in epithelial monolayers is sufficient to drive mitotic rounding failure and mitosis-associated cell extrusion (*101*, *102*). Well-known molecular mechanisms exist where mechanically unfit epithelial cells (loss of polarity and flattened morphology) are eliminated from the epithelium by extrusion or apoptosis via lethal p53 induction (*103*). Tight spatial and temporal regulation of Rac1-RhoA antagonism is likely a critical part of this mechanism.

When we investigated the mechanism underpinning the proliferation and mitosis defect, we found that Rac1 is required to stabilize the mitotic entry and G_2_ checkpoint kinase Wee1. Our findings extend previous data showing that Madin-Darby canine kidney cells have a mechanical G_2_-M checkpoint whereby increased E-cadherin tension at lower cell densities leads to myosin contractility-dependent degradation of Wee1 and rapid mitotic entry (*65*). Conversely, when cell density is high, E-cadherin forces are low, and Wee1 is stabilized and G_2_-prolonged (*65*). This endows the epithelial cell cycle with a mechanoresponsive mechanism that is critical in adapting the proliferative response to external stress (e.g. during repair). The upstream regulators of mechanoresponsive G_2_ and mitotic entry regulation in epithelial cells remain undefined. We propose that Rac1—by controlling actomyosin tension and cell-cell junction integrity—could act as a master regulator of Wee1-dependent mechanical G_2_-M checkpoint regulation in polarized epithelial cells. A key question is how Wee1 protein levels are stabilized downstream of actomyosin contractility and cell tension. The specific ubiquitin ligases that target Wee1 in a cell cycle specific manner are known (*67*, *104*, *105*). It would be interesting to define the mechanism whereby epithelial tension and myosin contractility regulate Wee1 and its ubiquitination and proteasomal degradation.

The repairing kidney epithelium is a highly confined space. How morphologically challenging processes such as rapid rebuilding of the actin cytoskeleton and cell division are spatially enabled is unknown. We found here that Rac1-controlled actin cytoskeletal organization is an essential requirement for the repairing CD cells to maintain their shape. Without Rac1 the proliferating epithelium is dysmorphic, premitotic cells in G_2_ fail to go into the appropriate cell cycle pause and are unable to exert pressure on their neighbors and fail to divide. Targeting this mechanism through direct actomyosin inhibition reversed the Rac1-deficient phenotype. Furthermore, we found that prolonged mechanical epithelial injury leads to proteasome-dependent Rac1 degradation. This extends recent experiments showing that volumetric cell compression suppresses Rac1 and tilts the Rac1/RhoA balance toward RhoA with increased myosin activation resulting in maladaptive cytoskeletal remodeling (*76*). Either forced Rac1 activation or myosin inhibition with an ROCK inhibitor was sufficient to recover the compressed cellular phenotype (*76*). These observations and our data highlight Rac1 as a central regulator of mechanical cell states in injury and repair. This raises the question whether “mechanical” therapeutic approaches or mechanobiology-based treatment designs that target the actin cytoskeleton can enhance repair after mechanical kidney damage. This has been proposed and tested in other organs. For example, lungs lacking the small Rho GTPase Cdc42 develop pulmonary fibrosis that is caused by mechanical cytoskeletal tension on alveolar epithelial cells (*106*). Implantation of a custom-made silicone prosthesis alleviated mechanical tension in these lungs, enhanced regeneration, and prevented fibrosis (*106*). In addition, mechanical therapies (e.g. based on shockwaves) are already known to enhance recovery for other injured tissues such as the heart, skin, and bone (*107*). Exploiting mechanoregulatory cues such as extracellular matrix stiffness is an established and powerful avenue to control stem cell differentiation or cancer cell behavior (*108*). However, it should be noted that mechanical targeting approaches (e.g., myosin inhibition) are not cell specific. For example, blebbistatin can affect macrophage motility in kidney injury models that could contribute to its nephroprotective effects. (*45*). Nevertheless, our data suggest that mechanical targeting of actin cytoskeletal-based mechanisms may represent a viable therapeutic principle to enhance postobstructive kidney regeneration, which is worth future exploration.

In conclusion, we provide evidence that Rac1 in kidney CD orchestrates repair by promoting epithelial F-actin organization and orderly actin-dependent mitotic entry and progression. This mechanism reveals how epithelial morphology, cell cycle, and mitotic entry are mechanically coupled.

## MATERIALS AND METHODS

### Mice

All experiments were approved by the Vanderbilt University Institutional Animal Use and Care Committee and conducted in Association for Assessment and Accreditation of Laboratory Animal Care (AALAC)-accredited facilities. All mice were backcrossed onto the C57BL/6N background for multiple generations. Rac1^f/f^ mice were previously described (*109*) and were crossed with AQP2Cre mice (the Jackson laboratory). Age- and gender-matched littermates (Rac1^f/f^ mice) were used as controls.

UUO or reversible ureteral obstruction (R-UUO) was performed as previously described (*24*–*26*) on 8- to 12-week-old male Rac1^f/f^ or AQP2Cre:Rac1^f/f^ mice (20 to 25 g). Mice were anesthetized with ketamine-xylazine and administered preoperative ketofen and isotonic saline. The right ureter was exposed, and clamping was performed on the ureter just distal to the renal pelvis with either sutures (UUO) or an atraumatic microvascular clamp (R-UUO, Fine Science Tools, 3.5-mm × 1-mm, 5- to 15-g press, #00396-01). For irreversible UUO, obstructed kidneys were harvested 10 days after suture placement. For R-UUO, the peritoneal cavity was reopened, and successful ureteral obstruction was confirmed by assessing the presence of hydronephrosis and paleness of the right kidney. The ureteral clamp was removed using a clamp applier (Fine Science Tools, #18040-14), and mice were euthanized on days 5, 7, and 30 after clamp removal. Upon euthanasia, successful decompression was confirmed by visual confirmation of the absence of hydronephrosis and ureteral bulging (fig. S4). A subgroup of animals was intraperitoneally injected with dimethyl sulfoxide (DMSO) (mock), blebbistatin (2 mg/kg, daily; Sigma-Aldrich, #B0560) or Y-27632 (10 mg/kg, daily; Tocris Bioscience #1254), doses within a range previously found to affect organ injury in various animal models (*45*, *110*–*112*). Plasma blood urea nitrogen (Bioassay Systems QuantiChrom Urea Assay Kit, #DIUR-100) was measured per the manufacturer’s instructions, and spot urine osmolality (in milliosmolal per kilogram; freezing point depression osmometer) was measured as previously described (*18*). For BrdU pulse labeling, in vivo mice were injected with BrdU (100 mg/kg; Sigma-Aldrich, #B5002) as a single intraperitoneal injection for 3 to 4 hours before euthanasia. Animal numbers are stated in each legend.

### Histology

Kidneys were removed, coronally cut with a razor in 2- to 3-mm-thick slices, fixed in 4% paraformaldehyde (PFA) for 30 min, and embedded in paraffin. Paraffin tissue sections (5 μm) were dewaxed and rehydrated by successive immersion for 5 min in 100% xylene and 100, 90, and 70% ethanol solutions and stained with hematoxylin and eosin (H&E) for morphological evaluation by light microscopy. Collagen accumulation in kidney sections was determined by staining for Sirius Red and quantified by image analysis using ImageJ [National Institutes of Health (NIH)] as described (https://imagej.nih.gov/ij/docs/examples/stained-sections/index.html). Fibrillar collagen was expressed as percentage area occupied by picrosirius red–positive structures/microscopic field. A minimum of five nonoverlapping kidney cortex areas were analyzed for each kidney. For 2D immunofluorescence, paraffin kidney sections were subjected to heat-induced antigen retrieval in a citrate-buffered solution (BioGenex, #HK086-9K). Sections were blocked, and antibodies were diluted in 3% bovine serum albumin (BSA), 10% normal horse serum, and 0.01% Tween 20 in phosphate-buffered saline (PBS). For mounting, ProLong Gold Antifade was used (Thermo Fisher Scientific, #P10144). CDs were labeled with AQP2 (Cell Signaling Technology, #3487), preconjugated [Alexa Fluor (AF)–488 or AF-647] AQP2 antibodies (Santa Cruz Biotechnology, #sc-515770), or fluorescein isothiocyanate (FITC)–conjugated dolichus biflorus agglutinin (DBA; Vector Labs, #-1031). Terminal deoxynucleotidyl transferase–mediated deoxyuridine triphosphate nick end labeling (TUNEL) assay was performed on paraffin sections as described by the manufacturer’s instructions (Sigma-Aldrich, #11684795910). Other antibodies include anti-BrdU (Cell Signaling Technology, #5292), pH3 (S10) antibody (Active Motif, #39098), mouse anti–E-cadherin (BD Biosciences, #610181), pMLC (Cell Signaling Technology, #3671), and FITC preconjugated anti-Ki-67 (Thermo Fisher Scientific, #11-5698-82). Secondary antibodies included the following: AF-488 anti-rabbit (Thermo Fisher Scientific, #A21206), AF-647 anti-rabbit (Thermo Fisher Scientific, #A21245), AF-647 anti-mouse (Thermo Fisher Scientific, #A212236), and AF-555 anti-rat (Thermo Fisher Scientific, #78945). 4′,6-Diamidino-2-phenylindole (DAPI) (Cell Signaling Technology, #4083) was routinely added at a 1:500 dilution to secondary antibody incubation to label DNA. For all histological quantifications, scatter plot dots represent the average per mouse with at least 10 measurements per mouse.

### Determination of mitotic stages

Analysis of mitotic stages in vitro and in vivo was performed using high-resolution confocal microscopy images. In vivo prophase was defined as a cell with nuclear positivity for pH3 and condensed chromatin relative to interphase cells. Metaphase was defined as a pH3-positive cell with DNA aligned at the center of the cell. In time-lapse imaging in vitro, mitotic condensation of prophase DNA and the increasing condensation during metaphase were used to determine the transition from prophase to metaphase. Anaphase was generally defined as a cell with an oblong membrane containing a clear separation of DNA to two poles. Telophase was defined as cells that have undergone membrane division with a clear separation between DNA and membrane within two daughter cells. For visualization of live imaging data, mitotic cells were manually segmented using Fiji/ImageJ, and a green lookup table (LUT) was applied to mitotic DNA. All groups were processed identically.

### Optical clearing

Optical clearing was performed as previously described with some modifications (*28*, *113*, *114*). In vivo transcardiac perfusion with 10 to 15 ml of PBS infused over 5 min was used to wash out blood. The kidney was removed and coronally sliced with a razor at 2 to 3 mm in thickness, the cortex was removed, and slices were incubated for 30 min in freshly prepared 4% PFA rocking at room temperature, washed three times in PBS, and then incubated in blocking buffer (2% BSA, 0.2% Tween 20, 0.2% Triton X-100, and 0.02% sodium azide) for 4 to 5 hours at room temperature. Next, sections were incubated in primary antibody (at least 1:50 dilution depending on the primary) in blocking buffer for 3 days at room temperature on a rocking platform, washed, and then incubated in secondary antibodies (+DAPI) for 3 days at room temperature. Slices were then washed thoroughly three times in PBS with 2 to 3 hours per washing step. Slices were dehydrated in a methanol series (25, 50, 75, 100, and 100%), 5 min each step, and transferred into BABB solution (benzyl alcohol and benzyl benzoate at a 1:2 ratio) in an Eppendorf tube for 20 min. Once the slices were translucent, they were placed into a glass-bottom dish (MatTek, #P35-1.5-14-C) containing 100 μl of BABB solution for confocal imaging.

### Confocal microscopy and image processing

Images were collected with confocal microscopy at super-resolution using a Zeiss LSM 980 confocal microscope equipped with an inverted Axio Observer 7 and Airyscan 2 detector. The objective used was a 63×/1.4 numerical aperture (NA) Plan Apochromat oil or 10×/0.50 NA Plan Apochromat (for low-powered scanning of Ki-67–labeled and optically cleared medullary slices). Airyscan super-resolution images were acquired under identical settings for all groups and images. Acquisition and 2D Airyscan processing of acquired images was done using ZEN Blue software (Carl Zeiss). For optimal optical sectioning 3D reconstructions, auto Z brightness corrections were applied that corrects for differences in optical tissue density during deep imaging. To computationally subtract bleed-through between channels, spectral profiles of each fluorescence channel were analyzed, and linear unmixing was performed using ZEN Blue. Next, image stacks were exported into Fiji/ImageJ for brightness/contrast adjustments and LUT assignment. Image stacks were then exported into Imaris (Bitplane). For AQP2/Ki-67 double labeled medullary slices, AQP2^+^ tubules were converted into surfaces using automatic segmentation and thresholding. Next, Ki-67 was converted into spots using the “create spots” function and thresholding by quality. Last, the Imaris XTension (written in MATLAB), “spots close to surface” was activated and run with a zero-distance threshold to filter the spots inside AQP2^+^ surfaces. For display, Ki-67 was shown as spots, while for AQP2, the original 3D reconstruction is shown. For tubular 3D F-actin reconstructions, in vivo image stacks were exported into Imaris. A surface around subapical F-actin was manually created for the whole-stack segmentation. Next, a mask was created on the F-actin channel (AF-647), and the voxels outside the subapical actin surface were set to zero. This results in displaying apical F-actin only. To display basolateral F-actin or total tubular F-actin, another surface was created around the whole tube, and subapical, as well as actin outside the tube, was masked. For mitotic metaphase F-actin surface reconstruction, Imaris software was used as outlined above using surface creation and manual segmentation.

### Thick frozen sectioning and F-actin labeling in vivo

For actin cytoskeletal preservation in live tissue, kidneys were perfusion fixed in 50:50 mix of 2% PFA and 2% glutaraldehyde in PBS, and the renal papillae including the upper inner medulla were dissected. For cryoprotection, tissue was then incubated in 30% sucrose for 48 hours, after which they were transferred into cryomolds for optimal cutting temperature (OCT) embedding (Fisher Scientific, #23-730-571). Thick cryosectioning was performed at 50 μm in thickness using a Leica cryostat. Sections were dried, rehydrated, and incubated with AF-647–phalloidin (Thermo Fisher Scientific, #A22287; 1:50) for 24 hours. For simple immersion, aqueous-based optical clearing sections were incubated in refractive index matching solutions (40 g of Histodenz in 30 ml of 0.02 M phosphate buffer with 0.01% sodium azide, brought to a pH of 7.5 with NaOH) for 2 to 3 hours or until sections became translucent. 3D super-resolution confocal imaging and processing was performed as outlined above. For assessment of total actin content, the mean global gray value intensity (of background normalized *Z*-stacks for each dataset) of maximum intensity projections (five *Z*-slices each) was obtained using ImageJ. For apical F-actin intensity, a similar operation was performed, but the mean gray value of regions of interest drawn around subapically segmented F-actin was used. To obtain the apical F-actin major axis length, orthogonal projections of whole tubular Z-stacks sliced across the median were generated. Longitudinal cell-to-cell junction distances were then measured and calculated using ImageJ’s line tool. Annotated examples of histological quantifications are further illustrated in fig. S32.

### Cell culture and cell cycle synchronization

Rac1^−/−^ CD cells were generated and cultured as previously described (*18*). To assess mitotic rounding in polarized monolayers, cells were grown on Transwell inserts consisting of polyvinyl pyrrolidone–free polycarbonate filters with 0.4-μm pores (Corning, #3460) as previously described. Confluent cells were fixed after wounding (see below) in 4% formaldehyde and processed for AF-647–phalloidin, activated actomyosin (pMLC S19, Cell Signaling Technology, #3671), and DAPI labeling. Cell cycle synchronization at the G_1_-S boundary was performed using a double thymidine block as previously described (*48*). Briefly, thymidine was added at a final concentration of 2 mM to CD cells at 50 to 60% confluency for 18 hours. Next, cells were washed in PBS once and regular prewarmed medium [Dulbecco’s modified Eagle’s medium (DMEM) and 10% fetal bovine serum (FBS)] was added back for 9 hours. After this, a second round of 2 mM thymidine was added for 18 hours after which cells were washed in PBS twice and incubated in regular medium for timed collections. Cell cycle synchronization in G_2_ at the G_2_-M boundary was performed by treating CD cells with 2 μM Cdk1 inhibitor RO-3306 (R&D Systems, #4181) for 20 hours, washed twice with PBS, and then cultured in full regular medium. Other cell culture treatments included nocodazole (Tocris Bioscience, #1228) at 100 ng/ml or the Wee1 inhibitor Adavosertib (MK-1775, Tocris Bioscience, #7589) at 1 μM, blebbistatin (Sigma-Aldrich, #203391) at 5 μM, or the ROCK inhibitor Y-27632 at 1 μM. For cell treatments, all drugs were dissolved for stock solutions and used according to the manufacturer’s recommendations. Solvents for stock solutions were used as negative control treatments in respective experiments. All in vitro experiments were repeated three times unless otherwise indicated. For lentiviral transfections of shRNA, the following vectors were ordered prepackaged in lentiviruses (VectorBuilder). For GEF-H1 (Arhgef2), pLV[shRNA]-EGFP:T2A:Puro-U6>mArhgef2 (ID: VB900050-0695dkh), and for Hace1, pLV[shRNA]-EGFP:T2A:Puro-U6>mHace1 (ID: VB900068-4913bgh). For the transduction of target cells, mouse CD cells were cultured to 50% confluency in DMEM (10% FBS). The recommended amount of virus per the manufacturer’s instructions was used and added in virus-containing Opti-MEM with polybrene. Successful transfection was monitored using the green fluorescent protein (GFP) signal under the ZOE Fluorescent Cell Imager (Bio-Rad) and confirmed by immunoblotting for the target protein.

### Wounding assay

Scratch assays were performed as previously described (*18*). Briefly, CD cells were grown to confluence on Transwell inserts consisting of polyvinyl pyrrolidone–free polycarbonate filters with 0.4-μm pores (Corning, #3460). For wounding, confluent epithelial cell sheets were scratched with a 10-μl pipet tip. An area adjacent to the scratch was monitored using the ZOE Fluorescent Cell Imager (Bio-Rad). As soon as the scratched area was close and the area was fully covered with cells (15 hours for Rac1^f/f^ and 24 hours for Rac1^−/−^ CD cells), the cell sheets were fixed, F-actin was visualized by phalloidin, and DNA with DAPI and mitotic cells were labeled with anti-pH3.

### Cell stretch and compression

Cells were stretched as previously described (*33*, *115*). Briefly, to expose cells to uniaxial noncyclical mechanical stretch, CD cells were grown to confluency on collagen-coated flexible silicone six-well culture plates within a gasekt (Flexcell International Corporation, Burlington, NC, USA) in regular full DMEM. A controlled vacuum is applied resulting in the membrane deformation across the planar face of the postcreating uniform axial strain. See also schematic representation in Fig. 4C. Static stretch was applied by increasing the percent elongation (degree of stretch) from zero to 10 at ~0.3 to 0.5% per min during the 30 min (ramp up) and then holding at 10% elongation for 3 hours. At the end of the protocol, the percent elongation was decreased to zero over 5 to 10 min before removing plates from the vacuum manifold. Control plates were not subjected to stretch. Cell compression was performed as previously described (*76*–*79*). Briefly, low–gelling temperature agarose (Sigma-Aldrich, #A9045) was dissolved in PBS at 3% concentration by heating in a microwave oven, followed by mixing the solution with DMEM cell culture medium with 20% FBS in a 1:1 ratio to form a 1.5% agarose gel solution containing full medium (DMEM and 10% FBS), and poured into a round petri dish. Round agarose cushions (diameter, ~20 mm) were cut, gently placed on top of the cells, and weighted down with around 25 g of stainless-steel balls in which prior studies have shown to create ~5.8 mmHg of constant pressure. For enrichment of ubiquitinylated Rac1 in compressed CD cells, immunoprecipitation was performed as previously described (*116*). Briefly, cells were harvested with M-PER after compression and gently sonicated, and debris was removed by centrifugation (13,000 rpm for 10 min). Next, 10 μl of immunoprecipitation-validated Rac1 antibody (ProteinTech, #24072-1-AP) was added to 150 μg of protein and incubated under agitation at 4°C overnight. Then, 80 μl of 50% slurry of Protein A agarose beads was added (Thermo Fisher Scientific/Pierce Protein A Agarose, #20333) and incubated overnight at 4°C. Beads were then precipitated, washed, and resuspended in 1× SDS-loading buffer for Western blotting of anti-ubiquitin (Cell Signaling Technology, #58395 or #3936).

### Live mitosis imaging

For live imaging of mitosis, control and Rac1^−/−^ CD cells were grown to confluence on glass-bottom live imaging MatTek dishes (MatTek, #P35G-1.5-14-C) precoated with growth factor–reduced Matrigel (Corning, #356230). Wounding was performed as described above. Upon wound closure and 1 hour before time-lapse imaging, the following live-cell fluorogenic labeling probes were added according to the manufacturer’s instructions (at 1× dilution of a 1000× stock) in phenol red–free complete DMEM: SPY650-DNA (Cytoskeleton, #CY-SC501) to label DNA and SPY555-actin (Cytoskeleton, #CY-SC202) to label F-actin. High-resolution live confocal imaging was carried out using a Zeiss LSM 980 microscope using a 63×/1.4 NA Plan Apochromat oil objective and equipped with a 37°C humidified incubator supplied with 5% CO_2_. Images were acquired at in the Airyscan Multiplex SR-8Y mode with subsequent deconvolution. In general, one image every 10 min with 10 optical sections (every 1 μm) per time point per frame was acquired. For visualization purposes, 4D confocal stacks were subsequently exported into Fiji/ImageJ for manual segmentation of mitotic chromosomes and green LUT reassignment. A minimum of 10 mitotic events per condition were recorded. Mitotic metaphase delay was defined as the inability to progress through and successfully complete metaphase within 60 min. Treatments (Wee1 inhibitor MK-1775 at 1 μM or blebbistatin at 5 μM) were added 30 min before time lapse imaging in phenol red–free complete DMEM.

### RhoA activity assay

RhoA GTPase activation assay was performed using the colorimetric G-LISA RhoA activation assay (Cytoskeleton, #BK124) according to the manufacturer’s instructions and as previously described (*117*). After the experimental treatments, cells were rinsed with ice-cold PBS and homogenized in ice-cold lysis buffer. Protein was quantified and sample concentration adjusted to 1 μg/μl using BCA Protein Assay Kit (Thermo Fisher Scientific, #23225). Binding buffer was added, and assays were performed using 20 μg of protein per well. Target binding to the Rho GTP-binding protein coated well was facilitated by 30 min of incubation at 4°C with shaking, followed by washing. Antigen-presenting buffer was added for 2 min and removed, and samples were incubated with the anti-RhoA antibody (1:250) for 45 min at room temperature. Samples were washed three times and incubated with horseradish peroxidase–conjugated secondary antibody for 45 min at room temperature. Horseradish peroxidase detection reagent was added, the samples were incubated briefly, and absorbance was measured at 490 nm using a microplate reader (Molecular Devices).

### 3D spheroid culture

3D CD spheroids were generated as previously described (*118*). Briefly, a CD cell suspension of 100,000 cells/100 μl in complete medium was mixed with Matrigel (Corning, #356230) in a 1:1 ratio. 200 μl of the cell:Matrigel mix were added to the media chambers of an of an eight-well glass slide (Millipore, #PEZGS0816). Upon solidification in the incubator (at 37°C and 5% CO_2_) for 30 min, 200 μl of prewarmed complete medium was added to the hardened cell:Matrigel mix. Cells were cultured for 4 days, while monitoring spheroid formation using a cell culture inverted light microscope. Medium was changed daily. Treatments (Wee1 inhibitor MK-1775 at 1 μM or blebbistatin at 2 μM) were added 16 hours before spheroid harvesting. For spheroid harvesting, the gels were washed twice with calcium-free PBS, the Matrigel was dissolved, and the spheroids were fixed by incubating the gels with fresh 4% PFA for 30 min at room temperature. Permeabilization, blocking, immunocytochemistry, and immunofluorescence were then performed per standard protocols. Spheroids were stained for F-actin using AF-647–conjugated phalloidin, α-tubulin (Cell Signaling Technology, #2144), and DNA (DAPI) and imaged using a Zeiss LSM 980 in super-resolution multiplex mode, followed by mitotic F-actin surface reconstructions using Imaris (Bitplane).

### Traction force microscopy

Traction force microscopy of CD cell layers was performed as briefly described with some modifications (*119*–*121*). For polydimethylsiloxane (PDMS) substrate preparation, the two-part PDMS material Sylgard-184 was used according to the manufacturer’s instructions. On the basis of previous characterizations, we chose an elastomer-to-curer ratio of 80:1 that gives rise to a substrate stiffness of ~9 kPa. One hundred microliters of the PDMS substrate mixture was applied to the coverslip of glass-bottom imaging dishes (MatTek) using a cut P200 tip and cured for 2 hours at 80°C. Next, the PDMS surface was silanized by adding 2 ml of 5% (v/v) 3-aminopropyltriethoxysilane (APTES) solution (in 100% ethanol) on top of the cured PDMS surface for 10 min. APTES was removed, and the PDMS surface was washed three times with 100% ethanol and dried at 80°C for 30 min. Next, a 0.05% (v/v) fluorescent Nanobead solution (FluoSpheres carboxylate-modified microspheres, Thermo Fisher Scientific, #F8811; diameter, 0.2 μm) was prepared in water and gently sonicated in a water bath sonicator. To functionalize the PDMS substrate with nanobeads, the bead solution was incubated in the PDMS dish for 5 min at room temperature. After the bead solution was removed, the plate was washed with water three times and again dried at 80°C for 30 min. To inactivate the bead surface, the PDMS substrate was incubated in 100 mM tris solutions for 10 min at room temperature and again dried. PDMS dishes were then coated with Matrigel as outlined above and sterilized using 1% pluronic, after which CD cells were seeded and grown to 100% confluence. Upon addition of live-imaging dyes (SPY probes as outlined above), frames of cells were monitored using a Zeiss LSM 980 confocal and the live imaging setup as described above to detect chromosome condensation upon mitotic entry. Stacks of five images with the fluorescent beads were obtained during prometaphase. For the references image, fluorescent bead image stacks were obtained of the same frame after addition of 10% SDS (reference image without cells). Bead image stacks with and without cells were aligned using the slice alignment plug-in (ImageJ), and gel deformation was calculated using the particle imaging velocimetry plug-in (ImageJ). Forces were determined and force maps were generated using the Fourier transform traction cytometry plug-in (ImageJ).

### Flow cytometry

Flow cytometry and DNA content–based cell cycle assessment on kidney tissue was performed as previously described with some modifications (*30*). Briefly, kidneys were harvested and coronally sliced, and slices with an equal weight between groups were minced in fresh 4% PFA on ice with 1:100 protease inhibitors. Tissue was then washed and pelleted by brief centrifugation, and additional mechanical tissue dissociation was performed using the gentleMACS Dissociator. The tissue was then resuspended in a detergent solution (0.2% Triton X-100) to permeabilize for 30 min at room temperature. After pelleting and washing in PBS, the tissue was resuspended in dissociation solution [collagenase type 2, 1 mg/ml (Thermo Fisher Scientific, #17101015) and dispase II, 1 mg/ml (Sigma-Aldrich, #D4693)] in 1 mM CaCl_2_ and 0.5 mM MgCl_2_ and incubated at 37°C for 1 hour. Samples were then placed on ice and passed through a 70-μm cell strainer (Thermo Fisher Scientific, #08-771-2). Cells were centrifuged (5000*g* for 5 min at 4°C) and resuspended in 1.5 ml of 0.5% FBS/PBS. Cell concentrations were then normalized to 1 million cells per ml across all samples using an automated cell counter (AE Bios, Cellometer K2). Samples were incubated for 10 min at room temperature with Fc blocking antibody, followed by incubation with anti-AQP2 (Cell Signaling Technology, #3487) and anti-pH3 (both 2 μl per 1 Mio cells) and DAPI (1:200). Flow cytometry data were acquired with a BD LSRFortessa analyzer at the Vanderbilt University Medical Center Flow Cytometry Shared Resource. Data files were analyzed by floreada.io, and we performed cell cycle analysis by gating a plot of side or forward scatter versus DAPI staining. All runs included staining controls (secondary antibodies only) and compensation controls. Cell cycle assessment in vitro was performed as previously described (*122*). Briefly, CD cells were grown according to the required experimental condition and detached using Accutase. Cell aggregates were carefully broken up by pipetting up and down. The cells were then spun down at 1500 rpm for 5 min. The cell pellet was resuspended in ice-cold 70% ethanol while vortexing, and cells were fixed in ethanol overnight. Cells were then spun down, ethanol was removed, and the cell pellet was washed in PBS. Cells were then resuspended in propidium iodide staining solution [0.1% Triton X-100 in PBS, deoxyribonuclease-free ribonuclease (100 μg/ml), and propidium iodide (50 μg/ml)] and subjected to flow cytometry.

### Immunoblotting

Immunoblotting was performed as previously described (*18*). Briefly, cell lysates were prepared using M-PER reagent (Thermo Fisher Scientific; #78501). Lysates were resolved in 10 and 12% or gradient 4 to 20% SDS–polyacrylamide gel electrophoresis depending on protein size and then transferred to nitrocellulose membranes. Membranes were incubated with the primary antibody, followed by IRDye fluorescent dyes secondary antibodies, and a LI-COR Biosciences Odyssey infrared imaging system was used for detection. Immunoreactive bands were quantified by densitometry analysis using ImageJ or Image Studio Lite. To quantify levels of protein phosphorylation, optical density of bands was normalized to total protein or β-actin. For kidney tissue analysis, papillae were removed and mechanically lysed in T-PER reagent (Thermo Fisher Scientific, #78510) using a Polytron homogenizer, centrifuged, and then subjected to immunoblotting. Primary antibodies used are pH3 (Cell Signaling Technology, #9701), cleaved caspase 3 (Cell Signaling Technology, #9664), Wee1 (Novus Biologicals, #NBP1-33506), actin (Cell Signaling Technology, #4967), cyclin B1 (Cell Signaling Technology, #4138), Rac1 (Millipore, #05-389), α-tubulin (Cell Signaling Technology, #2144), Cdk1 (Cell Signaling Technology, #77055), and phospho–(Y15) Cdk1 (Cell Signaling Technology, #4539). The secondary antibodies were IRDye 800CW anti-rabbit (#926-32213 and #926-32211), IRDye 800CW anti-mouse (#926-32212 and #926-32210), IRDye 680RD anti-rabbit (#926-68073 and #926-68071), and IRDye 680 anti-mouse (LI-COR Biosciences, #926-68072 and # 926-68070).

### Two-photon laser-induced injury in vitro

Induction of two-photon induced cellular microlesions was performed as reported with some modifications (*123*). Briefly, control and Rac1^−/−^ CD cells were grown to confluent monolayers on Transwell inserts (Corning, #3460). The Transwell filters containing the confluent monolayers were excised using a razor, inverted, and mounted onto the coverslip of a complete phenol-free medium–containing glass-bottom dish (MatTek, #P35-1.5-14-C). F-actin was labeled using SiR-actin (Cytoskeleton, #CY-SC001) or SPY555-actin (Cytoskeleton, #CY-SC202) according to the manufacturer’s instructions. This was mounted on the stage of a Zeiss LSM 780 equipped with a tunable-range coherent chameleon Ti:sapphire multiphoton laser. A 63×/1.40 NA Plan Apochromat oil Differential Interference Contrast (DIC) objective was used for imaging. Experiments were performed at 37°C in 25 mM Hepes. After collecting control images, a small rectangular region (2 μm × 3 μm) of a single cell was scanned at high Ti-sapphire laser power (730 nm; 80% intensity) for 25 iterations (duration, 8 to 10 s). F-actin images of the same frame were subsequently captured as indicated in the figure legend.

### Human samples

Formalin-fixed, paraffin-embedded tissue sections were obtained from deidentified tumor nephrectomy (renal cell cancer) specimens through the Department of Pathology at Vanderbilt University Medical Center (VUMC). Control Images were obtained from the nontumor healthy portion of the section. CKD biopsy specimens were randomly selected and had to have at least CKD stage 2 and 10 to 20% tubulointerstitial fibrosis. This use of human deidentified samples was approved by the VUMC institutional review board.

### Statistics

Data are shown as means ± SD or SEM. Unpaired two-tailed *t* test was used to evaluate statistically significant differences (*P < 0.05) between two groups. One-way analysis of variance (ANOVA) was used to test statistical significance among multiple groups. Post hoc comparisons of ANOVA were corrected with the method of Tukey. For fold change calculations, the control number was normalized to 1 for each comparison and does not have an error bar. Data distribution was assumed to be normal, but this was not formally tested.
