# Supplementary material for: Rac1 promotes kidney collecting duct repair by mechanically coupling cell morphology to mitotic entry
Source: Sci Adv. 2024 Feb 7;10(6):eadi7840. doi: 10.1126/sciadv.adi7840 (PMC10849615; doi:10.1126/sciadv.adi7840)
Supplement: Supplementary file 1 — Figs. S1 to S32 [file sciadv.adi7840_sm.pdf]

Science Advances
AAAS

Supplementary Materials for

Rac1 promotes kidney collecting duct repair by mechanically coupling cell morphology to mitotic entry

Fabian Bock *et al.*

Corresponding author: Fabian Bock, fabian.bock@vumc.org; Roy Zent, roy.zent@vumc.org

Sci. Adv. **10**, eadi7840 (2024)
DOI: 10.1126/sciadv.adi7840

The PDF file includes:

Figs. S1 to S32

Other Supplementary Material for this manuscript includes the following:

Movies S1 to S7

Supplemental Figure 1:

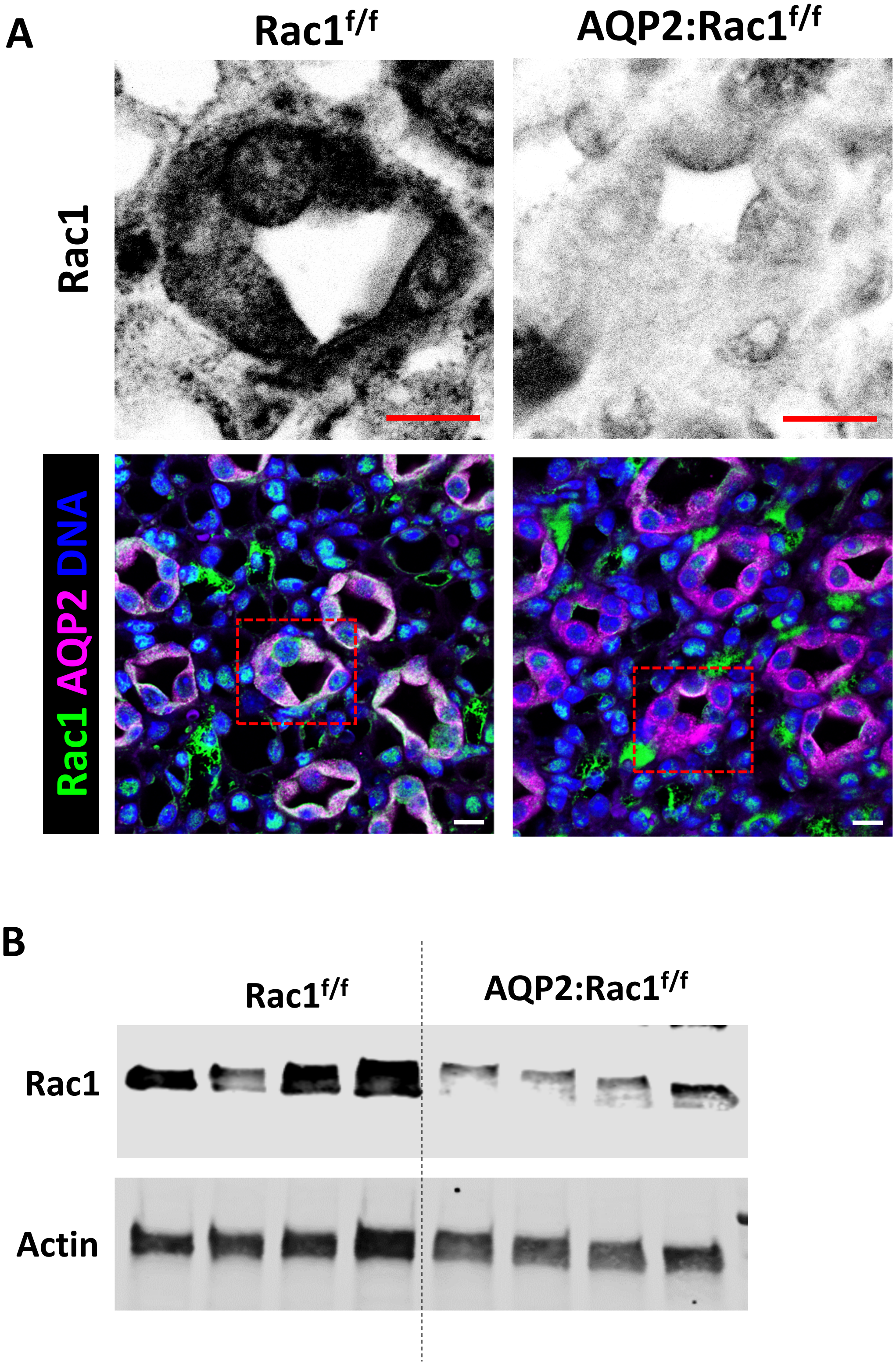

Supplemental Figure 1. (A,B) Confirmation of Rac1 (green) deletion in AQP2+ (magenta) collecting ducts at 10 weeks (without injury) by immunofluorescence (A) and immunoblotting of papillas (B). The top panels in A are color-inverted insets as outlined by a dashed red box in the bottom panel of the green Rac1 channel. Scale bars 10 μm.

Supplemental Figure 2:

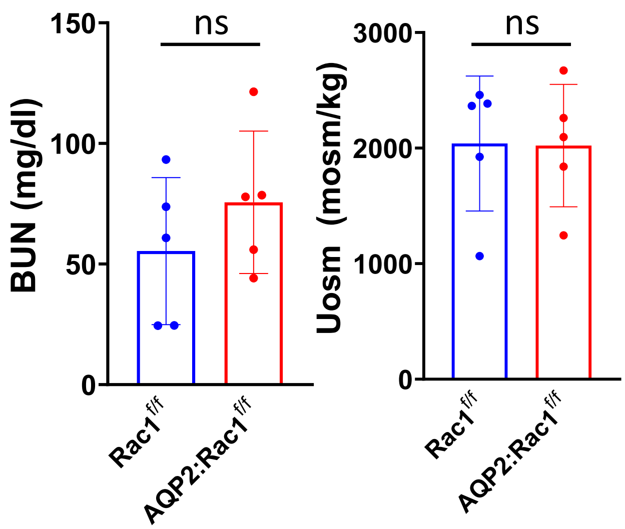

Supplemental Figure 2. Assessment of plasma BUN (blood urea nitrogen, mg/dl) and spot urine osmolality (mosm/kg) in the indicated groups at baseline (no injury). n= 5 mice per group. Mean ± SD. ns: not significant.

Supplemental Figure 3:

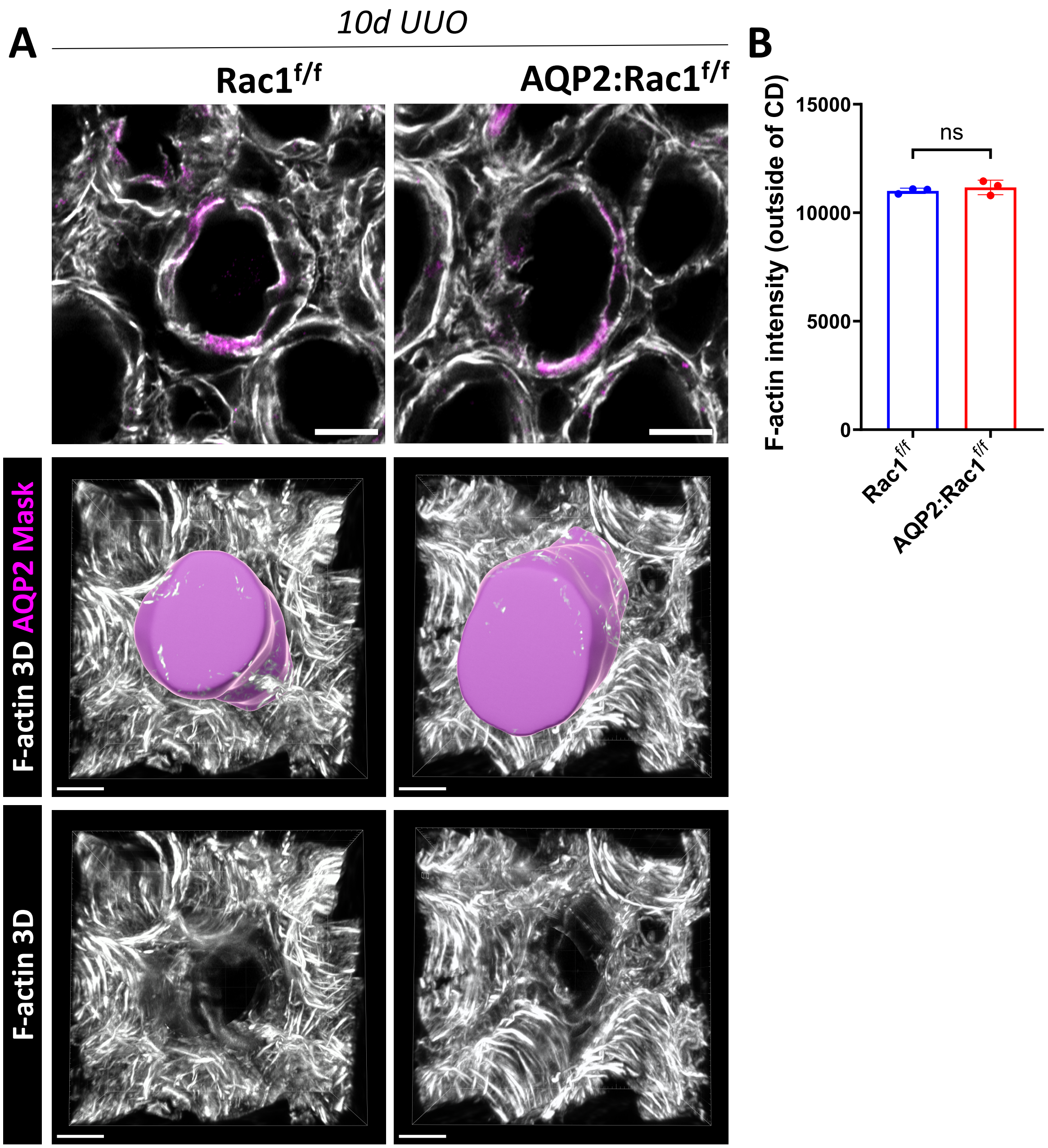

Supplemental Figure 3. (A,B) Thick fresh frozen medullary kidney slices stained for AQP2 (CDs) and F-actin of obstructed (10d UUO) control and AQP2:Rac1$^{f/f}$ kidneys. First row depicts a cross-section, second row shows the F-actin channel in 3D with a surface around the centered AQP2+ CD (AQP2 Mask), shows 3D F-actin outside of the masked CD. Scale bars 10 µm. **(B)** Quantification of total tubular (whole thickness of max intensity projections) F-actin content outside the CD. N=3 mice per group with each dot in the scatter plots representing an individual sample. Bars are mean ± SD. ns: not significant.

Supplemental Figure 4:

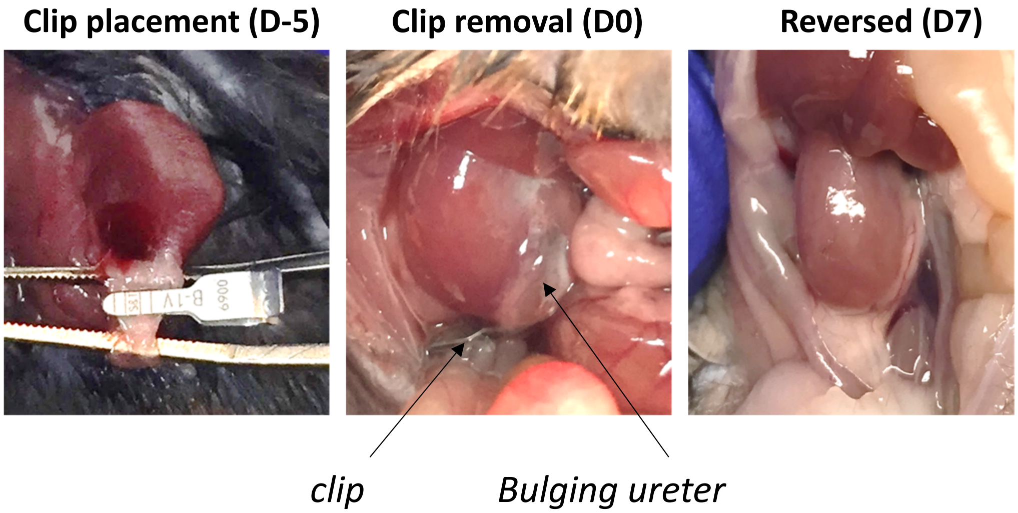

Supplemental Figure 4. Representative macroscopic images showing the surgical reversal procedure of ureteral clip placement, subsequent removal when ureter bulging became visible (day 5) and visual confirmation of decompression (“reversed”) one week after clip removal.

Supplemental Figure 5:

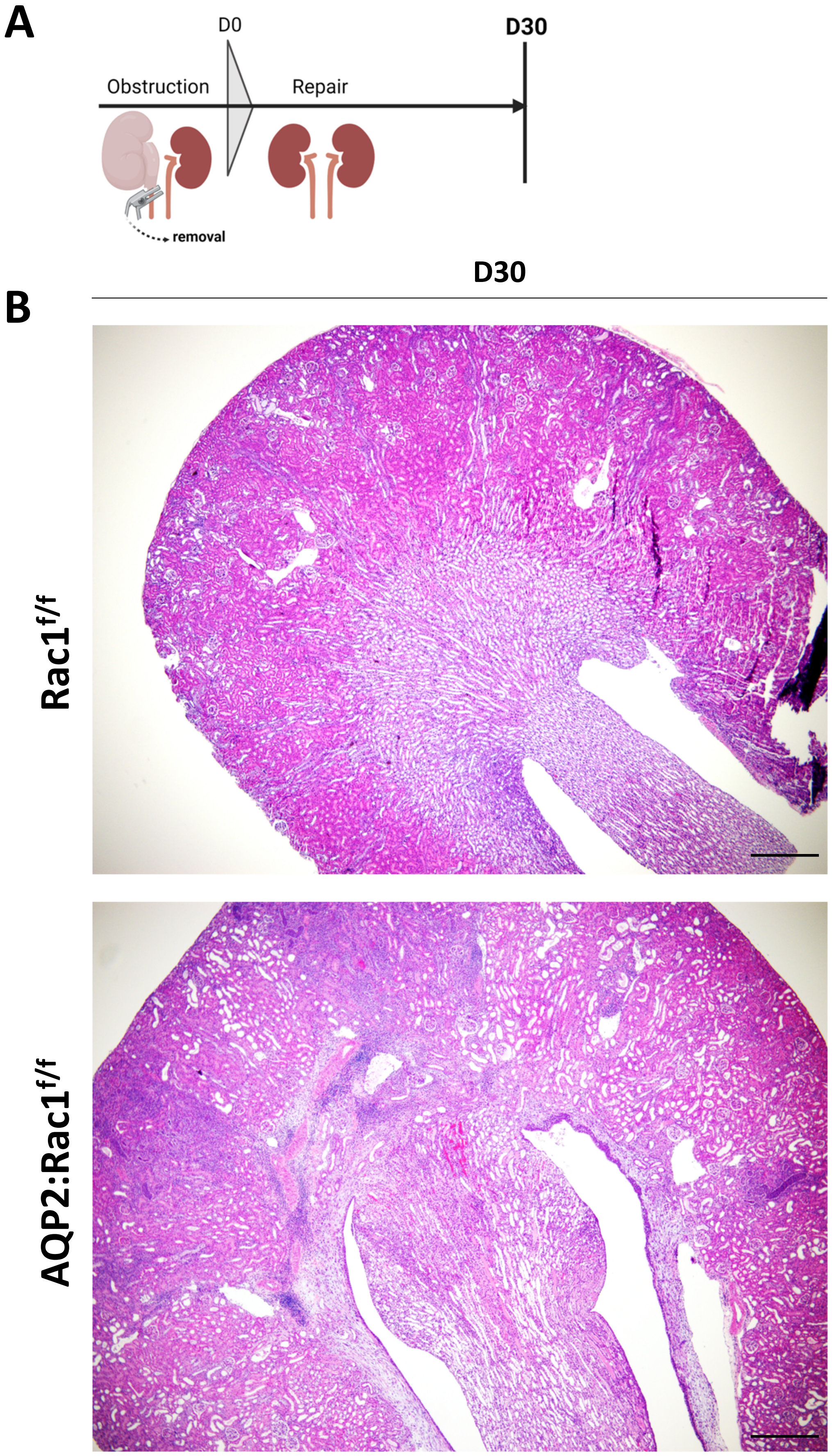

Supplemental Figure 5. (A,B) Histological assessment of Hematoxylin and Eosin-stained (H&E) paraffin kidney sections one month (D30) after reversal of obstruction as depicted in **A** (Created with Biorender.com). Scale bar 200µm.

Supplemental Figure 6:

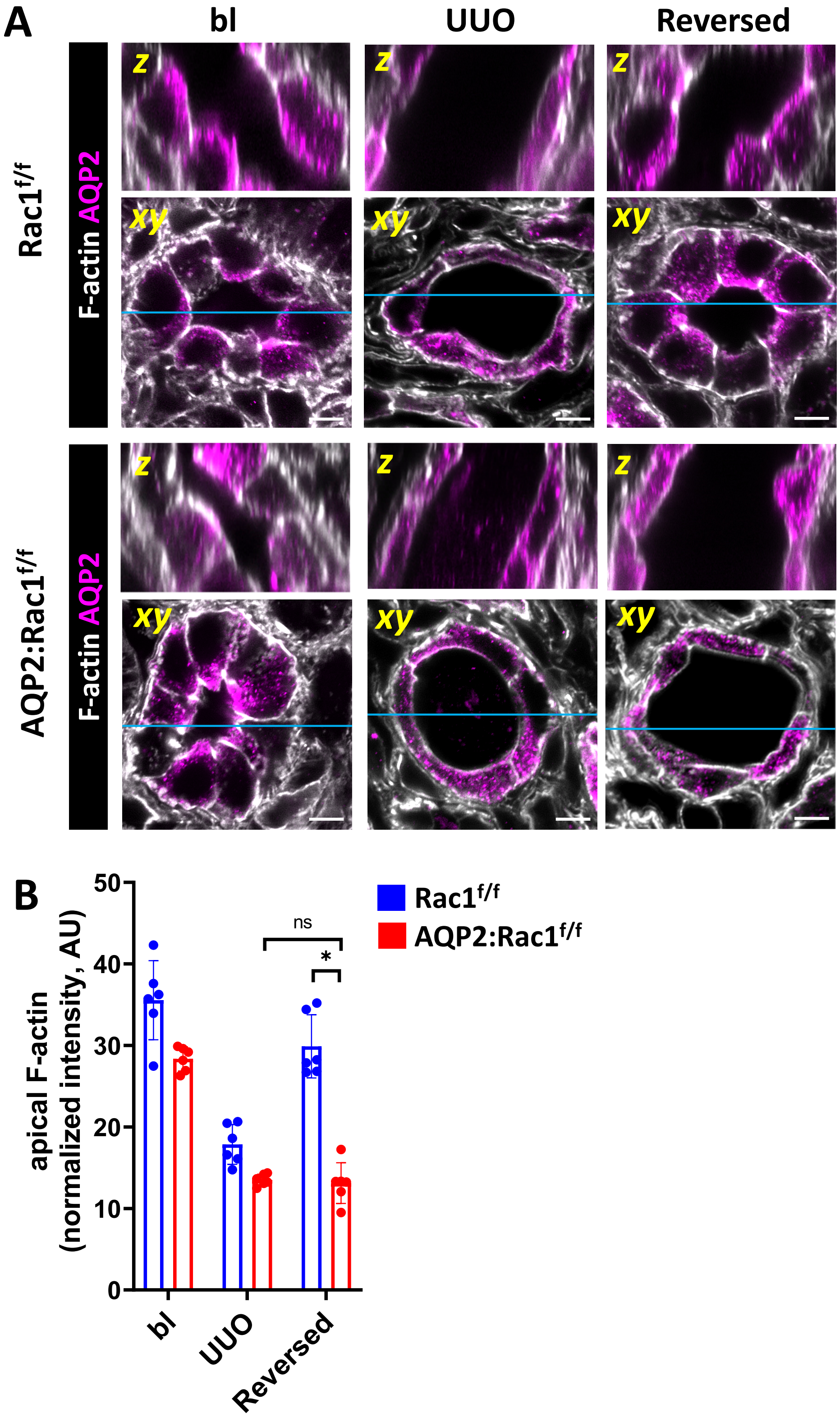

Supplementary Figure 6. **(A)** Cross-sections of F-actin-labelled thick frozen sections corresponding to Figure 2H with the medullary CDs labeled with AQP2 (bottom row) of baseline ("bl"), obstructed ("UUO") and de-obstructed ("Reversed") Rac1$^{f/f}$ and AQP2:Rac1$^{f/f}$ mice. Orthogonal Z-slices (as indicated by a continuous blue line in the cross-section) are shown in the top panels (scale bar 5 µm). N=6 mice per group. **(B)** Quantification of normalized apical F-actin intensity (AU, arbitrary units) in **A** with dots representing an individual mouse. Bars are mean ± SD.

Supplemental Figure 7:

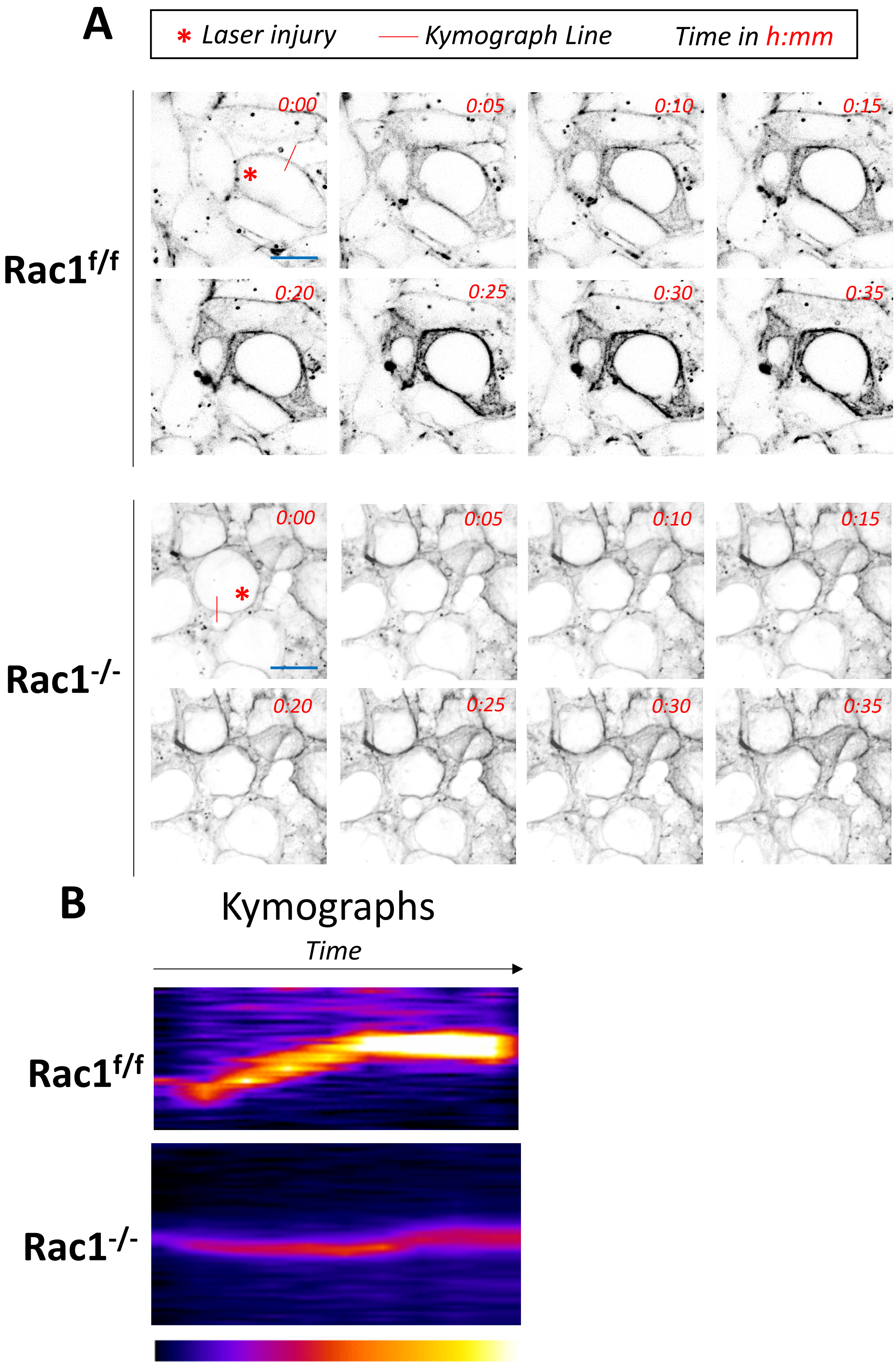

Supplemental Figure 7. (A) Confocal time lapse imaging of live F-actin labeled (SiR-Actin, black) laser-injured Rac1$^{f/f}$ and Rac1$^{-/-}$ CD cell monolayers. * indicates the point of laser injury. **(B)** Kymographs performed along the solid red line in **A** and fire LUT converted using ImageJ with the intensity scale displayed at the bottom. Scale bar 10 µm

Supplemental Figure 8:

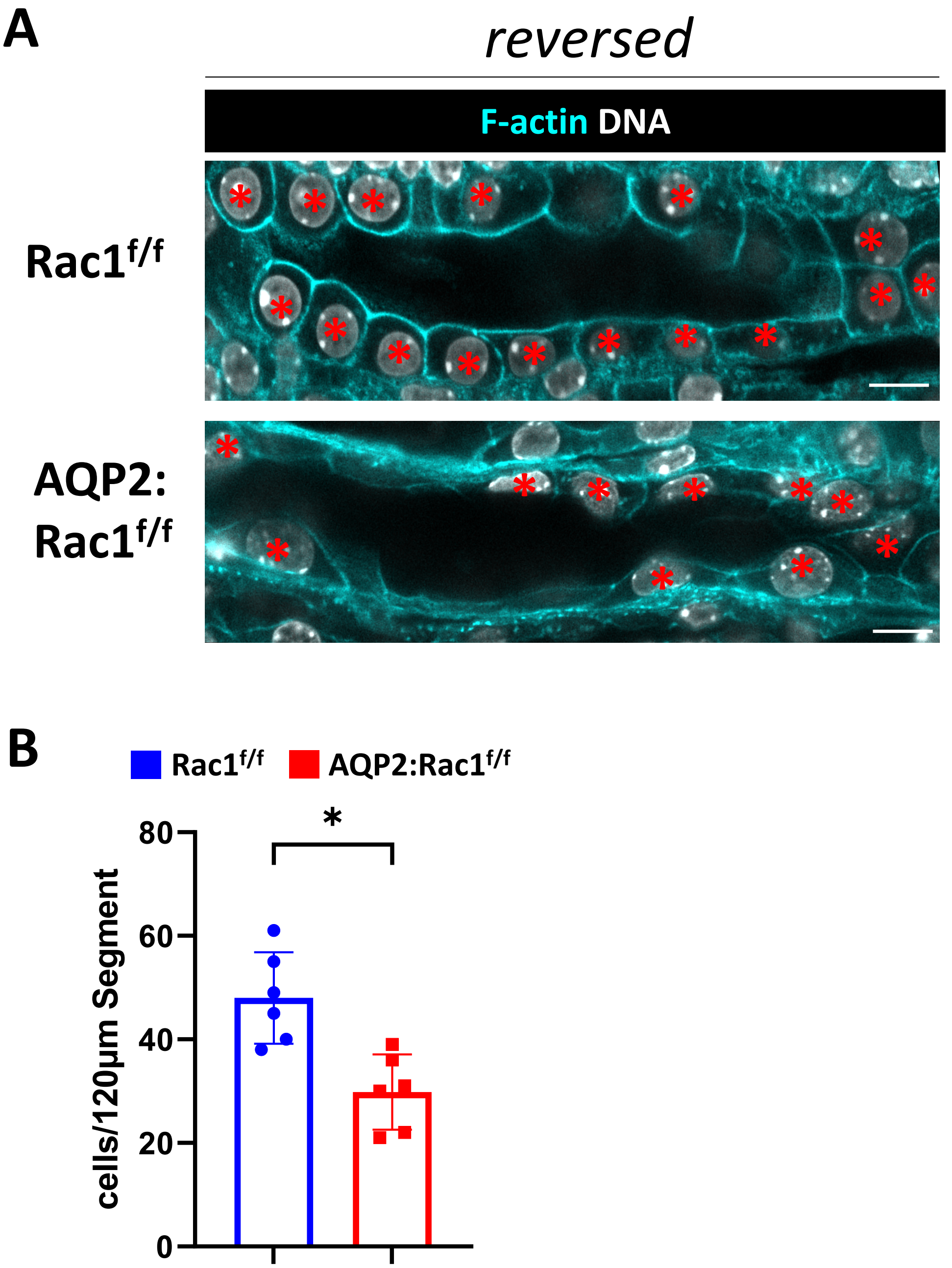

Supplemental Figure 8. (A,B) Assessment of cellularity of F-actin (cyan) and DNA (white) labelled upper inner medullary CDs of reversed control and AQP2:Rac1$^{f/f}$ mice. Images are representative of 6 mice per group. * indicates single cells that were counted and quantified in **B** with dots representing individual mice and bars ± SD. Scale bar 10 µm. *P < 0.05.

Supplemental Figure 9:

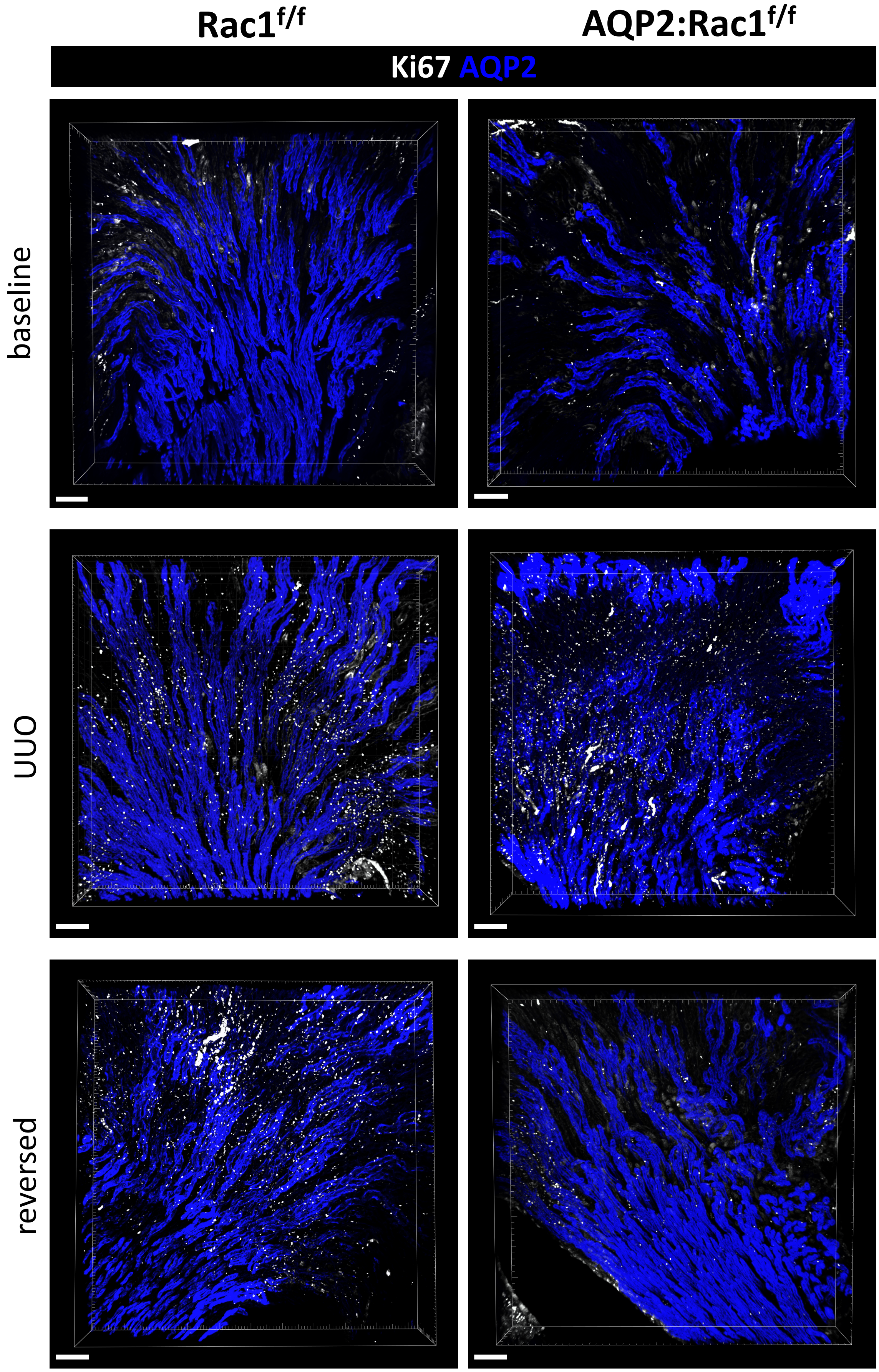

Supplemental Figure 9. Original image stacks corresponding to main Figure 3A. Scale bar 100 µm.

Supplemental Figure 10:

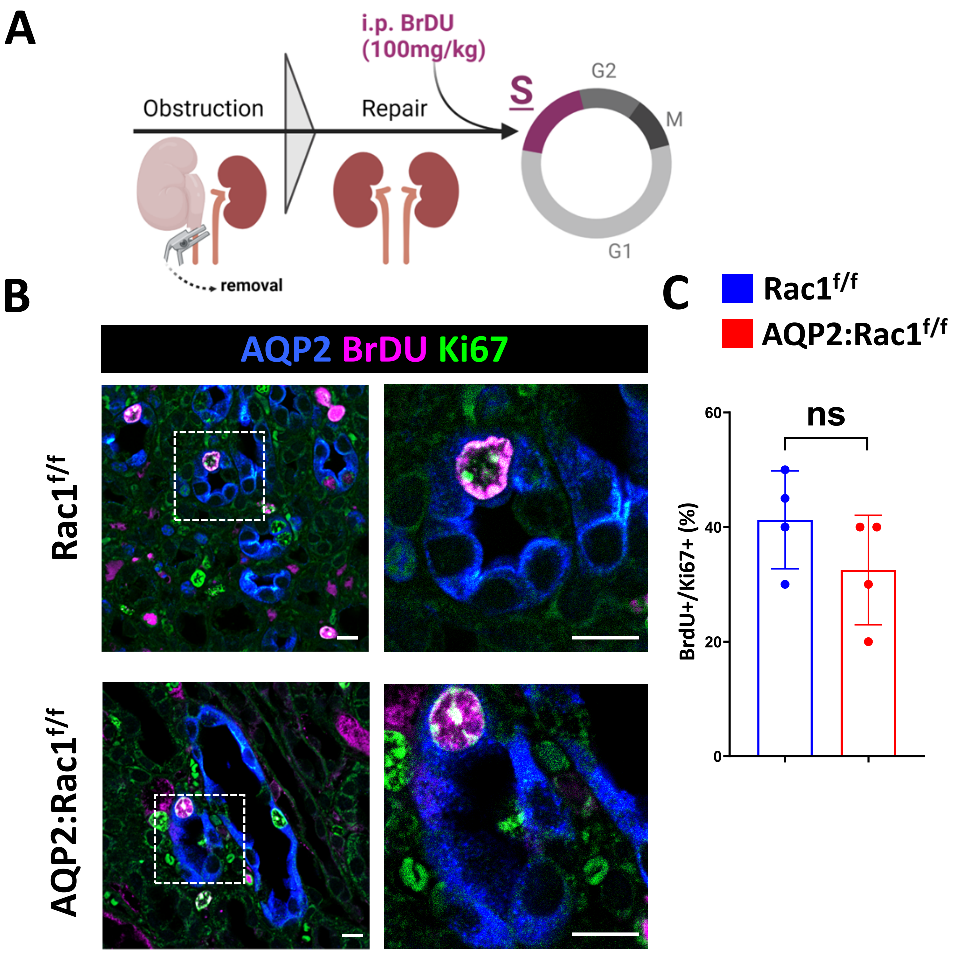

Supplemental Figure 10. (A) Schematic presentation of the experimental setup to assess S-phase entry *in vivo* (BrDU incorporation) using an intraperitoneal BrdU pulse. Created with Biorender.com. **(B,C)** Immunofluorescence of BrdU (magenta), Ki67 (green), and AQP2 (blue) to mark CDs of reversed control and AQP2:Rac1$^{f/f}$ kidneys with a quantification of BrDU positive proliferating (Ki-67) CD cells in percent with each dot representing individual mice. N=4 mice/group. Scale bar 10 µm. Bars are ± SD, ns: not significant.

Supplemental Figure 11:

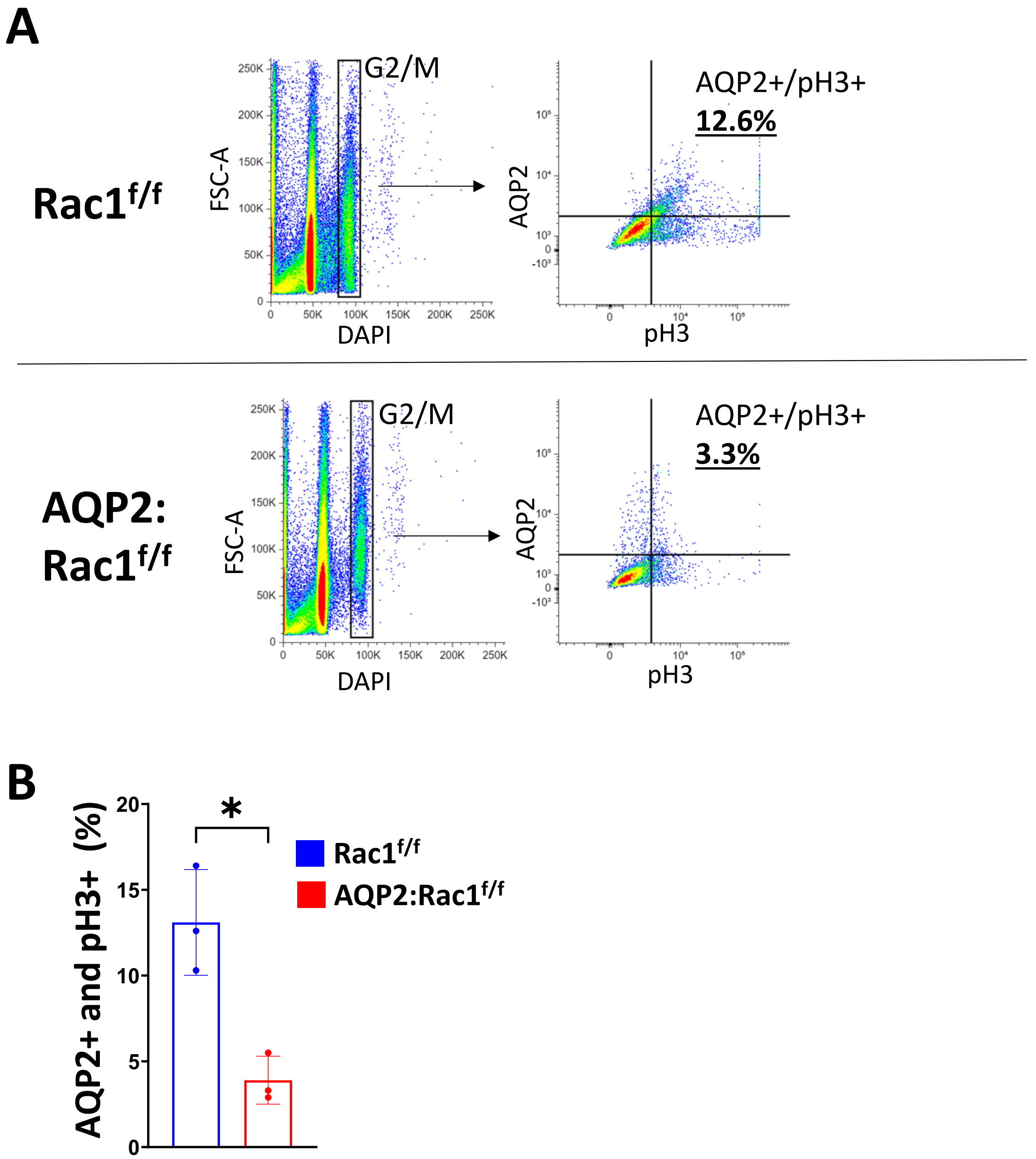

Supplemental Figure 11. (A,B) Assessment of AQP2 and pH3 double positive cells in G2/M during repair of reversed control and AQP2:Rac1$^{f/f}$ mice using flow cytometry. The first plot shows forward scatter (FSC-A) against DNA (DAPI) and a general G2/M gate. The second plot shows AQP2 signal against pH3 signal with double positive cell percentages shown in the top right quadrant. Three independent samples are quantified in **B** with dots representing individual mice. Bars are ± SD. $^*P < 0.05$.

Supplemental Figure 12:

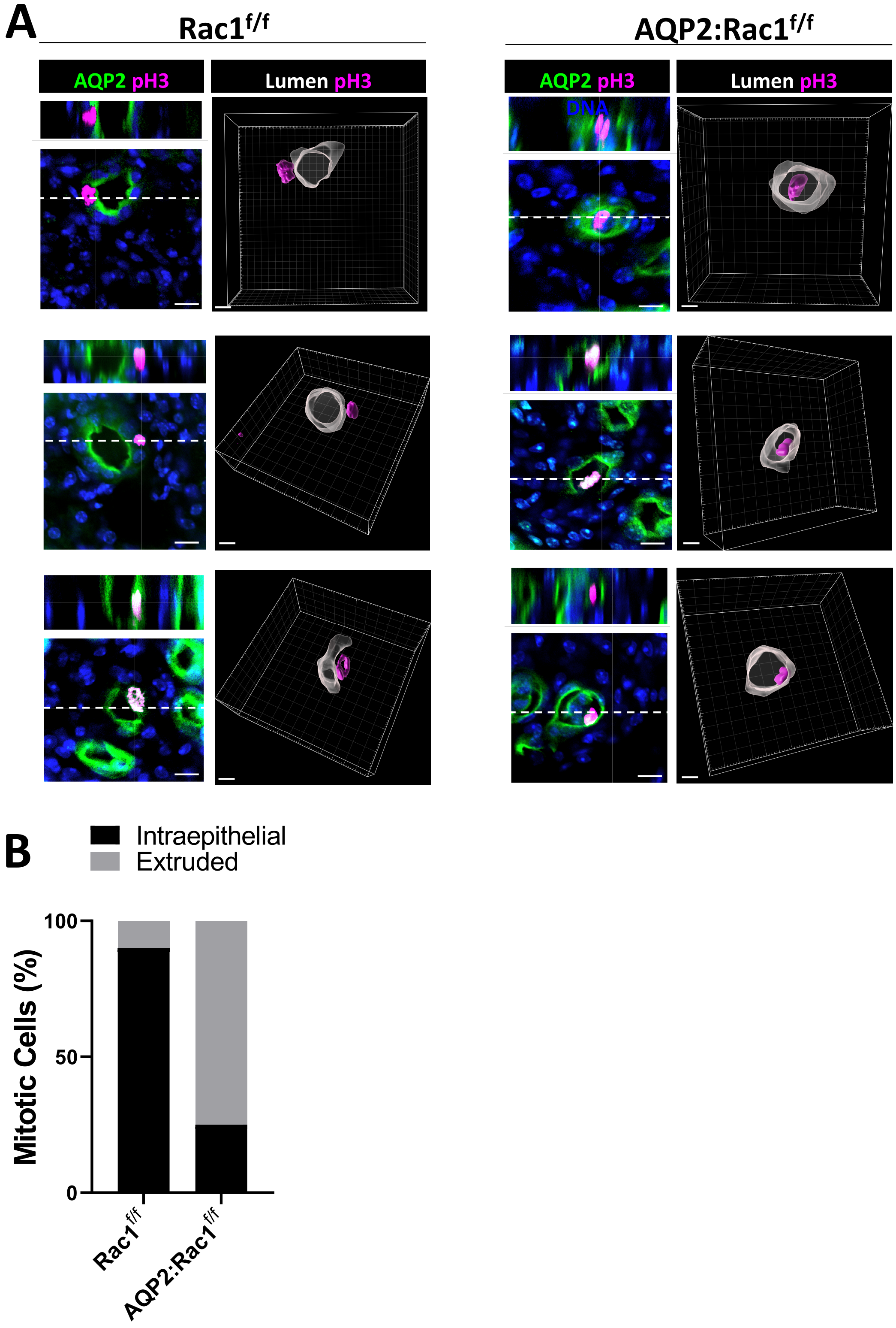

Supplemental Figure 12. (A,B) Optically cleared medullary slices of reversed control and AQP2:Rac1$^{f/f}$ mice were stained for mitotic cells (pH3, magenta) and CDs were labelled with AQP2 (green). The white dotted line indicates the orthogonal Z-slices shown at the top of the image. The right panel for each group shows 3D surface reconstructions using imaris of the intraluminal surface and the mitotic cell. Three representative image stacks are shown per group. Scale bars 10µm. The relative distribution of intraepithelial vs luminal mitotic figures is quantified in **B** with at least ten mitoses analyzed per group.

Supplemental Figure 13:

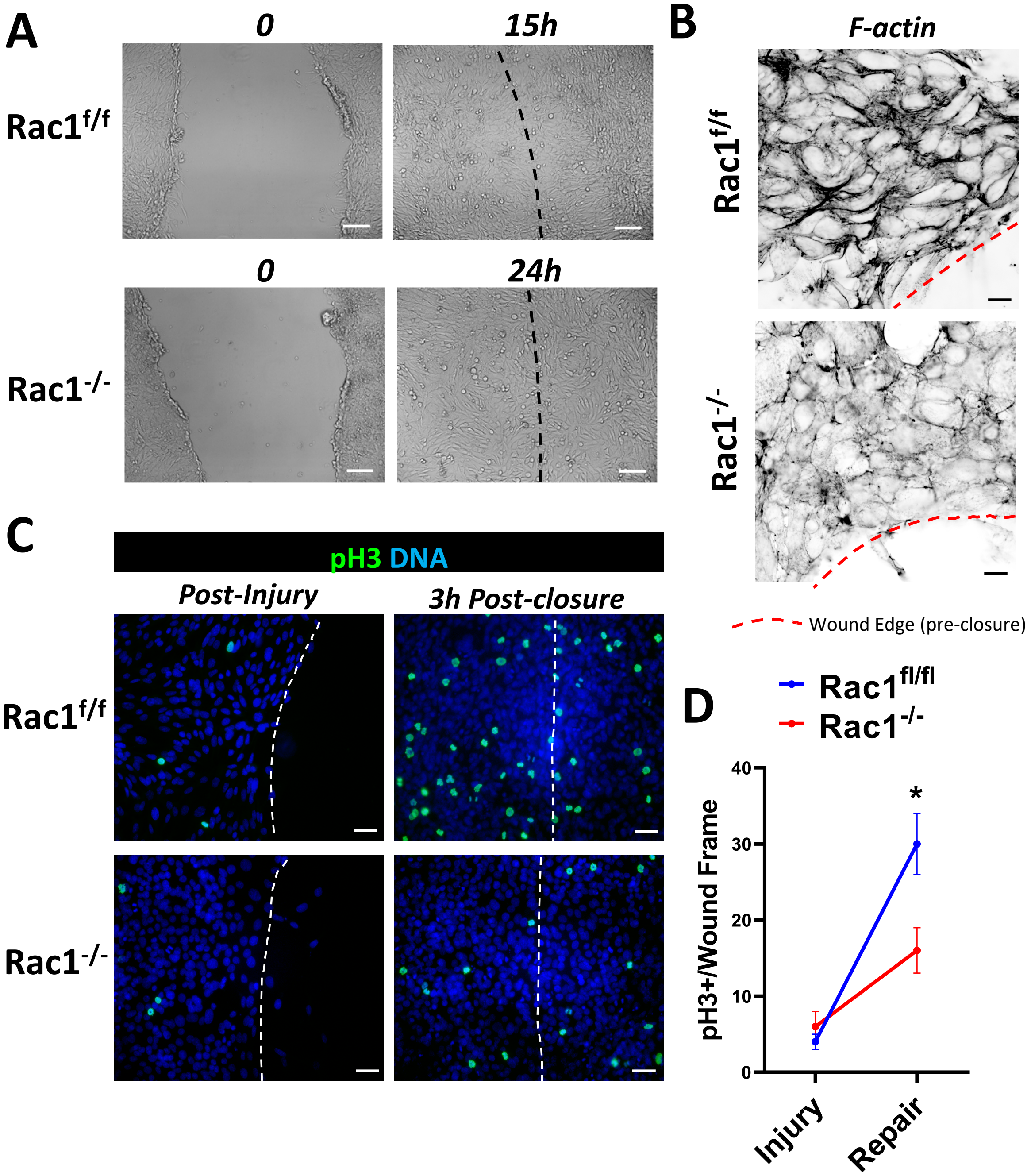

Supplemental Figure 13. (A) Confluent Rac1$^{f/f}$ and Rac1$^{-/-}$ CD cells were wounded (left panel) and allowed to migrate in full medium until cells had covered the scratch areas (right panel). This took ~15h in control cells and ~24h in Rac1 deficient CD cells. Scale bar 100 µm. Images are representative of at least three biological replicates. **(B)** Pre-wound closure Rac1$^{f/f}$ and Rac1$^{-/-}$ CD cell layers were labelled for F-actin (black) and analyzed by confocal microscopy with the migrating wound edge highlighted by a red dotted line. Scale bar 10 µm. **(C,D)** Post-scratch ("Post-Injury") and post-closure CD monolayers were stained for mitotic cells (pH3, green) and nuclei are highlighted with DAPI (blue). The Scratch is indicated by a white dotted line. Scale bar 20 µm. Post-wound closure mitotic cells per wound frame over time are quantified in D. Three independent repeat experiments. Bars are ± SD. *$P < 0.05$.

Supplemental Figure 14:

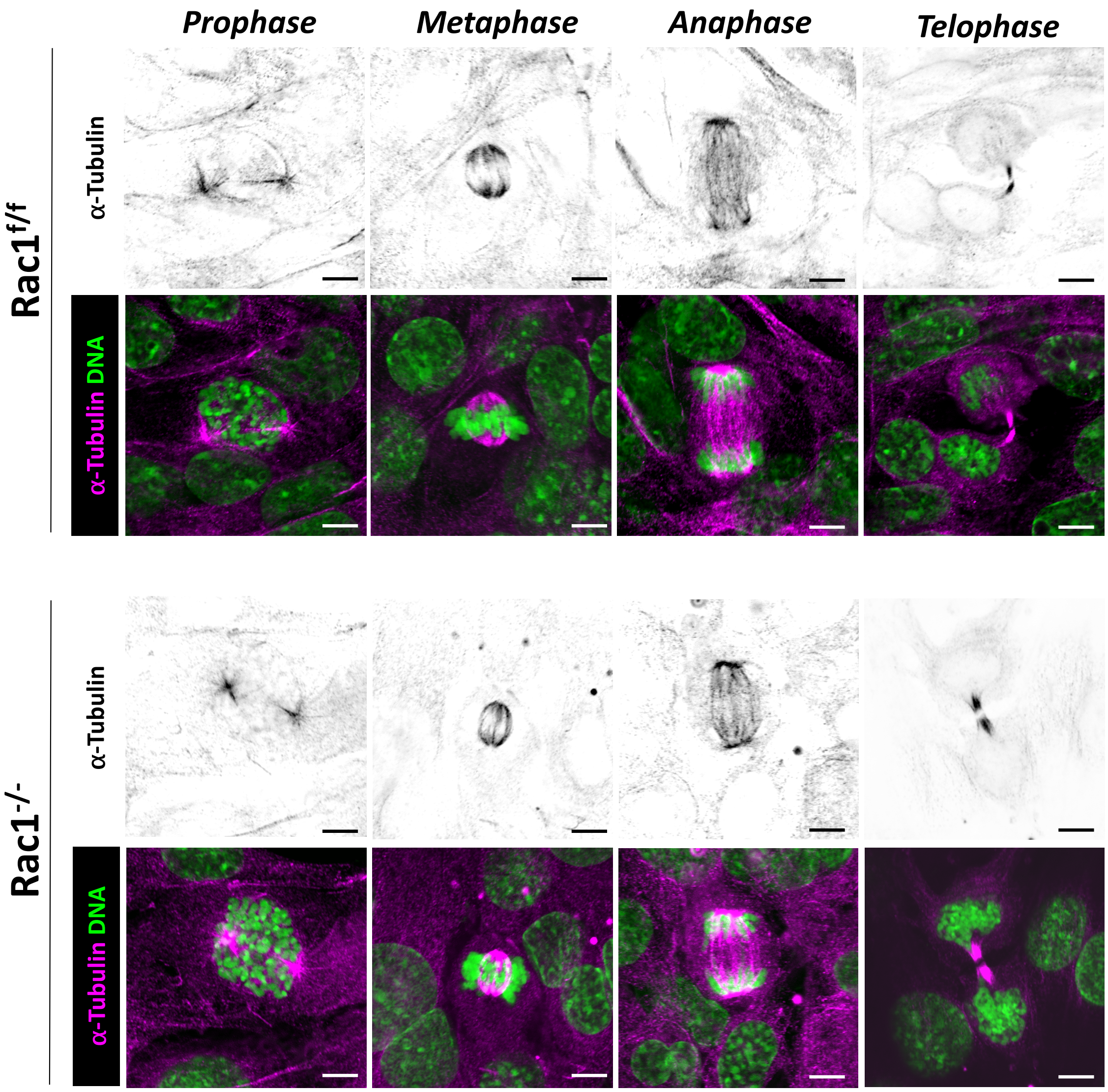

Supplemental Figure 14. Assessment of mitotic spindle formation using super-resolution confocal microscopy in Rac1$^{f/f}$ and Rac1$^{-/-}$ CD cell monolayers with α-tubulin (magenta) labeling microtubules and nuclei are labeled with DAPI (green). Each column shows a mitotic substage (Prophase, Metaphase, Anaphase, Telophase) with distinct spindle morphology that is largely preserved in both groups. Top row shows the α-tubulin channel (in gray with LUT-inverted). Representative of three repeat experiment. Scale bars 5 μm.

Supplemental Figure 15:

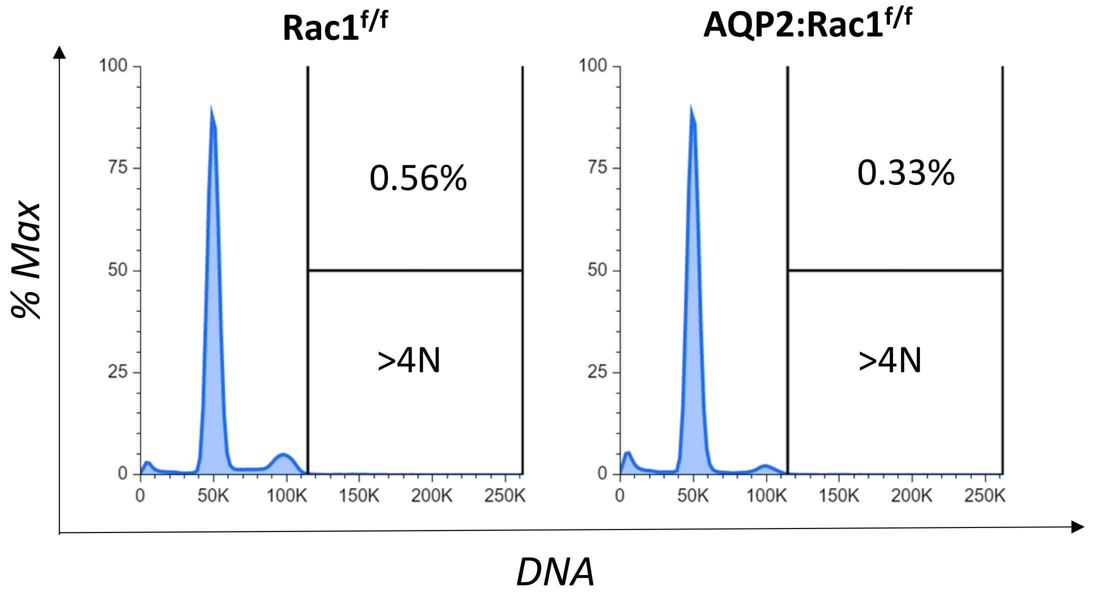

Supplemental Figure 15. Aneuploidy (>4N) assessment *in vivo* by flow cytometry of AQP2+ cells demonstrating low aneuploidy rates in reversed control and AQP2:Rac1$^{f/f}$ kidneys. Flow cytometry histograms show normalized cell counts (mode) against DNA content with aneuploid cell percentages indicated in the plot. The plots are representative of at least three mice per group.

Supplemental Figure 16:

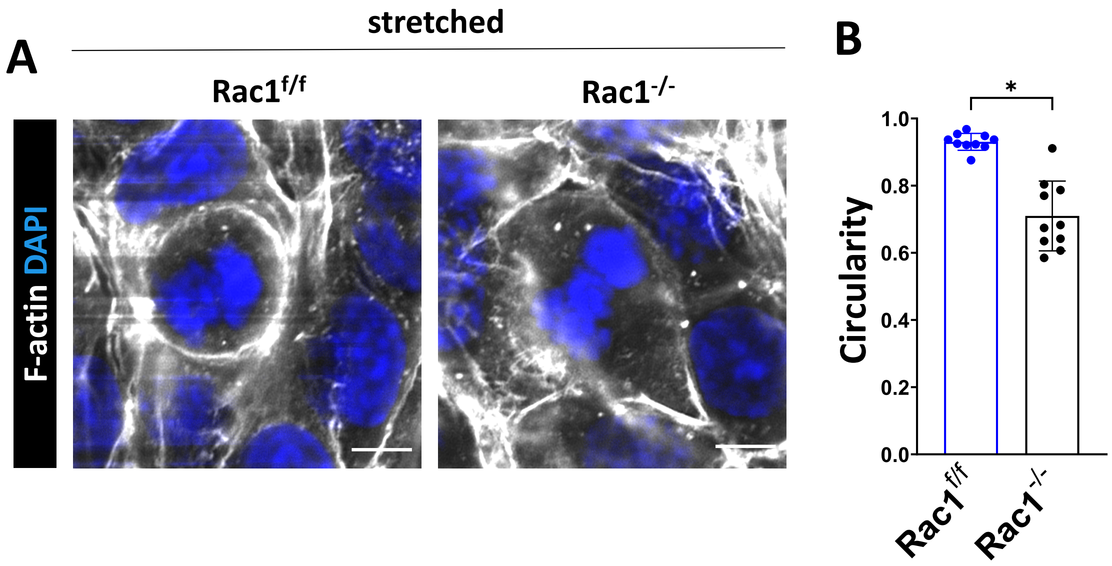

Supplemental Figure 16. (A,B) F-actin (white) and DNA (blue) labeled Rac1$^{f/f}$ and Rac1$^{-/-}$ CD cells after release of stretch ("stretched"). Scale bar 5 µm. Metaphase F-actin circularity is quantified in **B** with at least 10 measurements per group shown. Bars are ± SD. *P < 0.05.

Supplemental Figure 17:

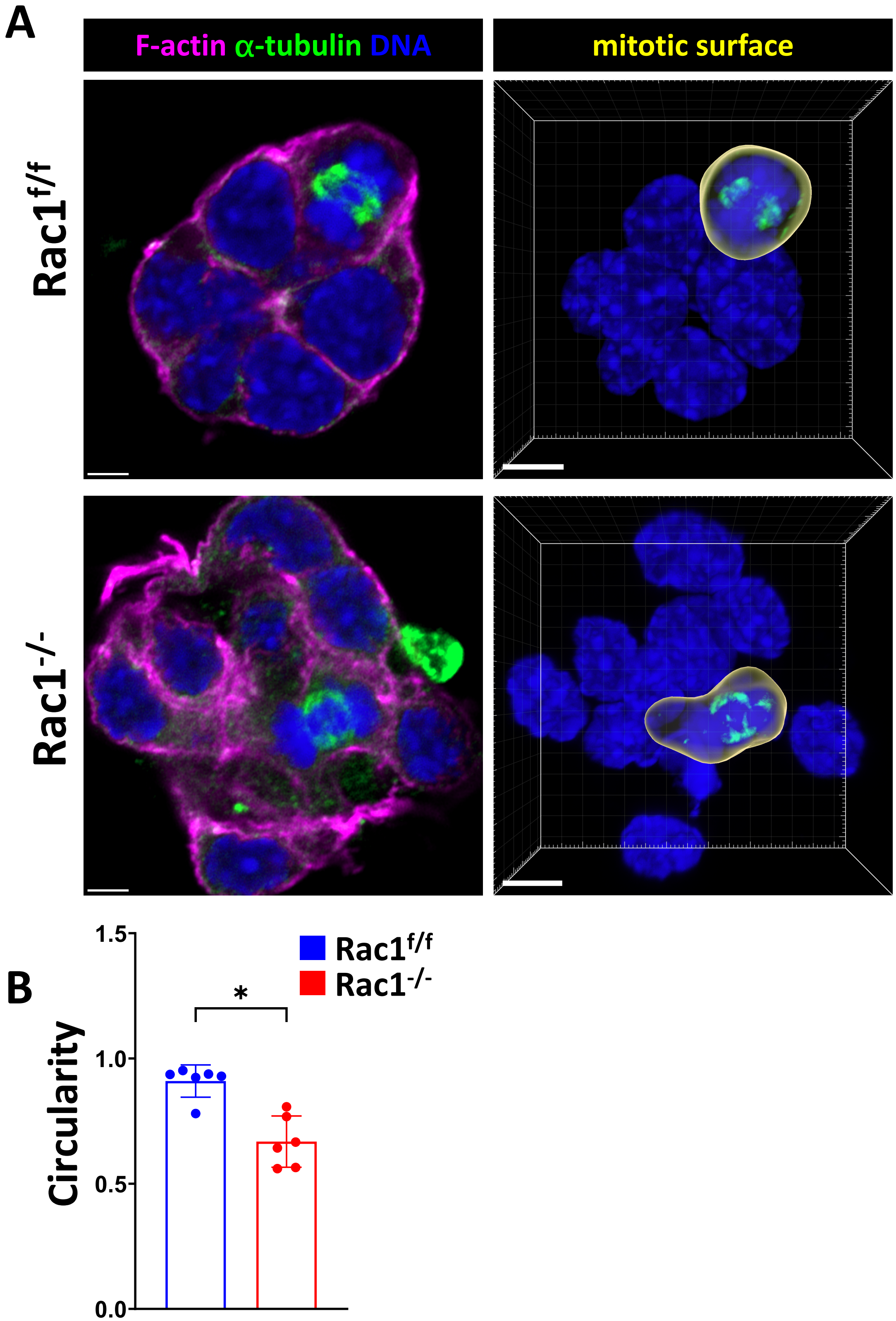

Supplemental Figure 17. (A,B) F-actin (magenta), DNA (blue) and α-tubulin (green) labeled Rac1$^{f/f}$ and Rac1$^{-/-}$ CD spheroids grown in 3D matrix and analyzed by confocal microscopy. The column on the right displays 3D surface reconstructions of mitotic metaphase F-actin (yellow surface) with DAPI and an α-tubulin mask. Scale bars 3µm left column and 5µm right column. Metaphase F-actin circularity is quantified in **B** with at least six measurements per group shown. Bars are ± SD. *P < 0.05.

Supplemental Figure 18:

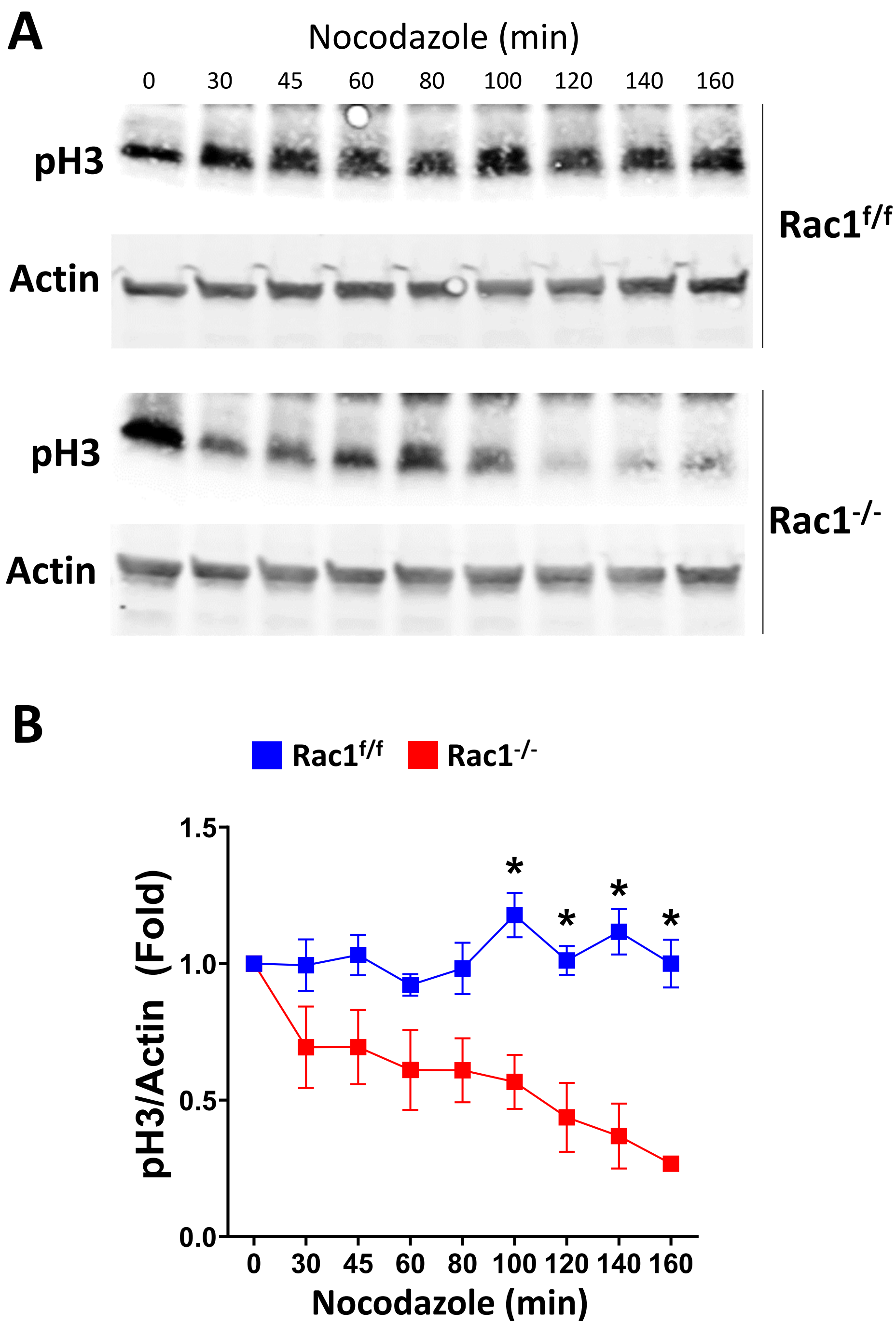

Supplemental Figure 18. (A,B) $Rac1^{f/f}$ and $Rac1^{-/-}$ CD cells were metaphase-arrested using nocodazole (100ng/ml). Lysates were obtained at the indicated time points and immunoblotted for pH3 to monitor mitosis. Two repeat experiments were quantified using densitometry in **B** shown as the mean fold change ± SD. *P < 0.05.

Supplemental Figure 19:

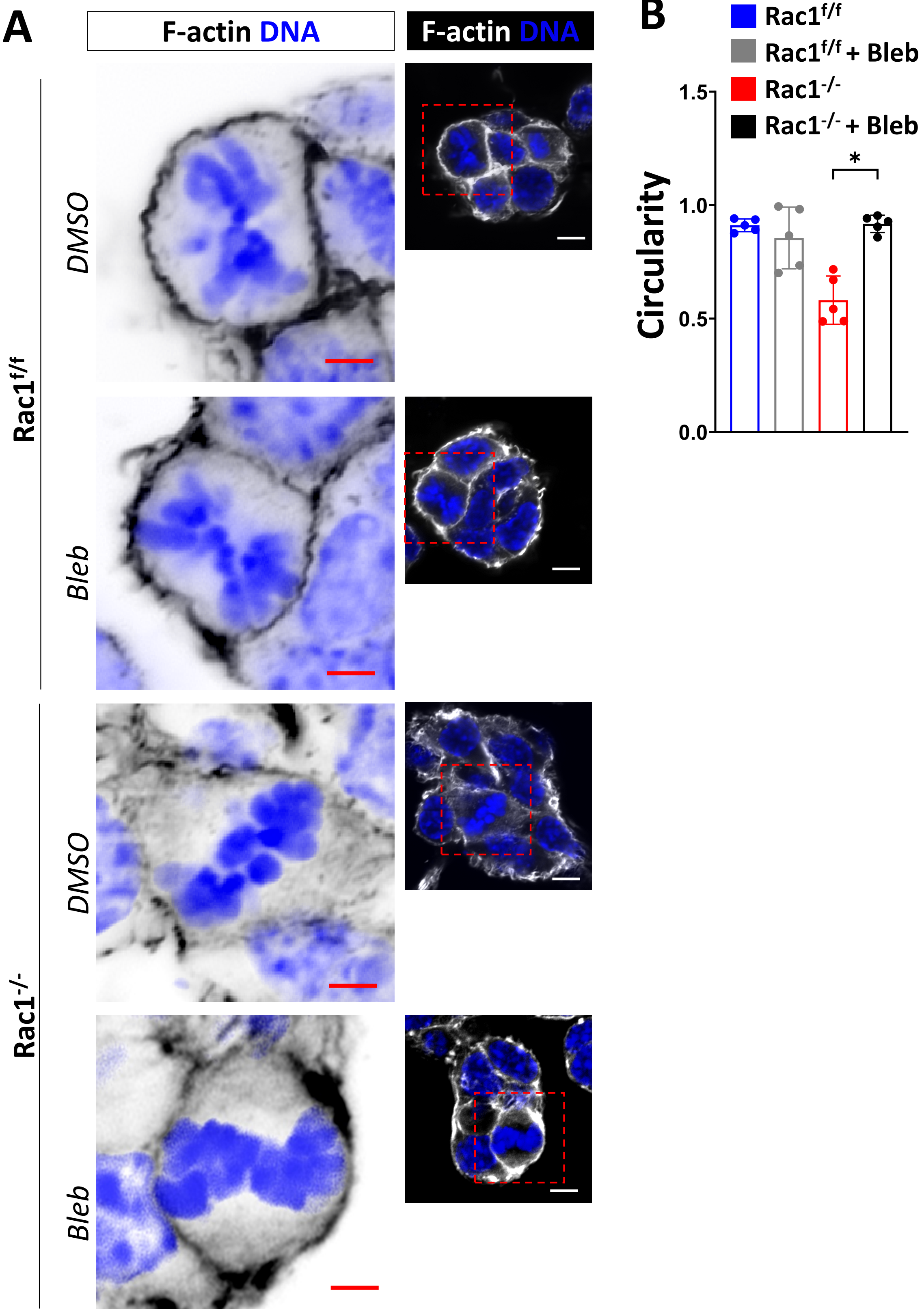

Supplemental Figure 19. (A,B) F-actin (white) and DNA (blue) labeled Rac1$^{f/f}$ and Rac1$^{-/-}$ CD spheroids (treated with vehicle, DMSO, or blebbistatin, Bleb 5μM) grown in 3D matrix and analyzed by confocal microscopy. The left panel shows color-inverted insets of mitotic metaphase F-actin (Scale bar 2.5 μm) as outlined by a red dotted box in the original images in the right panel (Scale bar 5 μm). Metaphase F-actin circularity is quantified in **B** with at least five measurements per group shown. Bars are ± SD. *$P < 0.05$.

Supplemental Figure 20:

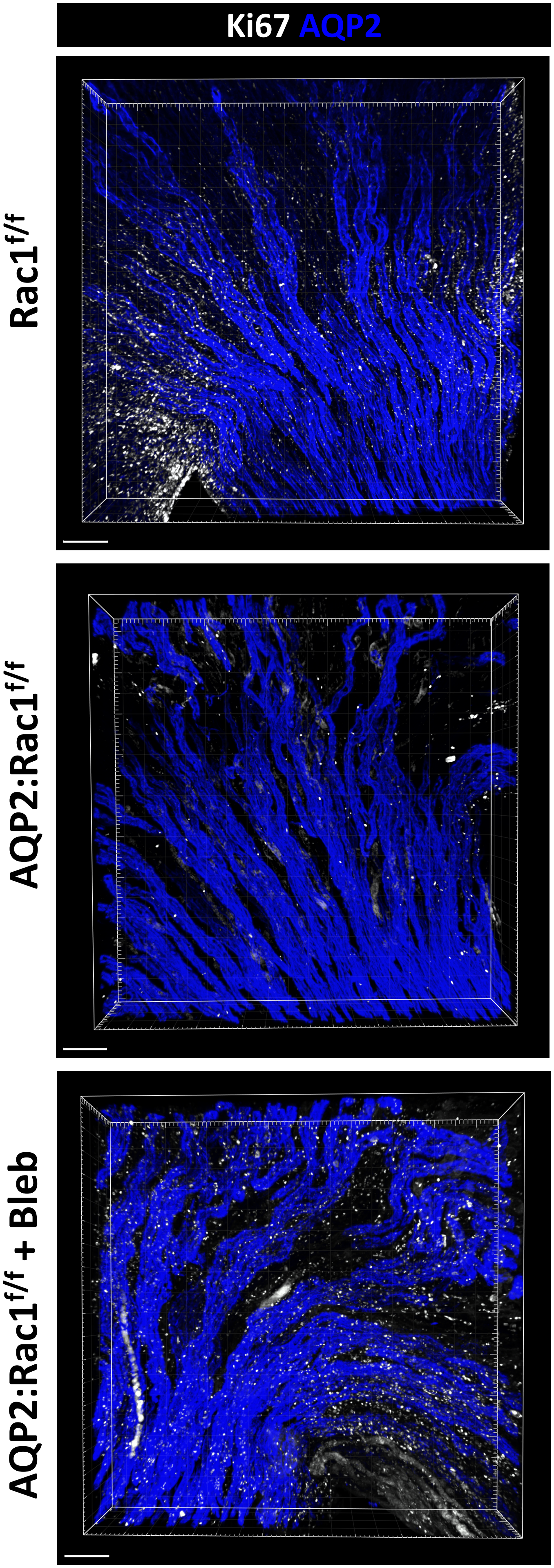

Supplemental Figure 20. Original image stacks corresponding to main Figure 7A. Scale bar 100 μm.

Supplemental Figure 21:

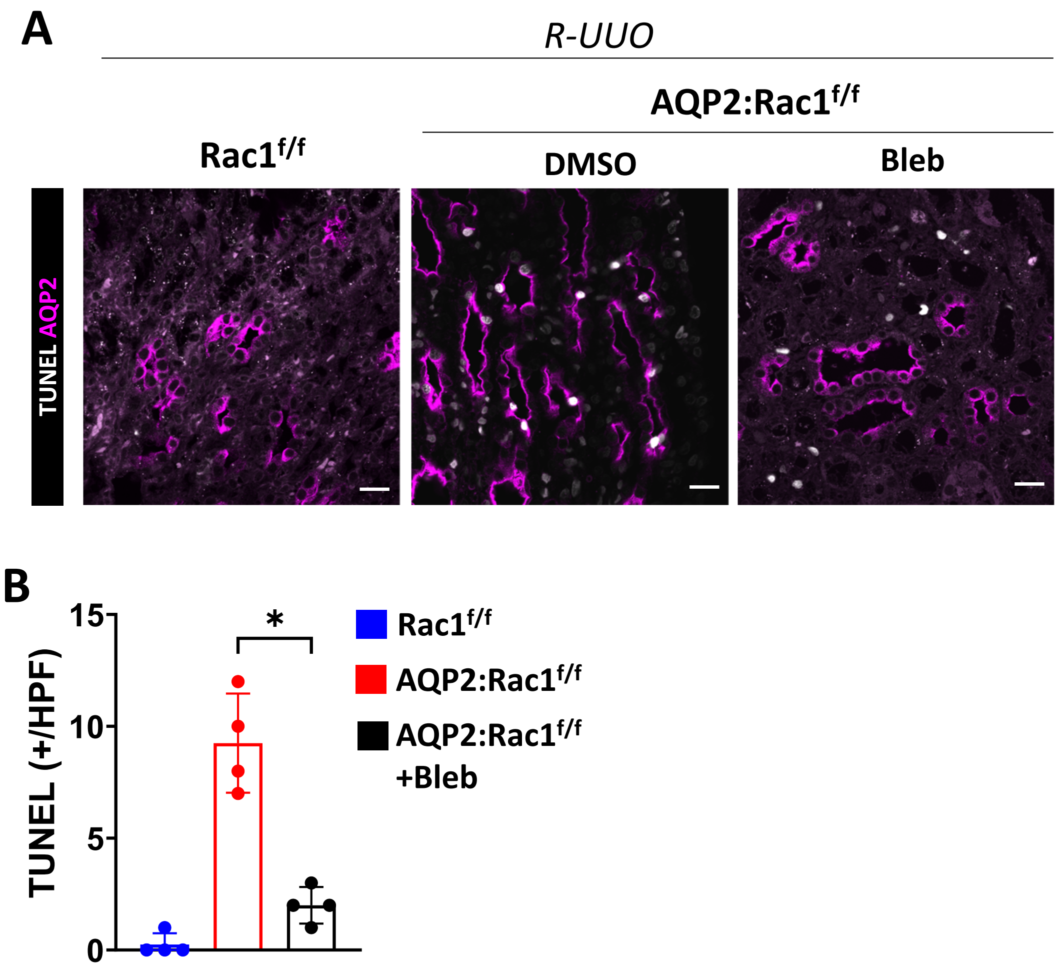

Supplemental Figure 21. (A,B) Apoptosis (TUNEL, white) labeling of CDs (AQP2+, magenta) of paraffin kidney sections in reversed $Rac1^{f/f}$ and vehicle or blebbistatin treated AQP2:$Rac1^{f/f}$ mice. (B) Quantification of TUNEL positive cells per medullary HPF with each dot representing individual samples. N=4 mice per group. Bars are ± SD. *P < 0.05.

Supplemental Figure 22:

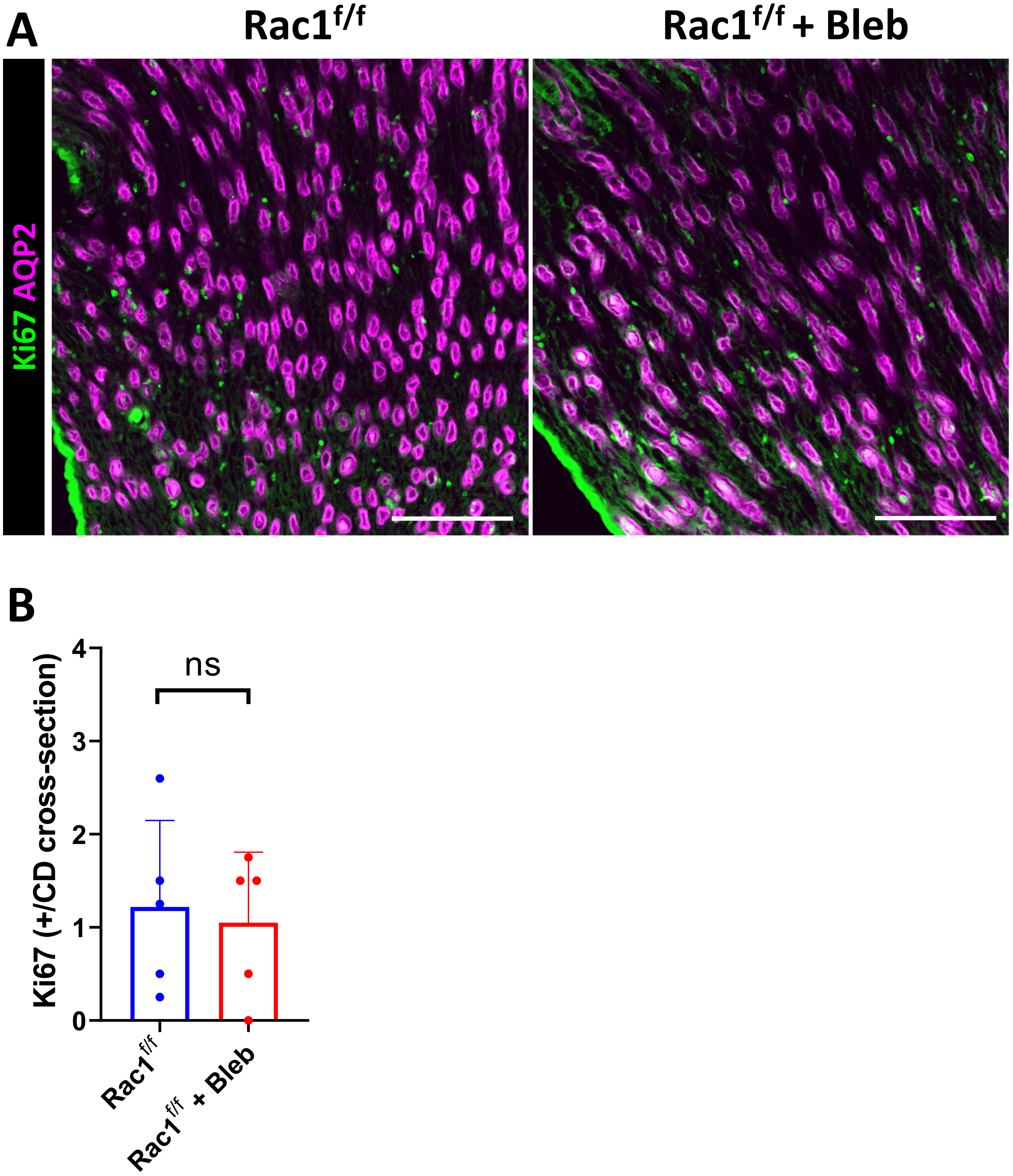

Supplemental Figure 22. (A) Assessment of proliferation (Ki-67, green) of vehicle or blebbistatin-treated control (Rac1$^{f/f}$) mice at baseline. A medullary cross-section is shown with the collecting ducts (CDs) marked by AQP2 (magenta). Scale bar 125µm. **(B)** Quantification of Ki-67 positive cells per collecting duct cross-section with dots representing individual mice. Bars are mean + SD. ns: not significant.

Supplemental Figure 23:

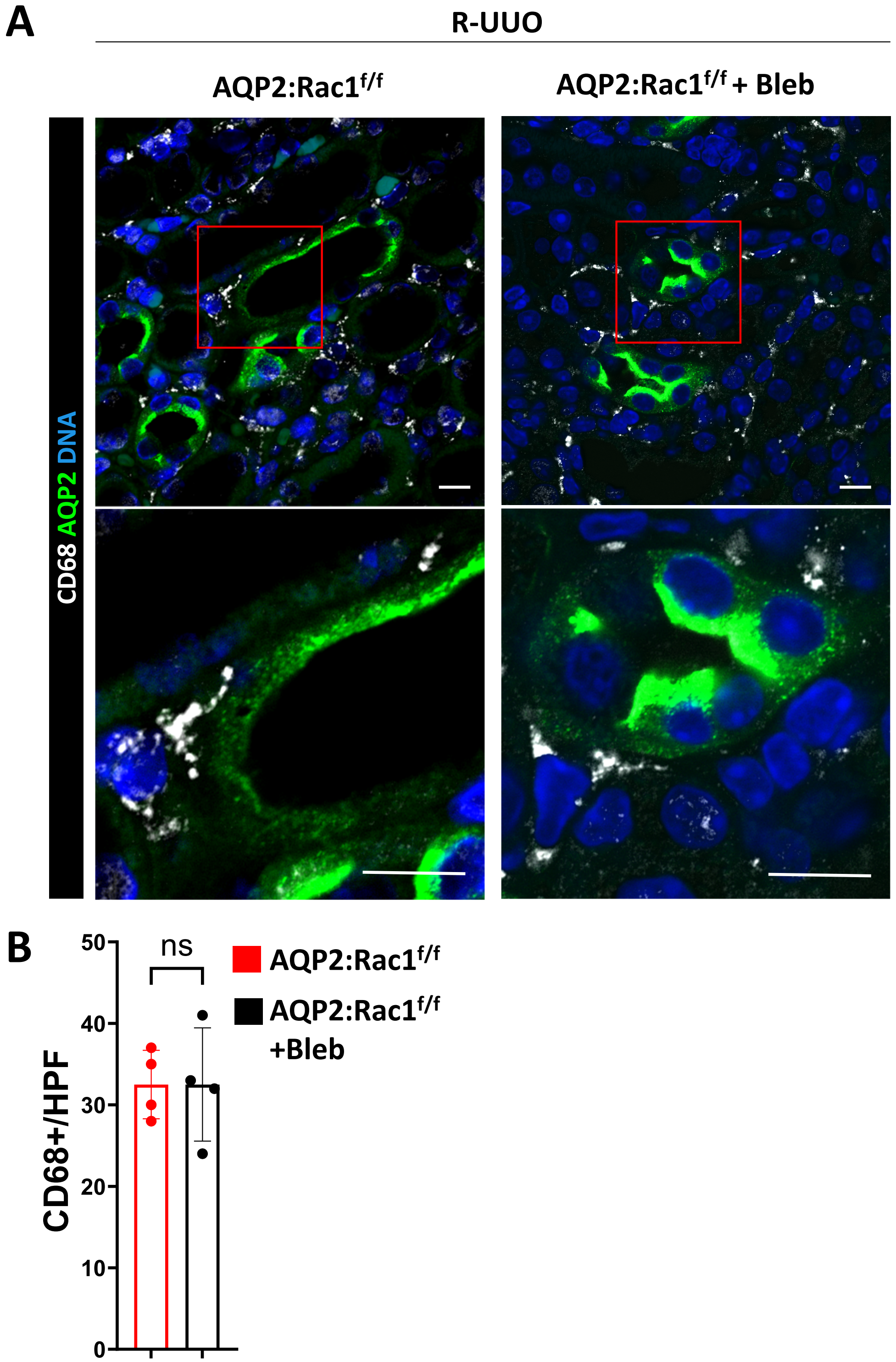

Supplemental Figure 23. (A) Assessment of CD68+ (white) immune cell infiltration (macrophages) around repairing vehicle or blebbistatin-treated AQP2:Rac1$^{f/f}$ CDs (AQP2, green). Nuclei: DAPI (blue). Scale bar 10 µm. **(B)** Quantification of CD68 positive cells per high power field (HPF) with dots representing individual mice. Bars are mean + SD. ns: not significant.

Supplemental Figure 24:

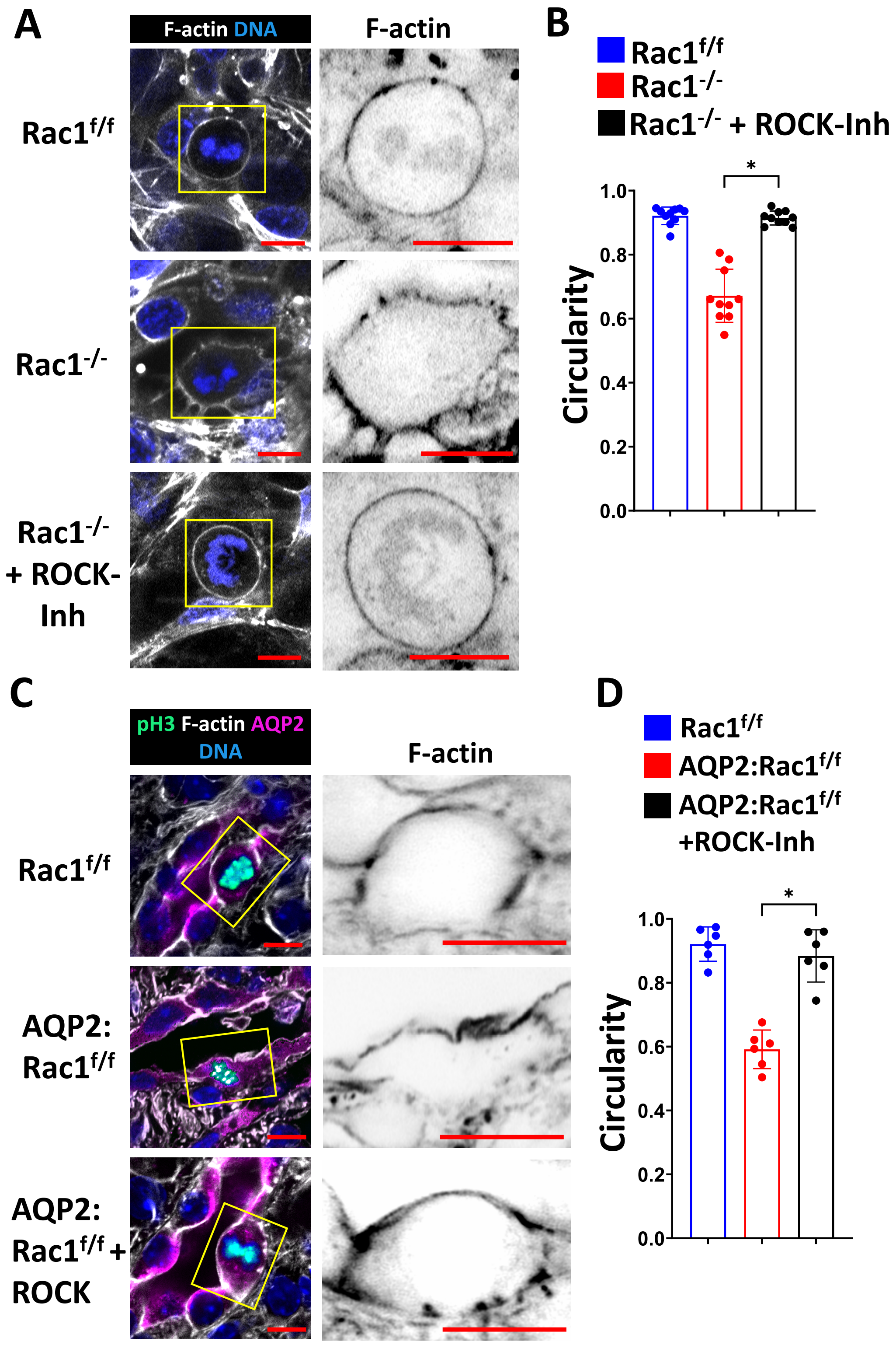

Supplemental Figure 24. (A,B) F-actin (white) and DNA (blue) labeled Rac1$^{f/f}$,Rac1$^{-/-}$, or ROCK-Inhibitor (Y-27632, 10µM, 1h pre-treatment prior to mitotic imaging). The right column shows color-inverted insets of mitotic metaphase F-actin as outlined in the yellow box in the left column. Metaphase F-actin circularity is quantified in **B** with at least 10 measurements per group shown. **(C,D)** Super-resolution imaging of F-actin-labelled (white) CDs (AQP2, magenta) with the mitotic metaphase cell labeled with pH3 (green) of reversed UUO mice (ROCK-Inh: i.p. injected with Y-27632 during repair). The yellow box outlines the insets that are shown in the right column which depicts color-inverted metaphase F-actin. Metaphase F-actin circularity is quantified in **D** with n=6/group. All scale bars 10 µm. Bars are ± SD. *P < 0.05.

Supplemental Figure 25:

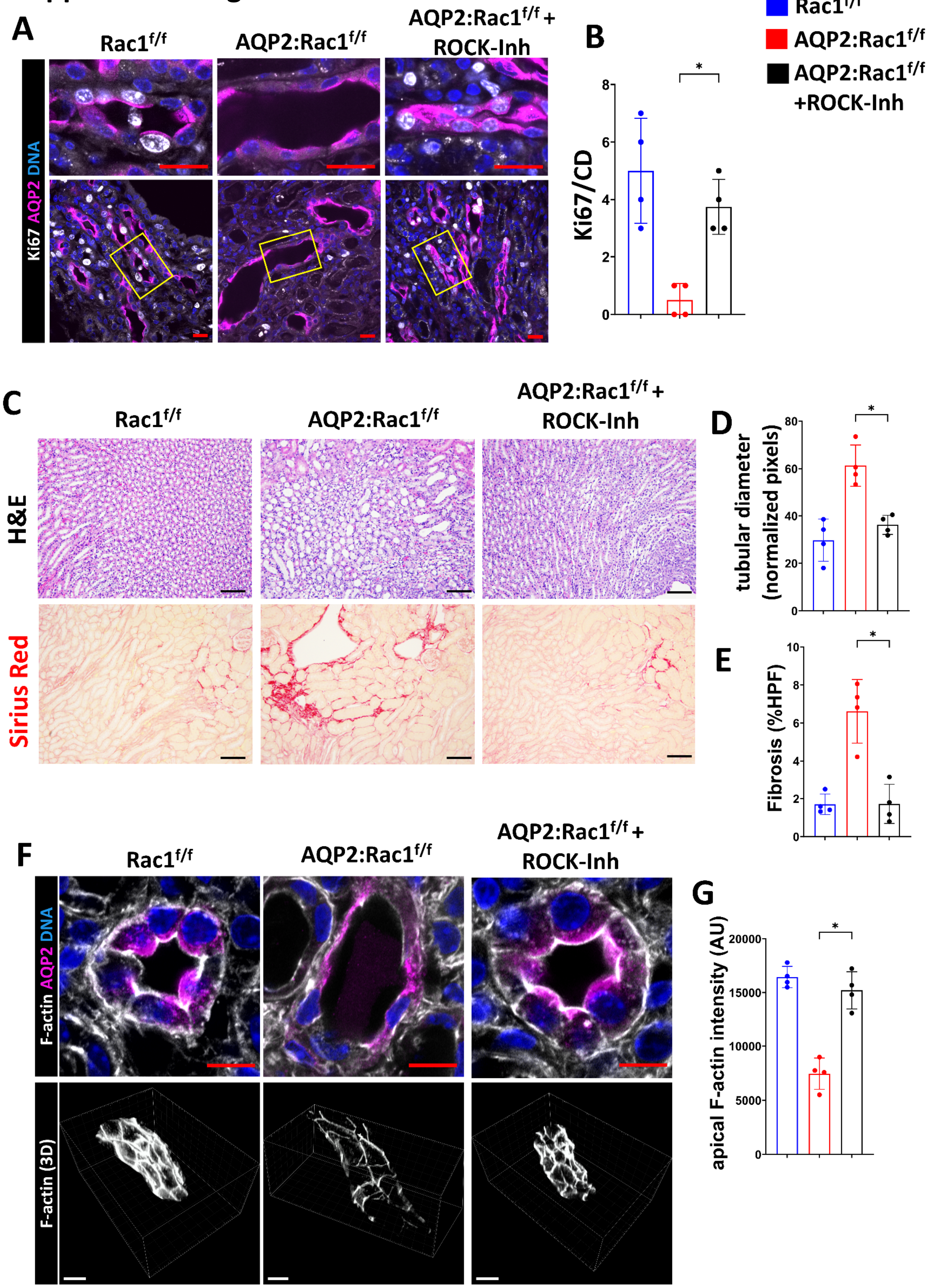

Supplemental Figure 25. (A,B) Assessment of proliferation (Ki-67, white) of repairing (R-UUO) control (Rac1$^{f/f}$) and untreated or ROCK-Inhibitor treated AQP2:Rac1$^{f/f}$ CDs (AQP2, magenta). Average Ki67 positive cells per CD per high power fields are shown in **B** with n=4 mice/group. The yellow box shows the region of the insets in the top row. Scale bar 20 µm. **(C-E)** H&E paraffin kidney sections in the first row (scale bar 50 µm) of medullary regions and Sirius red staining in the second row (scale bar 50 µm). Quantification of the average medullary tubular diameter and fibrosis in D and E with dots representing individual mice (n=4). **(F,G)** Cross-sections of F-actin labeled (white) CDs (AQP2, magenta) during repair (first row) and 3D confocal reconstructions of the apical F-actin cytoskeleton (second row). Scale bar 10 µm.) Quantification of the average apical F-actin intensity in G with dots representing individual samples (n=4). Bars are mean ± SD. *P < 0.05.

Supplemental Figure 26:

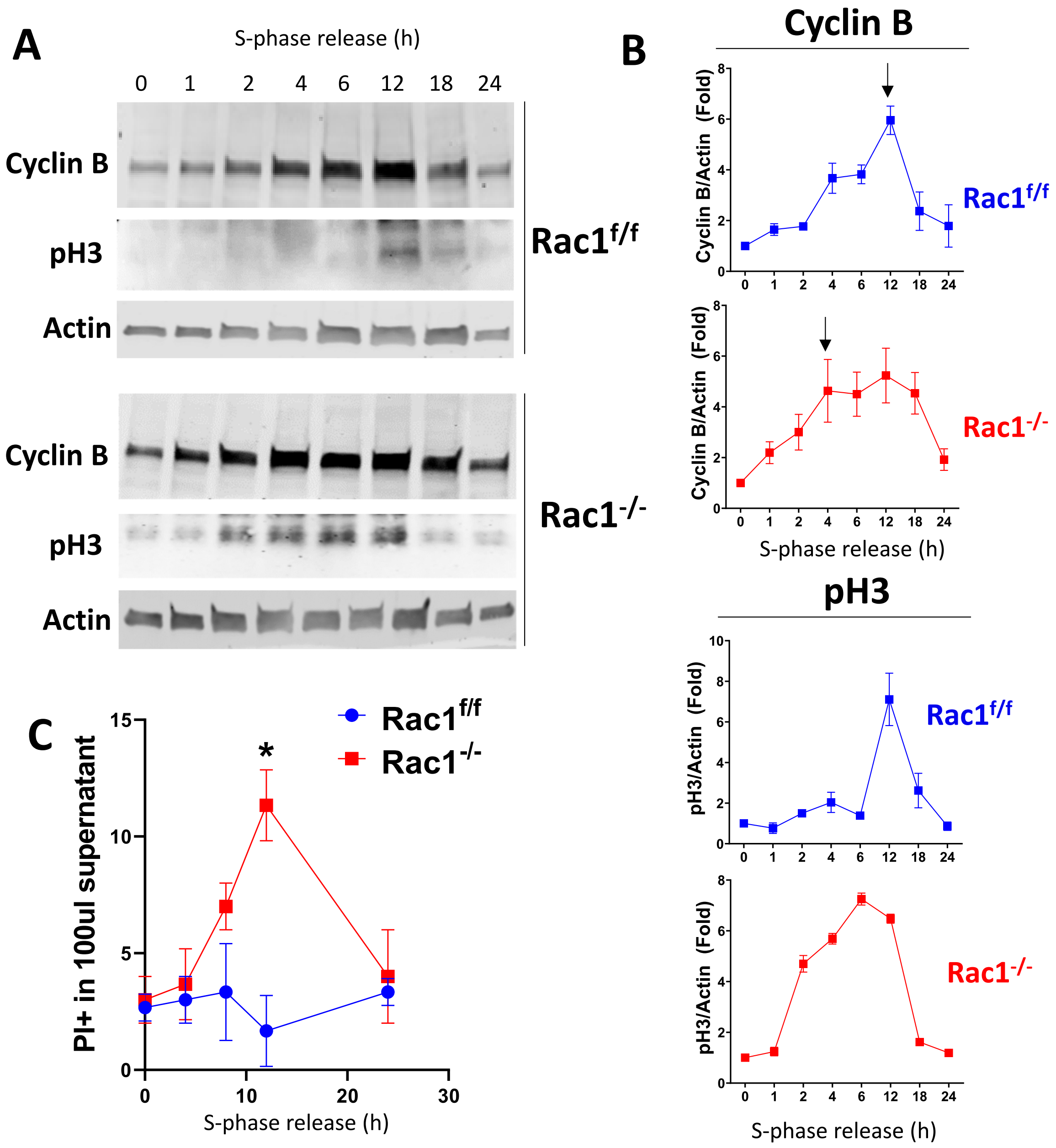

Supplemental Figure 26. (A,B) Rac1$^{f/f}$ and Rac1$^{-/-}$ CD cells were synchronized at the G1/S border using a double thymidine block. Lysate were collected at the indicated time points after S-phase release and immunoblotted for cyclin B1 and phosphorylated Histone H3 (pH3) to monitor mitotic entry. Three repeat experiments were quantified using densitometry in **B** and shown as mean fold change values ± SD. Arrows highlight the first pH3 peak of the respective groups indicating mitotic entry. **(C)** Propidium iodide staining and cell counting at the indicated time points of the supernatant after S-phase release. Three repeat experiments were quantified. Bars are ± SD. *P < 0.05.

Supplemental Figure 27:

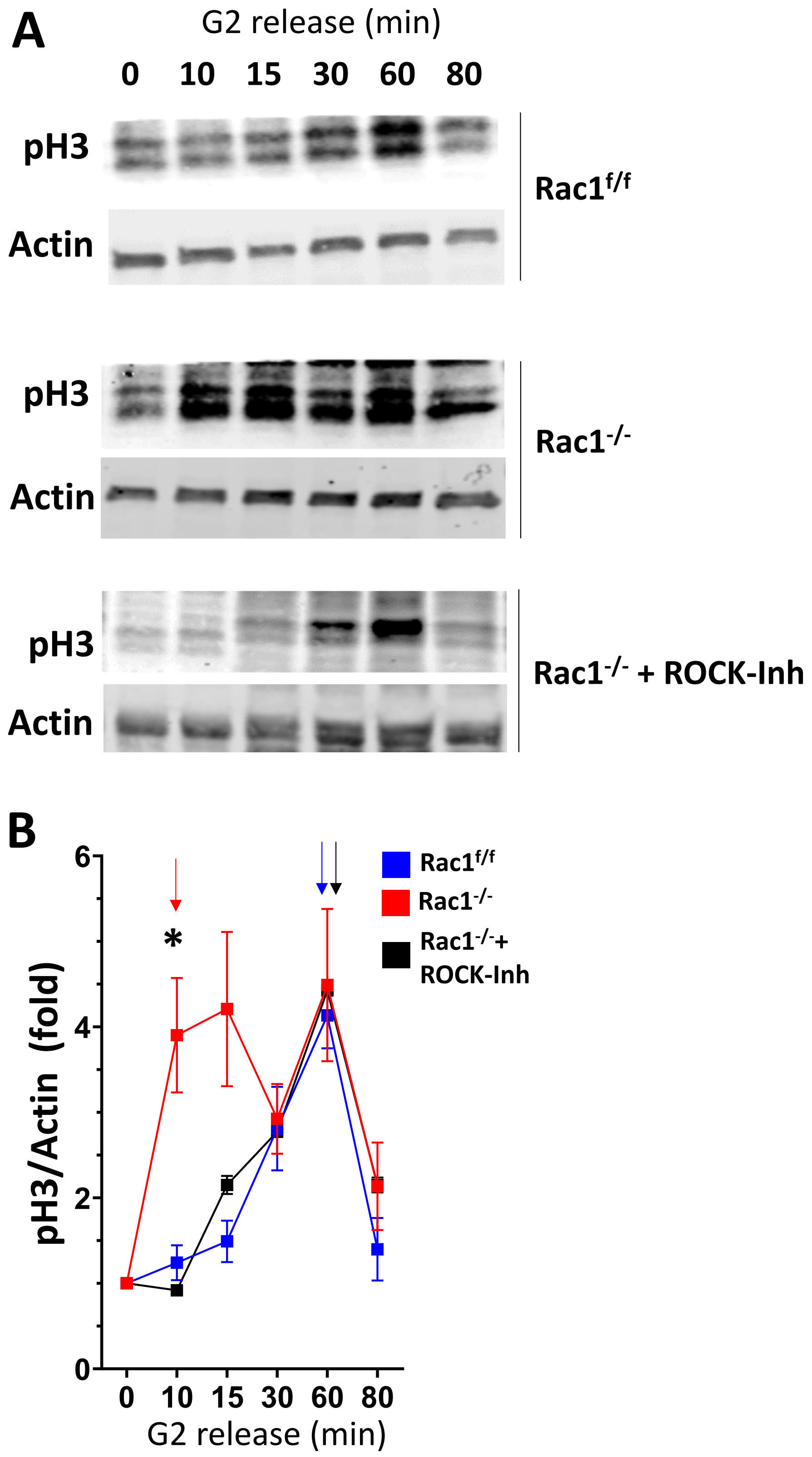

Supplemental Figure 27. (A,B) G2-synchronized Rac1$^{f/f}$ and untreated or ROCK-Inhibitor-treated (Y-27632, 10µM, treatment initiated at G2 release upon RO-3306 washout) Rac1$^{-/-}$ CD cells were immunoblotted for pH3 to monitor mitotic entry. Three independent repeat experiments are quantified in **B** as mean fold change values ± SEM. *denotes significance between Rac1$^{-/-}$ and ROCK-Inh treated Rac1$^{-/-}$ CD cells. Arrows indicate the first mitotic peak. *$P < 0.05$.

Supplemental Figure 28:

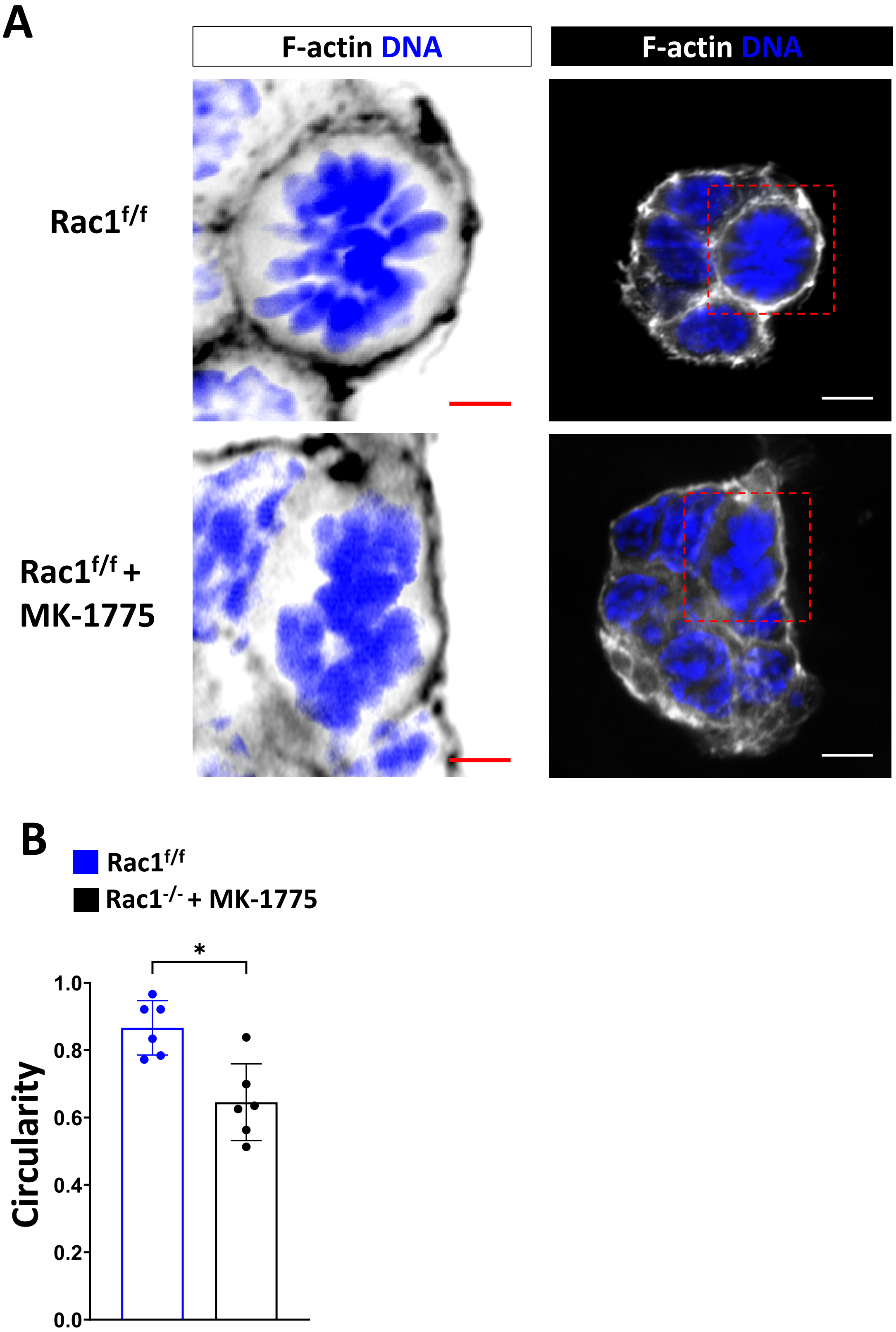

Supplemental Figure 28. Rac1$^{f/f}$ and Wee1-inhibited (MK-1775,1 µM) Rac1$^{f/f}$ CD spheroids grown in 3D matrix and analyzed by confocal microscopy. Scale bars 5 µm, Insets 2.5 µm. Metaphase F-actin circularity is quantified in **B** with at least six measurements per group shown. Bars are ± SD. *P < 0.05.

Supplemental Figure 29:

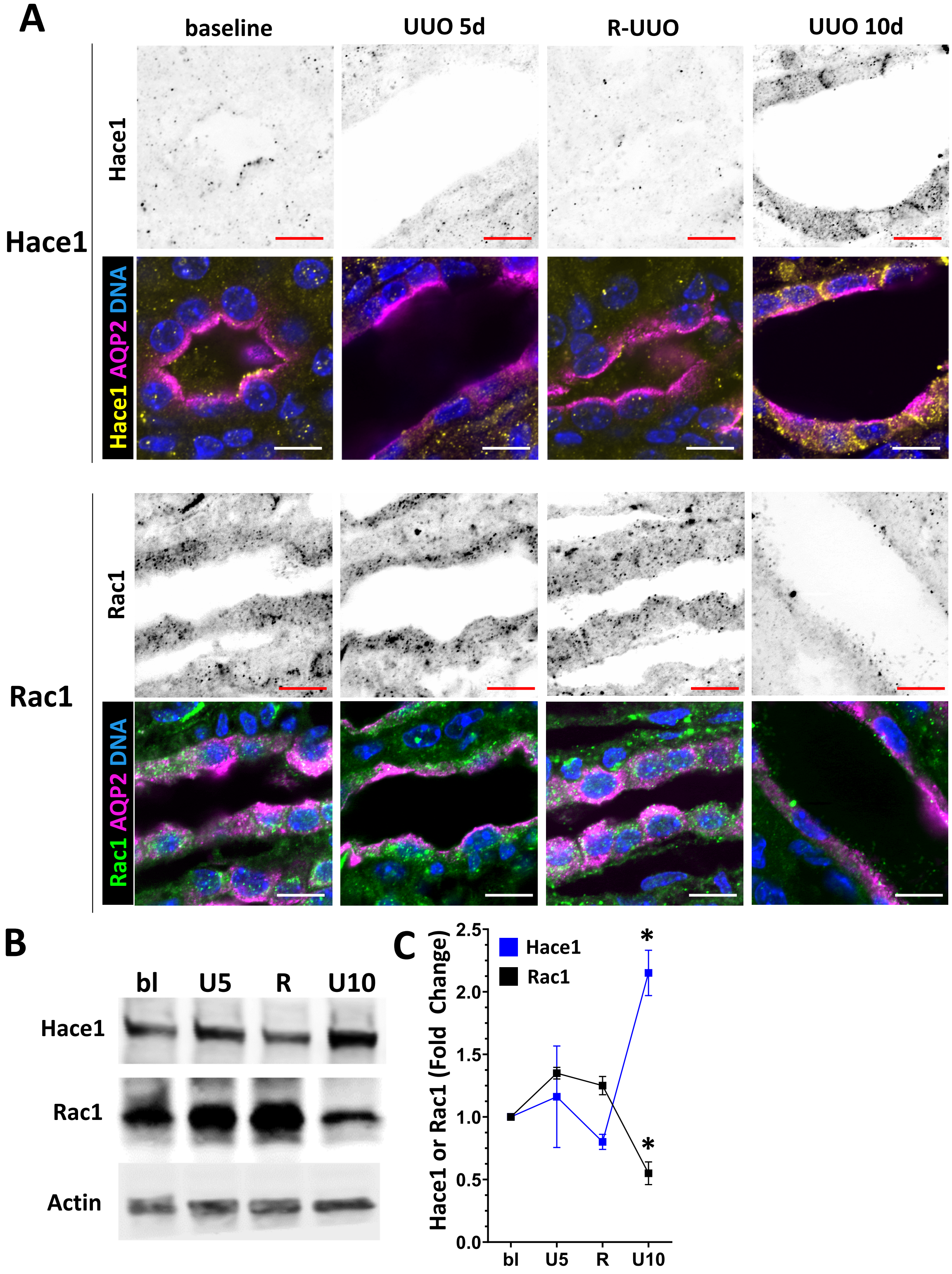

Supplemental Figure 29. (A) Immunostaining of baseline, UUO 5 days, reversed UUO (R-UUO), and UUO 10 days wild type collecting ducts (AQP2, magenta) for Hace1 (yellow) or Rac1 (green). DNA (DAPI) in blue. The top row represents the greyscale color-inverted Hace1 or Rac1 channel. Three mice per group and time point. Scale bar 10 µm. (**B,C**) Immunoblotting of medulla/papilla wild type lysates for Hace1 or Rac1 of the indicated groups (bl: baseline, U5: UUO 5 days, R: R-UUO, U10, UUO 10 days). Quantification of immunoblot results in **C** with three mice per group. *denotes significance for R vs U10. Mean ± SD. *P < 0.05.

Supplemental Figure 30:

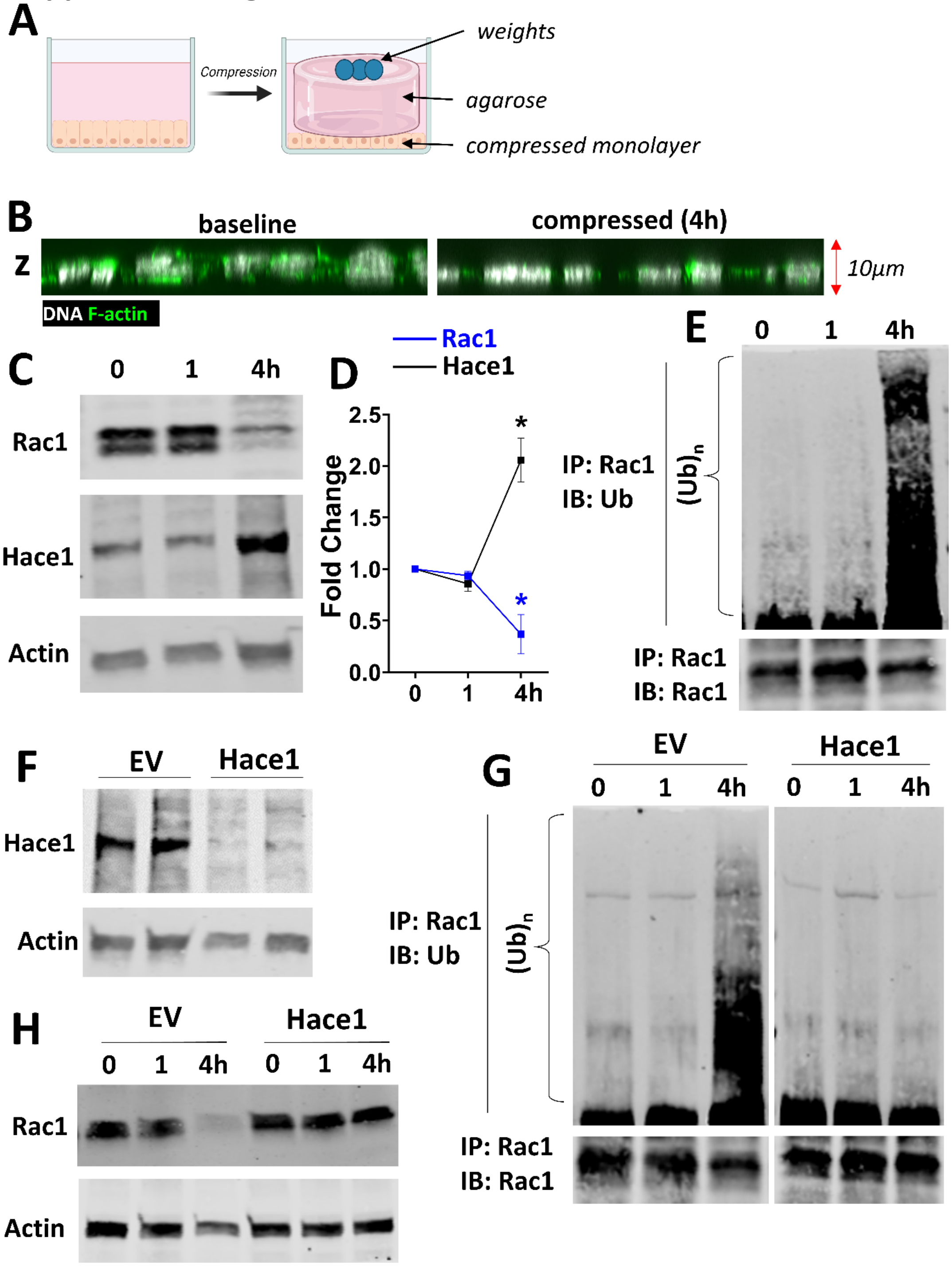

Supplemental Figure 30. **(A,B)** Schematic representation of the cell compression system (Created with Biorender.com) and 10 µm-thick orthogonal Z-stacks of baseline and compressed CD cells. **(C,D)** CD cells were compressed and immunoblotted for Rac1 and Hace1 at the indicated timepoints. Three repeat experiments are quantified in **C**. Bars are ± SD. *P < 0.05. **(E)** Compressed CD cells were immunoprecipitated (IP) with a Rac1 antibody and immunoblotted (IB) for Ubiquitin (UB) to assess polyubiquitination of Rac1 (Ub_n). **(F)** CD cells were transfected with Hace-1 shRNA and the knockdown was confirmed by immunoblotting for Hace1. Biological duplicates are shown. **(G)** The same experiment as **E** including EV (empty vector)- or Hace1-Knockdown transfected CD cells (Hace1). **(H)** Total EV- or Hace1-KD transfected CD cells were compressed and immunoblotted for Rac1. Immunoblots are representative of three experiments.

Supplemental Figure 31:

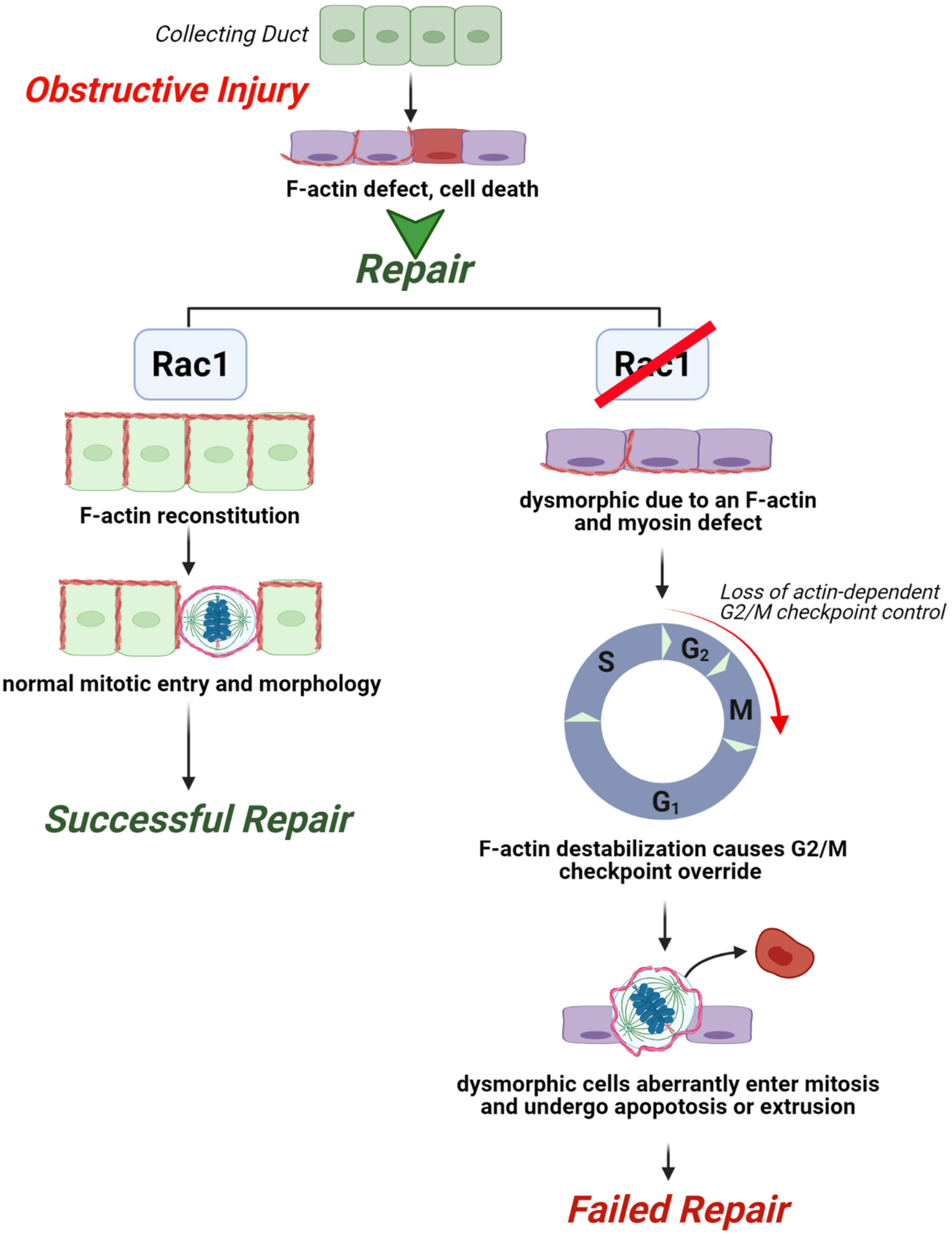

Supplemental Figure 31. Graphical Abstract. Rac1 promotes reconstitution of actin-dependent cell morphology after collecting duct injury which is required to maintain normal actomyosin activity. In the absence of Rac1, excessive actomyosin activity leads to myosin-dependent degradation of the negative mitotic entry regulator Wee1. This causes G2/M checkpoint instability in Rac1 deficient CD cells which results in premature mitotic entry of dysmorphic cells which mechanically fail mitosis and undergo extrusion or apoptosis. Rac1 thus promotes CD repair by coupling cell morphology to mitotic entry to prevent dysmorphic cells from aberrantly entering mitosis. Created with Biorender.com.

Supplemental Figure 32:

A *Exemplary quantification Figure 1D: tubular F-actin content*

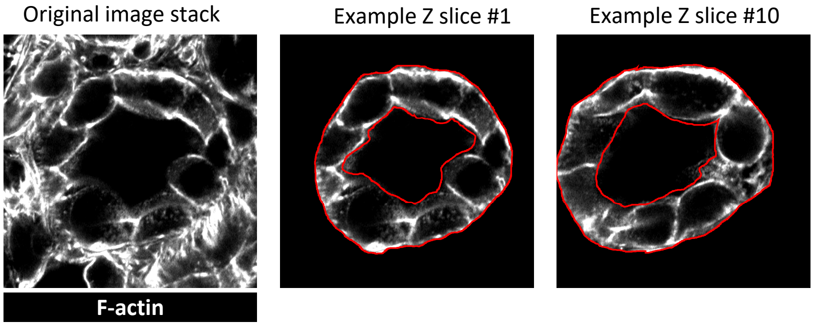

- create ROI around tubule, mask outside
- exclude inside lumen
- max intensity projection every 5 slices
- Obtain Mean Gray Value per projection and take average per stack

B *Exemplary quantification Figure 2G: lateral Ecad intensity and height*

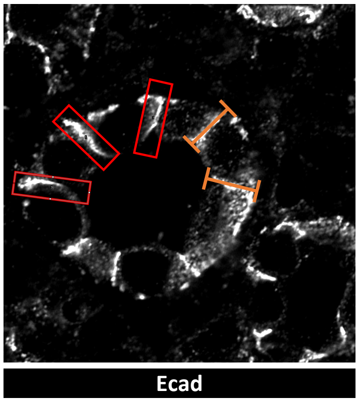

- Tubular cross-section
- Normalize background
- For Intensity:
  - Create **rectangular ROI** around lateral Ecad
  - Mean Gray Value
- For height
  - Line measuring tool in ImageJ/Fiji
  - Measure **apical to basal length**

C *Exemplary quantification Figure 2I,6J: apical F-actin major axis length*

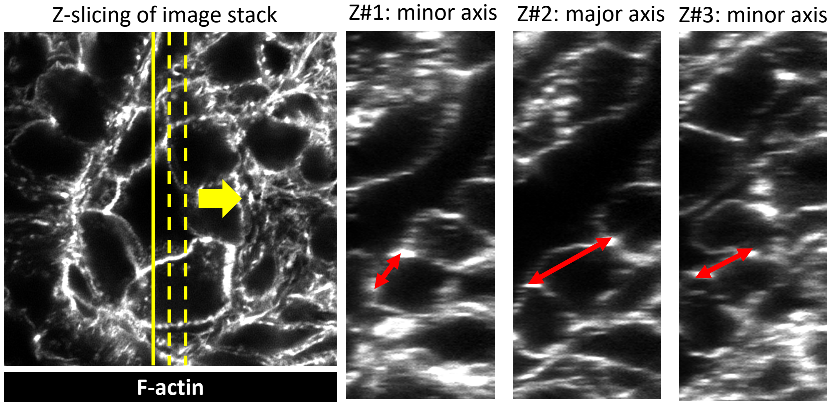

- Z-slicing of tubular cross-section, identify apical F-actin
- Determine the longest longitudinal cellular extension (**major axis**) using the apical F-actin at the cell-cell borders

D *Exemplary quantification Figure 5A: pMLC intensity*

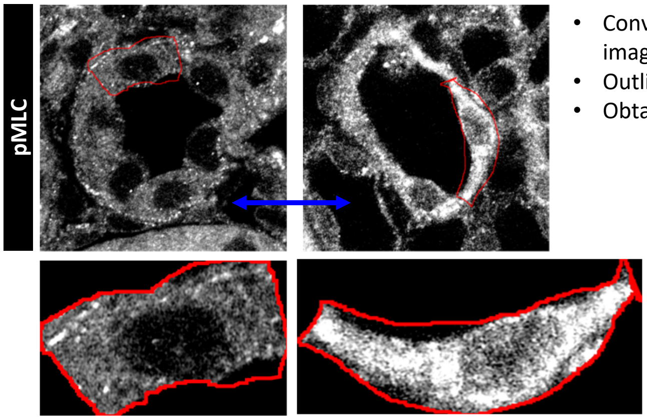

- Convert pMLC to grayscale image, normalize image backgrounds to each other ↔
- Outline single cell using ROI (**in red**)
- Obtain Mean Gray Value per cell

Supplemental Figure 32. (A-D) Explanation of quantifications
